# Angiogenesis as a Therapeutic Target of (Poly)phenols: Tackling Cancer and Vascular‐Related Complications

**DOI:** 10.1002/mnfr.70110

**Published:** 2025-05-15

**Authors:** María Ángeles Ávila‐Gálvez, Antonio Vico‐Padilla, Claus Schneider, Juan Carlos Espín, Antonio González‐Sarrías, Juan Antonio Giménez‐Bastida

**Affiliations:** ^1^ Laboratory of Food and Health, Research Group on Quality, Safety and Bioactivity of Plant Foods Department of Food Science and Technology CEBAS‐CSIC Murcia Spain; ^2^ Division of Clinical Pharmacology Department of Pharmacology Vanderbilt Institute of Chemical Biology Vanderbilt University School of Medicine Nashville Tennessee USA

**Keywords:** cancer, cardiovascular, diabetes, endothelial cells, inflammation

## Abstract

Targeting angiogenesis as a strategy for treating cancer or vascular‐associated complications is an inspiring field for many investigators. An active area within this discipline is the search for agents capable of modulating angiogenesis in order to ameliorate its structural and functional abnormalities associated with these diseases. (Poly)phenols are a broad group of molecules, many of which fall within the category of natural compounds with therapeutic potential. These potential medicinal effects have launched a considerable number of studies investigating the pro‐ and(or) anti‐angiogenic properties of (poly)phenols in different (patho)physiological settings. The purpose of this review is to summarize the current evidence of the role of (poly)phenols in modulating angiogenesis. This review will guide the reader through preclinical and human investigations describing the pro‐ and anti‐angiogenic effects of these compounds in different pathophysiological context, the cellular and molecular mechanisms associated, the key points in the design and evaluation of the effects described, and suggest new approaches to be considered in future studies to overcome the current limitations.

## Graphical abstract

(Poly)phenols constitute a source of natural therapeutic molecules capable of targeting angiogenesis in different scenarios. This review summarizes the current evidence of the role of (poly)phenols in modulating angiogenesis. The reader can find a compilation of preclinical and human investigations describing pro‐ and anti‐angiogenic effects of these molecules in different pathophysiological contexts, along with the main cellular and underlying molecular mechanisms.

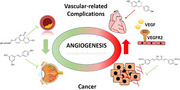

## 1 Angiogenesis

The cardiovascular system maintains the cellular environment and supports survival by delivering oxygen and nutrients, removing waste, and ensuring immune surveillance. Blood vessels also play a critical role in organogenesis, tissue growth, and regeneration through the formation of new blood vessels from pre‐existing ones, a mechanism known as angiogenesis, in which endothelial cells play a critical role [1].

**FIGURE 1:**
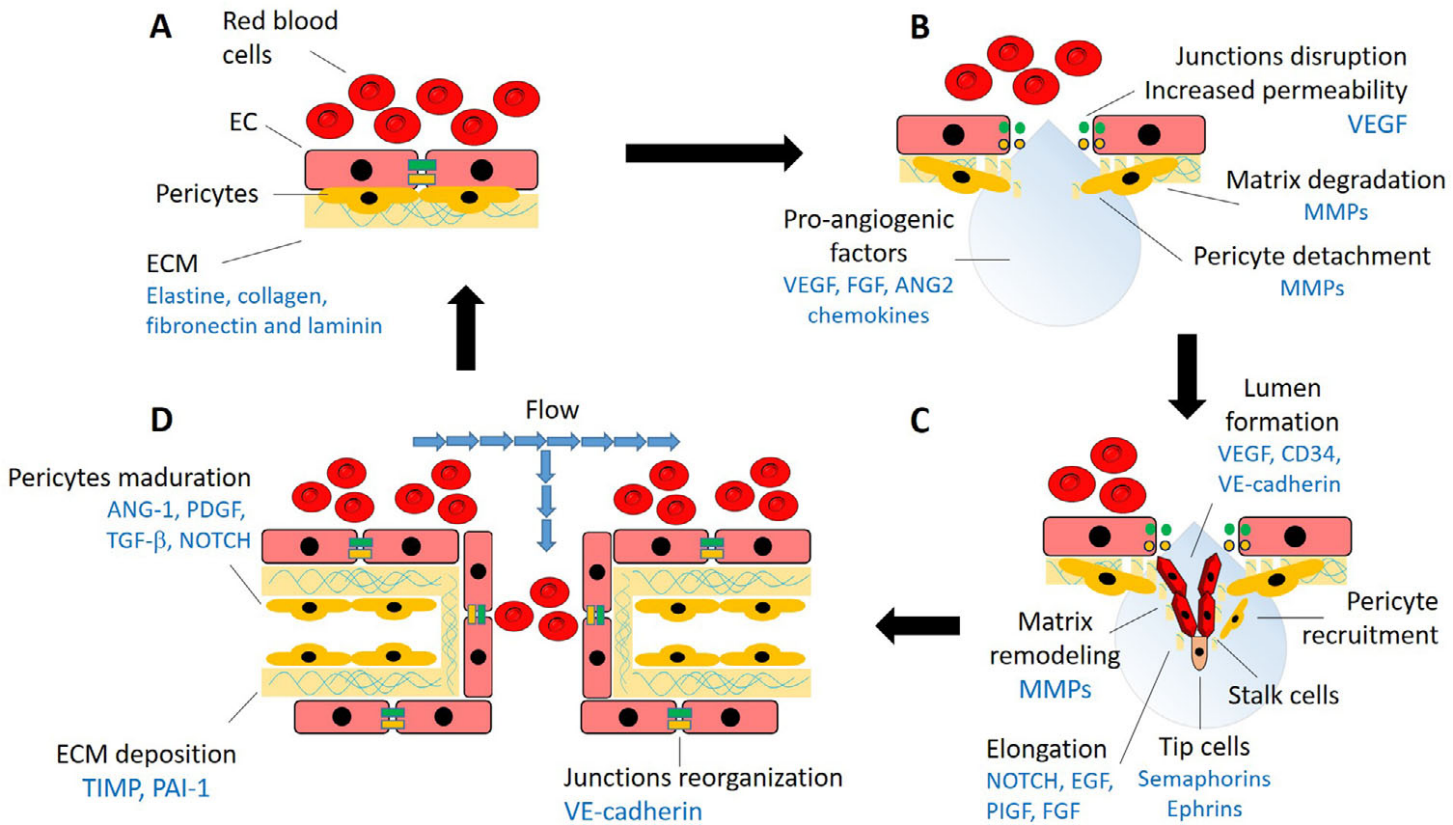
Description of the consecutive steps of blood vessel formation with the cellular and molecular mechanisms involved in each of them. (A) In normal conditions, endothelial cells remain in a quiescent state and contribute to maintaining via interaction with other cell types such as pericytes. (B) Growth factor stimulation induces blood vessel dilation and increased permeability via junction disruption, matrix degradation, and pericyte detachment. (C) The ECM serves as a scaffold for endothelial migration (stalk and tip cells), leading to the formation of the lumen and branches. (D) Branches fusion followed by junction reorganization, ECM deposition, and pericyte maturation results in the formation of functional new blood vessels.

As shown in Figure 1, endothelial cells form an inner lining monolayer of the vessels interconnected by junctional proteins (i.e., claudins and VE‐cadherin) and in contact with other cellular types, such as pericytes. In healthy conditions, endothelial cells remain quiescent, optimizing blood flow, and supplying oxygen and nutrients to the tissues. The increase of the biosynthesis of pro‐angiogenic signals (Table 1), such as, growth factors, angiopoietins, or chemokines induces the proteolytic degradation of the basement membrane (elastin, collagen, fibronectin, and laminin) by the matrix metalloproteinases (MMP; e.g., MMP‐9), detachment of pericytes from the basal wall, disruption of endothelial junctions and increase of vascular permeability (induced by VEGF) [1, 2, 3]. This increased permeability allows the formation of an extracellular matrix (ECM) layer, which serves as a scaffold for endothelial cell migration. At this point, one single endothelial cell, known as the tip cell, migrates onto the extracellular matrix (in response to signal molecules, including semaphorins and ephrins) through the binding of integrins to ligands, including growth factors (VEGF and FGF) and(or) angiopoietins (ANG‐1) [1, 3–6]. Tip cells are followed by the so‐called stalk cells, which are responsible for lumen (modulated by VEGF, VE‐cadherin, and CD34) and elongation/sprouts formation (regulated by NOTCH, PIGF, EGFL7, and FGF) as well as pericytes recruitment. After branches fusion, functional new blood vessels require inactivation of the endothelial cells to the normal quiescent state, reorganization of the cellular junctions, pericytes maturation (ANG‐1, PDGF, TGF‐β, NOTCH), deposition of basement membrane (regulated by anti‐angiogenic factors such as tissue inhibitor of MMP (TIMP) or plasminogen activator inhibitor (PAI)‐1 and maintenance of the pro‐/anti‐angiogenic equilibrium [1, 3–6].

**TABLE 1:** Key molecules involved in angiogenesis.

Molecule	Receptor	Biological effect	References
Growth factors			
VEGF	VEGFR1 VEGFR2	↑Tubulogenesis and endothelial proliferation and migration; ↑vascular permeability; ↑MMP and ECM degradation	[7]
PlGF	VEGFR1 VEGFR2 NRP‐1 NRP‐2	↑Endothelial migration and proliferation	[7]
FGF	FGFR1 FGFR2	↑Endothelial proliferation and migration, capillary morphogenesis, and detachment	[8]
PDGF	PDGFRα PDGFRβ	Modulation of perivascular cell activity and blood vessel maturation	[8]
IFG and IGFBP	IFG1R IFG2R	↑Tubulogenesis and endothelial migration; ↑VEGF biosynthesis and vessel formation	[9, 10]
HGF	c‐Met	↑Endothelial migration, tubular morphogenesis, and branching	[4, 11]
TGF‐β	TβRII, ALK‐5, and the co‐receptor endogline (CD105)	Modulation of VEGF formation and PAI‐1; regulation of migration and proliferation of vascular smooth muscle cells; vascular stability and modulation of the inflammatory response	[8, 12, 13]
EGF	Erb1 Erb2 Erb3 Erb4	↑Endothelial proliferation and differentiation; ↑VEGF biosynthesis	[4]
Angiopoietins			
ANG‐1 ANG‐2	Tie‐2	↑Angiogenesis; ↑vascular proliferation and pericytes shedding; Vascular stabilization and vessel remodeling	[4]
Notch‐Delta/Jagged			
Dll‐1, Dll‐3, Dll‐4, Jag‐1 and Jag‐2	Notch‐1 Notch‐2 Notch‐3 Notch‐4	Modulation of vessel branching, sprouting, and perfusion; tip endothelial cell activation	[1, 4]
Ephrins			
EphrinB2	EphB4	↑Revascularization, sprouting, and vessel maturation; ↑VEGF formation and VEGFR2 activation	[4]

Abbreviations: **ALK‐5**, activin receptor‐like kinase 5; **ANG**, angiogenin; **Dll**, delta like ligand; **ECM**, extracellular matrix; **EGF**, epidermal growth factor; **Eph**, erythropoietin‐producing hepatocellular; **Erb/HER**, human epidermal growth factor receptor; **FGF**, fibroblasts growth factor; **HGF**, hepatocyte growth factor; **IGF**, insulin‐like growth factor; **IGFBP**, insulin‐like growth factor binding protein; **Jag**, Jagged ligand; **MMP**, metalloproteinase; **NRP**, neuropilin; **PDGF**, platelet derived growth factor; **PlGF**, placental growth factor; **VEGF**, vascular endothelial growth factor; **VEGFR**, vascular endothelial growth factor receptor.

Besides the fundamental character of angiogenesis in physiological processes, it also has a prominent role in pathophysiological conditions. Tumor growth is a process derived from the abnormal proliferation of one or a small group of cells, which requires high quantities of oxygen and nutrients. The uncontrolled tumor expansion generates a hypoxic microenvironment characterized by chronic inflammation that triggers the activation of molecular mechanisms to induce the transition from an avascular state (initial phase) to vascularized tumors [8]. In diseases such as atherosclerosis and(or) diabetes‐related complications, diabetic nephropathy, and retinopathy, a range of factors, including lifestyle (diet, exercise, smoking, etc.), genetic makeup, turbulent blood flow and(or) hyperglycemia, leads to vascular dysfunction, production of reactive oxygen species (ROS), formation of advanced glycation end products (AGEs), and activation of their receptors (RAGEs) as well as chronic inflammation (i.e., via NF‐κB activation) and ischemia (reduced blood flow and oxygen level) [14, 15]. Hypoxia triggers neovascularization within these pathological conditions. HIF‐1 is a key transcription factor that, under hypoxic conditions, stimulates the biosynthesis of chemoattractant molecules to recruit pro‐angiogenic cells and up‐regulates the expression of pro‐angiogenic molecules such as ANG, MMP, and growth factors (VEGF, PDGF, and TGF‐β). The overexpression of pro‐angiogenic factors (with a predominant role of VEGF) impairs the equilibrium of pro‐ and anti‐angiogenic molecules, thus promoting an abnormal and chaotic vessel network [1, 16]. NF‐κB also promotes angiogenesis through the stimulation of the biosynthesis of pro‐inflammatory cytokines (TNF‐α, IL‐6, and IL‐1β) and chemokines (IL‐8, CCL‐2), growth factors (VEGF, bFGF), proteases (MMP‐9) or NO in a wide range of cancer and(or) immune cells, contributing to the formation of new blood vessels [17]. This results in an abnormal formation of blood vessels that fuel the growth of cancerous cells (which, in turn, promotes neoangiogenesis), contribute to the growth and instability of atherosclerotic plaques, and generate sight‐threatening hemorrhages (due to vessel fragility) or renal failure.

The role of angiogenesis is strikingly different in defective wound healing and diseases such as myocardial infarction, limb ischemia, or stroke, characterized by inadequate tissue perfusion and formation of ischemic tissues [15]. The repair of the hypoxic zone requires a robust pro‐angiogenic response defined by a marked inflammatory response (immune cells activation, biosynthesis of pro‐inflammatory cytokines), pro‐angiogenic growth factors release (VEGF, TGF‐β, PDGF), or increased levels of MMPs and NO, among others, mediators. However, endogenous angiogenesis might be limited (e.g., due to commonly associated complications such as advanced age, diabetes, or atherosclerosis), thus leading to inefficient remodeling, scarring of the affected tissues, and long‐term undesirable effects [15].

Both excessive and insufficient vessel formation are of major interest regarding anti‐ and pro‐angiogenic therapies. The groundbreaking studies led by Folkman [18] and Isner et al. [19]. brought angiogenesis to the forefront as a target for effective anticancer therapies and(or) a promising strategy against ischemic damage at the cardiovascular level, respectively. Since then, significant progress has been achieved in the search for molecules (i.e., growth factors such as VEGF) that regulate angiogenesis. The study of the molecular mechanisms of blood vessel formation and the identification of therapeutic targets set the basis of current anti‐ and pro‐angiogenic therapies [1, 4, 20, 21]. Despite major pharmacological advances achieved (development of anti‐angiogenic inhibitors or cellular pro‐angiogenic therapies, among others) and the large number of clinical trials, there is still questionable therapeutic efficacy (drug resistance or adverse and off‐target effects) of clinically approved treatments [3, 4]. The limitations of the current anti‐/pro‐angiogenic therapies encourage the search for novel approaches in the modulation (promoting or inhibiting) of the formation of new blood vessels, depending on each physiological context.

An attractive strategy invokes natural compounds as an alternative to the current pharmacological treatments, thanks to their potential for efficacy and reduced toxicity. The pharmacological development of pro‐ and anti‐angiogenic agents usually sets a particular biological target as a starting point to discover efficient “silver bullets.” In contrast to this “first laboratory and then clinic” approach, the research of plant‐derived products often begins with the observation of a relevant pro‐ and(or) anti‐angiogenic effect associated with their consumption [22, 23] followed by the identification of bioactive compounds and the underlying molecular mechanisms [24, 25, 26]; that is, “first in vivo, then in vitro” [27].

## 2 (Poly)phenols as a Strategy to Modulate Angiogenesis for the Treatment of Cancer and Vascular‐Related Complications

### 2.1 (Poly)phenols

Dietary (poly)phenols are naturally occurring compounds that result from the metabolism of plants. According to their chemical structure, (poly)phenols fall within two main categories: flavonoids and non‐flavonoids. Flavonoids contain a characteristic chemical structure with 15 carbon atoms organized in three rings (C_6_‐C_3_‐C_6_), in which two phenolic rings are bound by a heterocyclic ring. The hydroxylation pattern and oxidation state of flavonoids defines different sub‐classes of compounds including flavonols (rutin, quercetin), flavanones (naringin, hesperidin), isoflavones (daidzein, genistein), flavan 3‐ols (epicatechin, epigalocatechin), flavones (apigenin, fisetin), and anthocyanidin (cyaniding, delphinidin) [28]. Non‐flavonoids show a higher structural variability and encompass a variety of sub‐classes: phenolic acids (caffeic acid), hydrolysable tannins (ellagitannins and ellagic acid), stilbenes (resveratrol), curcuminoids (curcumin), and lignans (secoisolariciresinol). Flavonoids and non‐flavonoids are molecules abundant in many fruits and vegetables (detected as conjugated with other molecules such as sugar moieties), including coffee, soy products, olive oil, and pomegranate, among others [29]. Their regular intake through diet results in the presence of the (poly)phenols in the organism, justifying the interest in studying their benefits. A considerable number of comprehensive and highly cited manuscripts have appeared on the study of (poly)phenol chemical structure, metabolism, biological activity, and effects on human health [30, 31, 32, 33]. This review covers the impact of (poly)phenols on angiogenesis modulation and addresses fundamental questions usually overlooked: how relevant is the design of (pre)clinical studies to draw conclusions from the results? Are phase‐II metabolites able to modulate angiogenesis? Are these circulating metabolites stable molecules or precursors of biologically active molecules? Have new molecules (e.g., microbial‐derived metabolites) with the capacity to modulate angiogenesis been identified? Have new underlying mechanisms been described?

### 2.2 Search Strategy

PubMed was the database selected for a comprehensive literature search regarding the effect of (poly)phenols on angiogenesis. The search terms used were: (flavonoid/non‐flavonoid OR specific (poly)phenol sub‐class OR individual molecule) AND angiogenesis‐related terms (angiogenesis OR angiogenic) AND pathophysiological context such as cancer (cancer OR tumor OR carcinogenesis), vascular‐related complications (cardiovascular disease OR atherosclerosis OR atherosclerotic), or diabetes (diabetes OR diabetes complications OR diabetes mellitus).

The combination of the different terms was adapted for each (poly)phenol sub‐class (e.g., flavanones or isoflavones) and its individual compounds (e.g., naringenin, naringin and hesperetin, or daidzein and genistein, respectively) along with each pathophysiological context (cancer, vascular complications, or diabetes) in order to ensure a more targeted and extensive literature review. The search was carried out from January to October 2024 by the authors and provided 4041 studies published over the years with no time restriction. The eligibility criteria included: (i) all (pre)clinical investigations focused on the study of (poly)phenols on angiogenesis and its associated (patho)physiological processes; (ii) dietary context (studies using direct injection/administration of (poly)phenols were excluded); (iii) in vitro investigations designed according to physiological realistic conditions such as concentrations detected in vivo, appropriate cellular models and relevant molecular forms. Book chapters, reviews, modified (not natural) (poly)phenols, retracted, and non‐English studies were excluded.

The following sections summarize the studies that were in line with the eligibility criteria. Independent tables (Tables 2 and 3 as well as Tables S1 and S2) show the current evidence of the role of angiogenesis as a target of (poly)phenols.

**TABLE 2:** Human studies describing effects on angiogenesis associated with the consumption of (poly)phenols.

Population of the study	Study design	Foodstuff; Intake and duration	Main outcomes	References
Healthy individuals				
Non‐smoking healthy males (*n* = 4)	Quasi‐experimental (within‐subject control), placebo‐controlled, crossover	Experimental group: Blackcurrant powder supplemented with Quer (30 mg/day), provided in sachets, and diluted in 400 mL drink water Placebo group: blackcurrant flavored (containing synthetic flavor, aspartame, sodium citrate, citric acid, and sugar) Duration: consumption of 400 mL placebo for 2 weeks (all participants) followed by the consumption of 400 mL Quer‐enriched drink for 2 additional weeks (total 4 weeks)	No effect on MMP‐2 or TIMP‐2 and ↑TIMP‐1 (gene expression and protein level in plasma)	[46]
Healthy males (*n* = 47)	Protocol 1: cross‐over, randomized, single‐center, double‐blind, acute 3‐arm (*n* = 12) Protocol 2: randomized, single‐center, double‐blind, parallel, cross‐over, 4‐arm study (*n* = 24) Protocol 3: single‐center, randomized, 2‐arm cross‐over study (*n* = 5) Protocol 4: single‐center, randomized, double‐blind, cross‐over (*n* = 6)	Protocol 1: consumption of drinks containing 820 mg* CF (125 mg (‐)‐epicatechin*) and(or) 122 mg* Mx (together or individually); acute consumption at 3 different days (7‐day wash‐out between each consumption day). Parameters measured at time 0 (baseline) and after 2 h Protocol 2: consumption of increasing amounts of CF (from 102 to 820 mg*) and(or) Mx (from 61 to 244 mg*); acute consumption (alone or together) in each visit (8 or 10 total visits). Parameters measured at time 0 (baseline) and after 2 h Protocol 3: acute consumption of drinks containing 820 mg CF*(125 mg (‐)‐epicatechin*) alone or together with 122 mg Mx*. Parameters measured at time 0 (baseline) and after 1, 2, 3, 4, and 5 h Protocol 4: acute consumption of 75 mg (‐)‐epicatechin alone or with 400 mg theobromine and 26 mg caffeine. Parameters were measured at time 0 (baseline) and after 1, 2, and 4 h, and urine was collected for 24 h	↑FMD after CF intake (highest increase observed in the presence of Mx); no effect on FMD after consuming Mx alone; ↓bPWV and DBP, while ↑CACs (CD34^+^/KDR^+^) after intake of CF alone (highest increase observed in the presence of Mx); ↑plasma (‐)‐epicatechin metabolites level in the presence of Mx (similar results observed in the group consuming pure (‐)‐epicatechin together with theobromine and caffeine)	[43]
Healthy male volunteers (*n* = 42) participating in an overfeeding trial	Placebo‐controlled, randomized, volunteer‐blind (identical capsules), parallel design	Experimental group: capsules containing 250 mg grape extract (daily intake of 2 g grape (poly)phenols) Placebo group: identical‐looking capsules Duration: daily intake for 28 days	No adverse effects; down‐regulation in adipose tissue of 41 genes associated with angiogenesis compared to the placebo group; ↓PECAM1/CD31 in adipose tissue in the experimental group after intervention	[47]
Cancer patients				
Patients with high‐risk oral premalignant lesions (*n* = 41)	Phase‐II, randomized, placebo‐controlled	Experimental group: green tea extract (0.5, 0.75, and 1 g/m)^a^ administered in capsules (THEA‐FLAN 30 ARG) Placebo: capsules Duration: 3 capsules per day for 12 weeks	Higher clinical response observed in the experimental group (with evidence of dose‐dependent effects at higher doses) compared to placebo; Improvement of histological results (not significant); correlation found between histologic response and never drinkers in one patient; no difference in oral cancer‐free survival between the experimental and placebo groups; no association between green tea intervention and oral cancer development; Well tolerated (Grade 4 toxicity not reached), but higher doses induced insomnia, gastrointestinal symptoms and(or) nervousness (grade 3 toxicity), leading to discontinuation in 2 patients; No differences between experimental and placebo groups regarding hematologic results; EGCG detected at concentrations close to 1 µmol/L with a high variability in the 0.5 g/m^2^ (1.7–75.3 ng/mL; *n* = 3) and 1 g/m^2^ (5.7–435.2 ng/mL; *n* = 3) groups; correlation between baseline stromal VEGF score and better clinical response in the experimental group (VEGF is a good predictor for clinical response); lack of differences in the modulation of biomarkers between the different doses tested; lower up‐regulation of VEGF in the overall experimental group compared to placebo; ↓VEGF and CD1 level in clinical responders compared to non‐responders within the experimental group (not significant);	[48]
Prostate cancer patients (*n* = 145)	Randomized controlled (non‐placebo) trial, non‐crossover, parallel‐group design	Experimental group: Phase‐II trial with 4 arms: (i) usual diet, (ii) low‐fat diet, (iii) FS‐enhanced diet, and (iv) FS + low‐fat diet (30 g FS/day with or without low‐fat diet) Duration: 30–31 days	↓Eotaxin and IP‐10 (FS alone); ↑IL‐8; ↓Eotaxin, MIP‐1α, SCF, β‐NGF, and CA‐9 level (FS + low‐fat); No significant reduction angiogenic factors (e.g., VEGF), NF‐κB, and COX‐2	[49]
Patients suffering CAD (*n* = 16)	Randomized, controlled, crossover, double‐blind	Experimental group: High‐flavanol cocoa (375 mg cocoa flavanols) and low‐flavanol cocoa (9 mg cocoa flavanols) Duration: twice daily for 1 month	Comparative effects of the high vs. the low period: 47% improvement of the FMV of the brachial artery; ↓SBP and ↑plasma nitrite level; ↑2.2‐fold CD34^+^/KDR^+^‐CACs; no difference in the capacity of the CACs to migrate, proliferate, differentiate, or survive	[50]
Prostate cancer patients (*n* = 48)	Randomized, double‐blind, placebo‐controlled trial	Experimental group: Polyphenon‐E (enriched green tea polyphenol extract; 85%–95% total catechins, 56%–72% EGCG, and less than 1% caffeine) oral capsules (containing 200 mg EGCG per capsule) Placebo group: matched placebo capsules Duration: 4 capsules each morning for 3–6 weeks before surgery	Low level of green tea (poly)phenols detected in prostatectomy tissue (range of pmol/g) or plasma (pmol/mL); favorable but not significant effect on PSA; no effect on IGF‐1 or IGFBP‐3 pathway or oxidative damage in blood leukocytes; non‐significant reduction of Gleason score; no effect on cell proliferation (Ki‐67), apoptosis induction (cleaved caspase‐3), and microvessel density (CD34)	[42]
Patients (*n* = 10) with locally advanced non‐inflammatory breast cancer undergoing radiotherapy	Randomized, placebo‐controlled, parallel‐group design	Experimental: EGCG (400 mg in 2 capsules with 0.1 L water) and radiotherapy Placebo group: only capsules and radiotherapy Duration: 2 capsules, 3 times per day for 5 weeks	↓Serum VEGF and HGF as well as MMP‐2 and MMP‐9	[36]
Premenopausal women (*n* = 9) with normal breast tissue	Quasi‐experimental intervention (within‐subject control), non‐randomized, and non‐placebo controlled	Intervention group: Ground FS‐enriched diet: 25 g per day Duration: ∼30 days	↑Endostatin level in breast tissue; no effect on angiogenin or VEGF level	[51]
Patients (*n* = 34; 21–65 years) with resected Stage I–III ER‐ and PR‐ breast cancer with no active disease	Randomized, phase IB, double‐blinded, placebo‐controlled, and dose‐escalation study	Intervention group ( *n* = 26): consumption of Polyphenon E (green tea extract containing EGCG) capsules to intake 800 mg (*n* = 14), 1200 mg (*n* = 11), and 1600 mg (*n* = 1) EGCG Placebo group ( *n* = 8): matching placebo Duration: 2–4 capsules daily for 6 months	Significant transient decrease of serum HGF (only after 2 months of treatment) in EGCG consumers. No significant effects on VEGF, serum Ch and TG, oxidative damage, and inflammatory biomarkers	[35]
Pre‐surgical trial in postmenopausal women with ductal carcinoma in situ or primary invasive Stage I or II (*n* = 28)	Controlled, parallel‐group design	Experimental: Consumption of 3 Green tea capsules (Pro Health Green Tea Mega EGCG, 725 mg per capsule containing ∼314 mg EGCG) per day (equivalent to ∼8–10 cups of green tea) Control: No capsules provided (*n* = 18) Duration: 35 days before surgery (*n* = 13)	No significant effect in apoptosis (caspase‐3) and angiogenesis (CD34) within and between the groups; ↓Ki‐67 level within the green tea group (significant in benign cells) and compared with the control group	[52]
Prostate cancer patients (48–78 years) with high risk of recurrence (*n* = 159)	Double‐blind, randomized, placebo‐controlled	Experimental group ( *n* = 81): soy protein isolate beverage powder (23.8 mg GEN, 15.0 mg DAZ, and 41 mg total isoflavones) Placebo group ( *n* = 78): caseinate‐based product Dose and duration: Daily consumption of a beverage powder (47 g) enriched with soy protein (19.2 g) or placebo (19.8 g) for 18 months	↓Serum SHBG and circulating testosterone (but not free testosterone); ↓testosterone:estradiol ratio; estradiol concentration unaffected by soy supplementation; no effect on serum VEGF, bFGF, Fas, and Fas‐ligand (and their ratio) or IGF‐1 and IGFBP‐3 (and their ratio)	[53, 54]
Patients with vascular‐related disorders				
CAD patients (*n* = 16) and healthy young (*n* = 12) and old (*n* = 12) volunteers	Randomized, controlled, cross‐over, double‐blind	Intervention group: High‐flavanol cocoa (375 mg cocoa flavanols) and low‐flavanol cocoa (9 mg cocoa flavanols) Duration: twice daily for 1 month	↓CD144^+^ and CD31^+^/41^‐^ endothelial microparticles; no effect on CD41^+^ platelet‐derived microparticles or Factor Xa (microparticles associated with pro‐coagulant activity)	[55]
Non‐treated hypertensive patients (*n* = 19; 7 males and 12 females)	Randomized, double‐blind, controlled, cross‐over study	Intervention: Sachets diluted in 100–200 mL hot water to obtain black tea (150 mg (poly)phenols) Placebo: caffeine, tea flavor, caramel color, and sucrose Dose and duration: twice per day in 2 periods (13 days wash‐out) of 8 days, each one	↑Functionally active CACs and FMD compared with placebo; attenuation of the effect of cream (fat) consumption on FMD impairment; no significant effect on biomarkers of low‐grade inflammation (CRP, SAA, IL‐1β, IL‐6, IL‐8, TNF‐α) and endothelial dysfunction (sICAM, sVCAM, E‐sel, ET‐1); no effect on TCh, LDL, HDL, TG, glucose or insulin level in plasma	[56]
Patients diagnosed with primary chronic venous disease (*n* = 38) classified as CEAP2, 3, and 4	Quasi‐experimental intervention, prospective, open‐label, uncontrolled	Intervention group: 600 mg diosmin Dose and duration: twice daily consumption for 3 months	↓Edema and average leg circumference; pain amelioration; no effect on biochemical blood parameters; ↓VEGF‐A (highest reduction in CEAP4), VEGF‐C, TNF‐α, IL‐6, FGF2 and ↑angiostatin (higher increase in CEAP2 and 3); no effect on plasminogen	[57]
Patients diagnosed with unilateral choroidal neovascularization related to AMD (*n* = 50)	Multicenter, parallel, randomized, observer‐blinded trial	Formulation 1 (intervention): Vit C, Vit E, Zinc, manganese, selenium, copper, hydroxytyrosol, RSV, zeaxanthin, lutein, and DHA Formulation 2 (control): Vit C, Vit E, Zinc, manganese, selenium, and β‐carotene Dose and duration: 2 capsules daily for 12 months (with evaluations at baseline and 6 months)	↑Lutein and zeaxanthin levels in the intervention group; ↑DHA, n‐3 PUFAs, and total n‐3 LCPUFAs compared with the control group; ↓n‐6 PUFAs, n‐6 LCPUFAs, and the n‐6/n‐3 PUFAs and LCPUFAs; ↓INF‐γ, IL‐1β, IL‐8, TNF‐α in the intervention group; ↓MMP‐10 and VEGF levels in both groups (no difference between the groups); adverse effects related to the eyes reported	[58]
Patients diagnosed with primary chronic venous disease (*n* = 47; average age = 44 years) classified as CEAP2, 3, and 4	Quasi‐experimental intervention, prospective, open‐label, uncontrolled	Experimental group: 600 mg diosmin Dose and duration: Twice daily consumption for 3 months	↓Creatinine (CEAP3) and TG (CEAP4); ↓leg circumference and VAS; no effect on BMI; ↓contraction incidence; ↓lactate, HIF and AG; ↑endostatin and angiostatin; ↓FGF23, VEGF‐A, VEGF‐C and FGF; ↓TNF‐α, IL‐1β, IL‐6 and D‐dimer (fibrin degradation product); ↑IL‐12; no effect on CRP	[59]
Patients with reproductive and hormone disorders				
Patients diagnosed with polycystic ovary syndrome (*n* = 61)	Randomized, triple‐blind, placebo‐controlled, intervention study	Intervention group: RSV capsules (800 mg/day) Placebo group: identical capsules (provided by the same company) Duration: twice daily (2 × 400 mg per capsule) for 40 days	Intragroup differences: ↓AMH, LH, (intragroup significant difference in both groups); ↓total testosterone and ↑TSH (intragroup significant difference only in the intervention group); ↑estradiol, progesterone, FSH, prolactin, (intragroup significant difference in both groups) Intergroup differences: ↑TSH, FSH, and ↓LH and total testosterone Other outcomes: no effect on the fertility, cleavage, and fertilization rates or the number of mature oocytes; ↑high‐quality embryo and oocyte rates; ↓*VEGF* and *HIF1* genes (mRNA)	[60]
Infertile patients with endometriosis (*n* = 34)	Randomized, placebo‐controlled, parallel, double‐blind	Intervention group: RSV (400 mg; Mega RSV, Southampton, UK) Placebo group: tablet containing maltodextrin and lactose (Mega RSV, Southampton, UK) Duration: twice daily (2 × 200 mg per capsule) for 12–14 weeks (together with oral contraceptives, the last 3 weeks)	No adverse effects associated with RSV consumption; ↓VEGF and TNF‐α levels (gene and protein) after the intervention (within the same group) and compared with the control group	[61]

Abbreviations: **(LC)PUFA,** (long chain) polyunsaturated fatty acid; **AG,** anion gap; **ALA,** α‐linoleic acid; **AMD,** age related macular degeneration; **b.w.,** body weight; **bFGF,** basic fibroblasts growth factor; **BMI,** body mass index; **bPWV,** brachial pulse wave velocity; **CA‐9,** carbonic anhydrase‐9; **CAC/EPC,** circulating angiogenic cells/endothelial progenitor cell; **CAD,** coronary artery disease; **CD31/PECAM‐1,** platelet endothelial cell adhesion molecule; **CEAP,** clinical‐etiology‐anatomy‐pathophysiology; **CF,** cocoa flavanols; **CHD,** coronary heart disease; **COX‐2,** cyclooxygenase‐2; **CRP,** C‐reactive protein; **DBP,** diastolic blood pressure; **DHA,** docosahexaenoic acid; **EGCG,** epigallocatechin gallate; **EPA,** eicosapentaenoic; **EPC/CAC,** endothelial progenitor cell/circulating angiogenic cells; **ER,** estrogen receptor; **E‐sel,** E‐selectin; **ET‐1,** endothelin‐1; **FGF,** fibroblast growth factor; **FMD,** flow‐mediated vasodilation; **FMV,** flow‐mediated vasodilation; **FS,** flaxseed; **GSH,** glutathione; **HDL,** high density lipoprotein; **HIF,** hypoxia‐inducible factor; **IGF,** insulin like growth factor; **IGFBP,** insulin growth factor binding protein; **IL,** interleukin; **IP‐10,** interferon‐γ‐inducible protein 10; **KDR (VEGFR2),** vascular endothelial growth factor receptor 2; **LDL,** low density lipoprotein; **MIP‐1α,** macrophage inflammatory protein 1 alpha; **MMP,** metalloproteinase; **Mx,** methylxanthines; **NGF,** neurotrophic growth factor; **PECAM‐1/CD31,** platelet endothelial cell adhesion molecule; **PR,** progesterone receptor; **PSA,** prostate‐specific antigen; **Quer,** quercetin; **RSV,** resveratrol; **SAA,** serum amyloid A; **SBP,** systolic blood pressure; **SCF,** stem cell factor; **SHBG,** sex hormone‐binding globuling; **sICAM‐1,** soluble intercellular adhesion molecule 1; **sVCAM,** soluble vascular adhesion molecule 1; **TCh,** total cholesterol; **TG,** triglyceriedes; **TIMP,** tissue inhibitor of metalloproteinase; **TNF‐α,** tumor necrosis factor alpha; **VAS,** visual analog scale; **VEGF,** vascular endothelial growth factor.

*Quantity administered to a 75‐kg person.

^a^ The dose was based on the body surface area of the participants.

**TABLE 3:** Effects of (poly)phenols in cellular and molecular mechanisms related to angiogenesis in cell‐based assays.

Cellular model	Metabolite(s) assayed	Dose/duration	Outcomes	References
Urolithins				
HAEC	Co‐treatment with 50 ng/mL TNF‐α and Uro‐A glur, Uro‐B glur, Uro‐A, and Uro‐B	0.1–15 µmol/L; 4–12 h	↓THP‐1 monocyte adhesion and endothelial migration (Uro‐A glur and Uro‐A); ↓IL‐8 (only Uro‐A), CCL‐2 (Uro‐A glur and Uro‐A) and PAI‐1 level (only Uro‐A glur)	[186]
HAEC	Uro‐A, Uro‐B, Uro B‐glur, and their mixture	15 µmol/L of each compound and 5 µmol/L of each in the mixture (15 µmol/L final concentration); 5 min and 24 h	↑NO production (only the mixture after 24 h) and eNOS level (only Uro B‐glur and the mixture after 5 min); slight deconjugation of Uro B‐glur and sulfation of the individual compounds	[187]
Flavanones				
HAEC	Co‐treatment with 50 ng/mL TNF‐α Hesp 3‐glur, Hesp 7‐glur, Hesp 3‐sulf, Hesp 7‐sulf, Hesp	0.1–10 µmol/L; 12–24 h	Phase‐II metabolites (10 µmol/L) and Hesp (10 and 1 µmol/L) reduced endothelial migration Hesp 3‐glur, Hesp 7‐sulf (10 µmol/L) and Hesp (0.1–10 µmol/L) reduced PAI‐1 level	[188]
Isoflavones and derived metabolites				
HAEC	Pre‐treatment with GEN, GEN 7‐glur, GEN 4’‐glur, DAZ, DAZ 4’‐sulf, DAZ 7‐glur, DAZ 4’‐glur, Eq, and Equation 7‐glur followed by stimulation with a mixture of growth factors or 100 ng/mL VEGF_165_	0.1–10 µmol/L; 4–24 h	No effect on endothelial proliferation; ↓tubulogenesis by GEN, DAZ, Equation (0.1–10 µmol/L) and Equation 7‐glur (only 10 µmol/L); ↓endothelial migration (only Eq and Equation 7‐glur); ↓p‐VEGFR2/t‐VEGFR2 (GEN and Eq), p‐ERK/t‐ERK (Eq, Equation 7‐glur and GEN) and p‐Akt/t‐Akt (GEN and DAZ); no effect on bFGF pathway; binding affinity of Eq and GEN to VEGFR2 active site (in silico); no effect on VEGFR2 location; dissimilar conjugation/deconjugation metabolism in the different assays	[161]
Curcuminoids				
HIMEC	Pre‐treatment with Curc followed by VEGF (50 ng/mL) or TNF‐α (100 U/mL)	0.1–20 µmol/L; 0.5–2 h pre‐treatment with Curc and up to 72 h in co‐treatment with VEGF	↓Proliferation, endothelial migration and tubulogenesis; ↓COX‐2 (mRNA and protein level) and PGE_2_ and 6‐keto PGF1α (dose‐dependent); ↓p‐JNK/t‐JNK, p‐ERK/t‐ERK and p‐p38/t‐p38 (similar effect exerted by specific inhibitors); ↓U‐937 leukocyte adhesion; ↓ICAM‐1, VCAM level; ↓E‐selectin level (not significant)	[189]
HIMEC	Treatment with Curc in the presence or absence of activated platelets or 25 µmol/L ADP	0.625–40 µmol/L; 4–24 h	No cytotoxic effects at concentrations up to 10 µmol/L; ↓VEGF level, cell invasion and tubulogenesis (dose‐dependent); ↓α‐SMA, E‐cadherin and collagen I (dose‐dependent); no effect on VEGFR; ↓p‐PI3K/t‐PI3K, p‐Akt/t‐Akt, p‐mTOR/t‐mTOR, HIF‐1α level; ↓HIF1α nuclear translocation; no significant effect on LamB1	[190]
Flavonols				
BPCVEC and PAEC	Treatment with Quer, Quer 3‐glur, and Quer 3’‐sulf	1 × 10^−3^–1 × 10^3^ nmol/L; 0.5–48 h	No cytotoxic effects observed; ↑Cell growth by Quer 3’‐sulf (no effect of Quer and Quer 3‐glur) at 1 and 10 nM in the absence of VEGF; ↓Cell growth by Quer (40% at 1 nmol/L) and Quer 3‐glur (10 nmol/L–1 µmol/L), whereas Quer 3’‐sulf lacked effect (from 1 nmol/L to 1 µmol/L) in the presence of VEGF; ↓Cell migration by Quer and Quer 3‐glur (no effect of Quer 3’‐sulf); ↑p‐ERK/t‐ERK by 10 nM Quer 3’‐sulf in absence of VEGF; ↓p‐ERK/t‐ERK by 10 nmol/L Quer 3‐glur in the presence of VEGF; Quer 3’‐sulf target VEGFR2 and downstream pathways such as PI3K/Akt and cGMP	[180]
RIMEC	Treatment with Quer alone (12 h) or followed by 10 µg/mL LPS (12 h)	2.5–160 µmol/L; 12–24 h	No effect on cell viability or LDH release at concentrations up to 80 µmol/L (Quer alone); ↓VCAM‐A and ICAM‐1 level (dose‐dependent effect); ↓p‐NF‐κB p65, t‐NF‐κB p65, TLR‐4, MyD88, p‐ERK, p‐JNK, p‐p38, p‐STAT, and ↑I‐κB level (at 80 µmol/L)	[191]
RIMEC	Treatment with kaempferol alone (12 h) or followed by 10 µg/mL LPS (6 or 12 h)	6.25–200 µmol/L; 3–15 h	No effect on cell viability at concentrations up to 100 µM (Quer alone); ↓TNF‐α, IL‐1β, IL‐6, TLR‐4, ICAM‐1, and VCAM‐1 level (dose‐dependent); ↓p‐NF‐κB p65/t‐NF‐κB p65, p‐I‐κB/t‐I‐κB and p‐STAT/t‐STAT ratio; no effect on p‐p38/t‐p38 ratio	[192]
RIMEC	Pre‐treatment with kaempferol followed by stimulation with LPS (1 µg/mL) and TNF‐α (20 ng/mL)	25–100 µmol/L; 6–48 h	No effect on cell proliferation; ↓IL‐6 and ↑IL‐10 (dose‐dependent effect); ↓p‐p65/p65 (protein), *Il‐6* and *Rela*/*p65* gene expression (mRNA); ↓VEGFR2, p‐Akt/t‐Akt, VEGFA, HIF‐1α, p‐p38/t‐p38 and p‐HSP27, eNOS, bFGF level (similar effect exerted by p38 and Akt inhibitors); ↓*Vegfr2* and *Vegfa* gene expression (mRNA); ↓cell migration and tubulogenesis (similar effect exerted by p38 and Akt inhibitors); ↓RIMEC barrier permeability and ↑TEER; ↑ZO‐1, occludin‐1 and ↓claudin‐2; ↓CD31 level	[193]

Abbreviations: **6‐keto PGF1α**, 6‐keto prostaglandin F1α; **ADP**, adenosine diphosphate; **Akt**, protein kinase B; **bFGF**, basic fibroblast growth factor; **BPCVEC**, Bovine post‐capillary coronary venular endothelial cells; **CCL‐2/MCP‐1**, monocyte chemoattractant protein 1; **CD31/PECAM‐1**, platelet endothelial cell adhesion molecule 1; **cGMP**, cyclic guanosine monophosphate; **COX‐2**; **Curc**, curcumin; cyclooxygenase‐2; **DAZ**, daidzein; **eNOS**, endothelial nitric oxide synthase; **Eq**, equol; **ERK**, extracellular signal‐regulated kinase; **GEN**, genistein; ‐**Glur**, glucuronide; **HAEC**, human aortic endotelial cells; **Hesp**, hesperetin; **HIF‐1α**, hypoxia inducible factor 1; **HIMEC**, human intestinal microvascular endotelial cells; **HSP27**, heat shock protein 27; **HUVEC**, human umbilical vein endotelial cells; **ICAM‐1**, intercellular adhesion molecule 1; **IL**, interleukin; **JNK**, c‐Jun N‐terminal kinases; **LamB1**, laminin subunit beta 1; **MCP‐1/CCL‐2**, monocyte chemoattractant protein 1; **mTOR**, mammalian target of rampamycin; **My88D**, myeloid differentiation primary response 88; **Nar**, naringenin; **NF‐κB**, nuclear factor kappa‐light‐chain‐enhancer of activated B cells; **p‐**, phosphorylated; **PAEC**, porcine aortic endothelial cells; **PAI‐1**, plaminogen activator inhibitor 1; **PECAM‐1/CD31**, platelet endothelial cell adhesion molecule 1;**PGE_2_**, prostaglandin E2; **PI3K**, phosphoinositide 3‐kinase; **Quer**, quercetin; **RIMEC**, rat intestinal microvascular endotelial cells; **STAT**, signal transducer and activator of transcription; **Sulf**, sulfate; **TEER**, trans epithelial electrical resistance; **TLR‐4**, toll‐like receptor 4; **TNF‐α**, tumor necrosis factor alpha; **Uro**, urolithin; **VCAM,** vascular cell adhesion molecule; **VEGF**, vascular endothelial growth factor; **VEGFR**, vascular endothelial growth factor receptor; **ZO‐1**, zonula occludens‐1.; **α‐SMA**, alpha smooth muscle actin.

### 2.3 Evidence From Human Studies

Table 2 summarizes 20 human studies published between 2001 and 2023 (studies published before 2001 or after 2023 did not fit within the eligibility criteria set) that investigated the phenotypical effects and the modulation of angiogenesis‐related markers in healthy volunteers or patients (cancer and vascular‐related complications) to determine the effect of the consumption of plant‐derived products rich in (poly)phenols or their individual compounds. Four studies in healthy volunteers evaluated the impact of different natural products (flaxseed, grape extracts, cocoa flavanols, or quercetin‐enriched drinks) on molecules involved in angiogenesis modulation (growth factors, MMP, etc.), endothelial cells related to cardiovascular health [34] and regulation of molecular mechanisms (i.e., gene and protein expression) in plasma and systemic tissues (e.g., adipose and breast tissues). The significant effects observed in cellular (CD31/PECAM‐1 and CD34^+^/KDR^+^) and anti‐angiogenic (i.e., TIMP‐1 and angiostatin) markers provide information about possible mechanisms of prevention against chronic disease development via modulation of angiogenesis. Whether this modulation is also observed in a pathological framework is addressed in fourteen further studies. Polyphenon‐E (green tea extract) and epigallocatechin gallate (EGCG) showed the capacity to reduce the level of pro‐angiogenic molecules (VEGF, HGF, and MMP‐2 and ‐9) in breast cancer patients [35, 36] that could imply an anti‐angiogenic role of these natural compounds. Nevertheless, these findings are inconsistent with the lack of effect on the level of pro‐angiogenic molecules (i.e., VEGF or IGF/IGFBP3) or microvessel formation described in additional studies using breast and prostate cancer patients (Table 2). Hence, the modest anticancer effects (clinical effects or improvement of symptoms) of consuming (poly)phenol‐containing products appear independent of angiogenesis regulation (inconsistent effects on markers and mediators). Regarding vascular‐associated conditions (e.g., chronic venous diseases or hypertension), the angiogenesis‐related circulating endothelial cells (CAC) and growth factors (angiopoietins or VEGF, among others) act as angiogenic targets through which (poly)phenol‐rich products might exert their effects against vascular disorders.

Despite the evidence provided in these studies, the significance of the results is scant due to several factors that deserve attention. An important point is the low number of studies (only 17 included in Table 2). Limited funding for natural product research (especially compared to pharmaceuticals) [37, 38] is a potential barrier to increasing this number, and, in turn, it restricts the duration and size of studies. Thus, most of the studies included in Table 2 were only a few weeks in duration and included fewer than 50 treated patients, yielding, in most cases, no significant effects. Longer (e.g., 12–18 months) and larger enrolment numbers testing the effects of (poly)phenols on angiogenesis are desirable to obtain relevant results. Placebo is also a pertinent design component to consider when conducting these studies. The absence of a placebo‐control group [39] limits interpretation of the data and decreases the robustness of the conclusions of a study [40]. The optimization of the placebo composition is another issue to consider due to its importance to blind studies [41] and reduce off‐protocol interferences such as the presence of (poly)phenols of interest in the intervention and placebo‐control group [42]. Identifying the circulating molecular forms of dietary (poly)phenols produced after their consumption in plasma or systemic tissues is another point that requires attention. Only two studies analyzed the concentration of flavanols‐derived compounds achieved in plasma, urine, and tissue samples from healthy [43] and prostate cancer patients [42] and analysis occurred following pre‐treatment with deconjugating enzymes. This approach may lead to incomplete hydrolysis of the conjugates and an underestimation of exposure to these molecules [44], thus making the definition of effective concentrations in plasma and tissues, as well as the identification of molecular mechanisms and significant targets, more complex. These studies lacked information on the concentrations of phase‐II metabolites achieved (data are shown as free forms), even though sulfates and glucuronides are the major circulating metabolites, with free forms (non‐conjugated) being near absent [45]. Information on the systemic concentration of phase‐II metabolites is important in identifying relevant bioactive compounds (and the molecular forms). The conjugates might exert a direct effect on processes related to angiogenesis (Table 3) or act as precursors of their free forms (that are, in general, more active than their conjugates) via deconjugation, for example, carried out by endothelial cells under specific conditions (see Section 2.4). Also relevant to consider is that most of the preclinical studies have been performed using the free forms, a major limitation when trying to extend mechanisms and insight from in vitro to in vivo studies, where conjugates (poly)phenols dominate.

### 2.4 Evidence From Animal Models

There is a clear need for more clinical trials specifically designed to determine whether the effects of dietary interventions with natural products on chronic disease risk are, at least partly, mediated by their impact on angiogenesis. Clinical trials are significantly enhanced when mechanistic information and biomarkers from well‐designed pre‐clinical studies, particularly in vivo, are available [26, 62].

Non‐clinical in vivo evidence provides information about the therapeutic effects of particular plants through the modulation of angiogenesis. Cancer animal models (prostate, ovarian, or breast cancer) fed diets enhanced with extracts of traditional medicinal plants, including *Ocimun gratissimum* [63], *Emblica officinalis* [64], *Gnetum gnemon* seed [65], or *Punica granatum* [66, 67] showed anticancer effects associated with the inhibition of angiogenesis, whereas Xuefu Zhuyu capsules consumption [68] promoted new vessel growth in a myocardial infarction model. Microvessel density (measured as CD34 or CD31), VEGF, MMPs, and(or) HIF‐1α were common processes and molecular mechanisms associated with the effects observed, yet the magnitude of their modulation was model dependent.

A critical point regarding these studies resides in pinpointing the bioactive molecule(s) and the underlying molecular mechanisms. Notwithstanding, the complex composition of these extracts challenges the identification of bioactive compounds relevant to the modulation of angiogenesis and the critical molecular targets [26]. The chromatographic characterization and fractionation of botanical extracts with the capacity to promote [69], attenuate uncontrolled angiogenesis [70], or exert anti‐angiogenic effects [71, 72, 73, 74, 75, 76, 77, 78], targeting key biomarkers (VEGF, VEGFR2, bFGF, or survivin), in diabetic and cancer animal models, highlight (poly)phenols as constituents that most likely mediated the biological effects observed. Innovative approaches such as network pharmacology [79] or biochemometrics [80] are attractive methodologies to correlate the metabolic profiles of natural products with biological activity. Hence, studies using these methods support the role of dietary (poly)phenols as effective molecules in modulating a range of physiological processes, including angiogenesis, and predicting their potential molecular targets [81]. The information obtained from these methodologies requires further confirmation coming from “more complex” non‐clinical in vivo biological assays, which encompass other biological factors (e.g., gut microbiota, metabolism, bioavailability, and metabolic activation) that are critical to understanding how (poly)phenols target angiogenesis [41, 82, 83]. Tables S1 and S2 summarize a number of important animal studies focused on investigating the potential of (poly)phenols consumption (dietary approach) in murine models as a non‐invasive, cost‐effective, and accessible dietary strategy to modulate angiogenesis in different pathophysiological backgrounds.

A joint phenotypic effect observed in cancer animal models consuming different (poly)phenols, alone or in a mixture, is a reduction in the formation of blood vessels in tumors through the decrease of CD31, CD34, and(or) von Willerbrand factor (vWf) level (markers of endothelial proliferation and vascular density). This effect, along with the antiproliferative and pro‐apoptotic responses, is associated with tumor growth inhibition in different cancer models (Table S1). The mechanistic information described in Table S1 places VEGF as the main target of the (poly)phenols tested, and its biosynthesis inhibition as a key antiangiogenic effect. A contrasting trend to this effect comes from the studies using isoflavone‐enriched diets. Genistein exemplifies this controversial since its consumption might increase [84, 85, 86], reduce [87], or exert no effect [88, 89] on VEGF level (together with variable effects on microvascular density) in different cancer animal model, highlighting the need of future studies evaluating dose‐, time‐ and context‐dependent effects. Beyond the inhibitory effects on VEGF level, (poly)phenols such as EA [90], EGCG [91, 92, 93, 94, 95], sylibin and silibinin [96, 97], Quer [98], or Curc [99], among others, exert a consistent reduction of the receptors VEGFR1, VEGFR2, and VEGFR3 expression at the protein and mRNA level. At the receptor level, the mechanism of VEGFR2 inhibition depends on the specific (poly)phenol tested. EGCG and rhamnazin inhibit the phosphorylation of VEGFR2 at Tyr^1175^ and Tyr^951^, respectively, thereby inhibiting downstream related pathways such as MAPK, Akt, and STAT3 [92, 100]. These results reinforce the role of the VEGF route as a crucial pathway of these natural compounds in tumor angiogenesis. However, other less approached growth factors with a recognized role in blood vessel development (see Section 1) are also important markers regulated by (poly)phenols. The IGF/IGF1R is another important pathway [101] regulated at different points by the consumption of different (poly)phenols in animal models. Thus, diets enriched in green tea, and its main (poly)phenol EGCG, are associated with a reduction of IGF‐I and higher level of IGFBP3 (molecule involved in the regulation of the IGFs activity) [101] together with lower level of IGF1R (total and phosphorylated) [102, 103]. RSV and Quer are also capable of down‐ and up‐regulating IGF‐I and IGFBP5 gene expression, respectively, while increasing IGFBP7 protein level [104]. The effect of the consumption of (poly)phenols on other important growth factors (bFGF, PIGF, PDGF, HGF, EGF, and TGF‐β) are investigated in less detail, describing an increase or reduction in their level (tissue or serum) and their relation with the pro‐ and anti‐angiogenic effects observed (Table S1).

The inhibition of the master switch HIF‐1α [105] is a crucial mechanism related to the antiangiogenic (lower growth factors level and their receptors inactivation) and anticancer effects exerted by (poly)phenols. All studies included in Table S1 describe a downregulation of HIF‐1α (at mRNA and protein level) regardless of the cancer animal model, (poly)phenol consumed, dose administered, or duration of the study. This inhibitory effect is likely to involve the regulation of interconnected signaling pathways. For example, curcumin [99, 106, 107] and silibinin [97, 108–111] administration exert a reduction of MMP‐2 and MMP‐9 levels (its activation results in the degradation of ECM and release of bound VEGF) [112], which may explain the lower levels of VEGF/VEGFR via modulation of the HIF‐1α/mTOR/VEGF/VEGFR route. Akt and ERK (information on p38 and JNK is scant) are further relevant signaling pathways consistently inhibited by all tested (poly)phenols included in Table S1, with EGCG being the most documented example in hepatocarcinoma, intestinal, and pancreas cancer models [91–94, 102].

The antiangiogenic effects observed in the cancer animal models are also concomitant with an anti‐inflammatory effect exerted via inhibition of the NF‐κB pathway and related molecules (COX‐2, PGE_2_, IL‐6, TNF‐α, and IL‐1β). Curcumin stands out as the most widely tested molecule, with up to 10 studies supporting its inhibitory effect on the NF‐κB pathway (Table S1). The study by Tian et al. illustrates this effect, describing a reduction of TLR4/NF‐κB pathway along with low levels of IL‐6, IL‐1β, PGE_2_, COX‐2, VEGF, and CD31 [113].

While the inhibitory effect of (pol)phenols on angiogenesis in cancer animal models is rather consistent, their effects in vascular‐related complication animal models are context‐dependent and less clear at the phenotypical and molecular level, see Table S2.

The (poly)phenols investigated can exert dissimilar phenotypical effects on angiogenesis in the same pathophysiological model. For instance, taking as a reference the diabetic models, the effect on angiogenesis can be pro‐ (flavanones, resveratrol, and curcumin) [114, 115, 116, 117, 118, 119, 120], anti‐angiogenic (flavones) [121, 122], or lack effect (sylimarin) [123]. A further nuance to this is the specific tissue effect within the same model as observed in kidneys and left ventricles (low and high CD31 level, respectively) of animals fed a high‐fat diet in the presence of XN and 8‐PN [124]. Specific chemical structure, dosage, and bioavailability are factors that warrant consideration to understand these effects. The specific pathological context of the model tested is also relevant in molecules such as resveratrol, which can promote angiogenesis in hind limb ischemia [125], reduce blood vessel formation in cerebral ischemia [126], or have no effect in myocardial ischemia models [127]. Otherwise, the effect of one specific compound can be dependent on the animal model investigated. Curcumin acts as a pro‐angiogenic molecule in diabetes and ischemia [118, 128], whereas in a thoracic aneurysm model, it exerts opposite effects [129].

In the vascular context, the VEGF/VEGFR system is the critical pathway that determines the effect of (poly)phenols on the formation of new blood vessels. Representative examples (from Table S2 containing 86 studies) show that dietary administration of (‐)‐epicatechin [130] or trilobatin [131] stimulates angiogenesis via VEGF/VEGFR2 activation (as evidenced by increased VEGF synthesis and receptor level and phosphorylation), whereas compounds such as scutellarin [122] and cyanidin‐3‐*O*‐β‐glucoside [132] inhibit angiogenesis through suppression of VEGF/VEGFR signaling (including VEGFR1 and VEGFR2 reduction). This dual modulation (VEGF formation and receptor activation), in turn, involves VEGF‐mediated signaling pathways such as Akt, ERK, or p38 [124, 133], whose activation or inhibition is concomitant with the effects observed on the VEGF/VEGFR pathway. Further mechanisms involved in the activation of the VEGF/VEGFR pathway by (poly)phenols imply important transcription factors, including HIF‐1α and NF‐κB. The HIF‐1α activation after the consumption of (‐)epicatechin, along with higher MMP‐2 and MMP‐9 levels, could increase the VEGF formation and VEGFR2 activation, leading to higher capillary density [134]. Nevertheless, its role is less clear in other compounds such as curcumin (enhanced angiogenesis, but reduced *Hif1a* gene expression) [118] and Quer (HIF‐1α reduction results in contrasting effects on VEGF‐A/VEGFR2 activation) [135]. The effect on NF‐κB activation also depends on the (poly)phenols investigated. In this regard, RSV and HHC induced its activation (concomitant with higher VEGF and VEGFR2 level) [136, 137], whereas apigenin, Quer, and polydatin exerted an inhibitory effect (in line with lower VEGF synthesis) [138, 139, 140]. A likely link to explain this correlation between NF‐κB and VEGF levels resides in inflammatory molecules derived from the NF‐κB pathway [141]. This mechanism is supported by a few studies describing that Quer [140] and cyanidin‐3‐*O*‐β‐glucoside [132] consumption exerted a reduction of NF‐κB, VEGF and TNF‐α, IL‐6, IL‐1β, and IL‐18 levels, while HCC [137] increased NF‐κB and TNF‐α levels.

The role of angiogenesis as a target of (poly)phenols constitutes an exciting frontier in cancer or vascular treatments. The identification of the VEGF/VEGFR, HIF‐1α and NF‐κB pathways as key targets of (poly)phenols and the consistent effects of (poly)phenols (particularly in cancer studies) across a wide variety of approaches regarding the animal models (xenografts, knock‐out or genetically modified, diabetic or myocardial infarction, among others) and the administration strategy (individual compound or their mixtures in extracts or food products) lend credence to the role of these natural compounds as effective regulators of angiogenesis. The challenge at this point is to identify the molecular form of the active principle(s), which is a demanding task based on the dynamic metabolism of these molecules and a better understanding of the underlying mechanisms.

### 2.4 Dietary (Poly)phenols as Precursors of Gut Microbial and(or) Derived Metabolites: Identifying Active Principles in Angiogenesis Modulation

**FIGURE 2:**
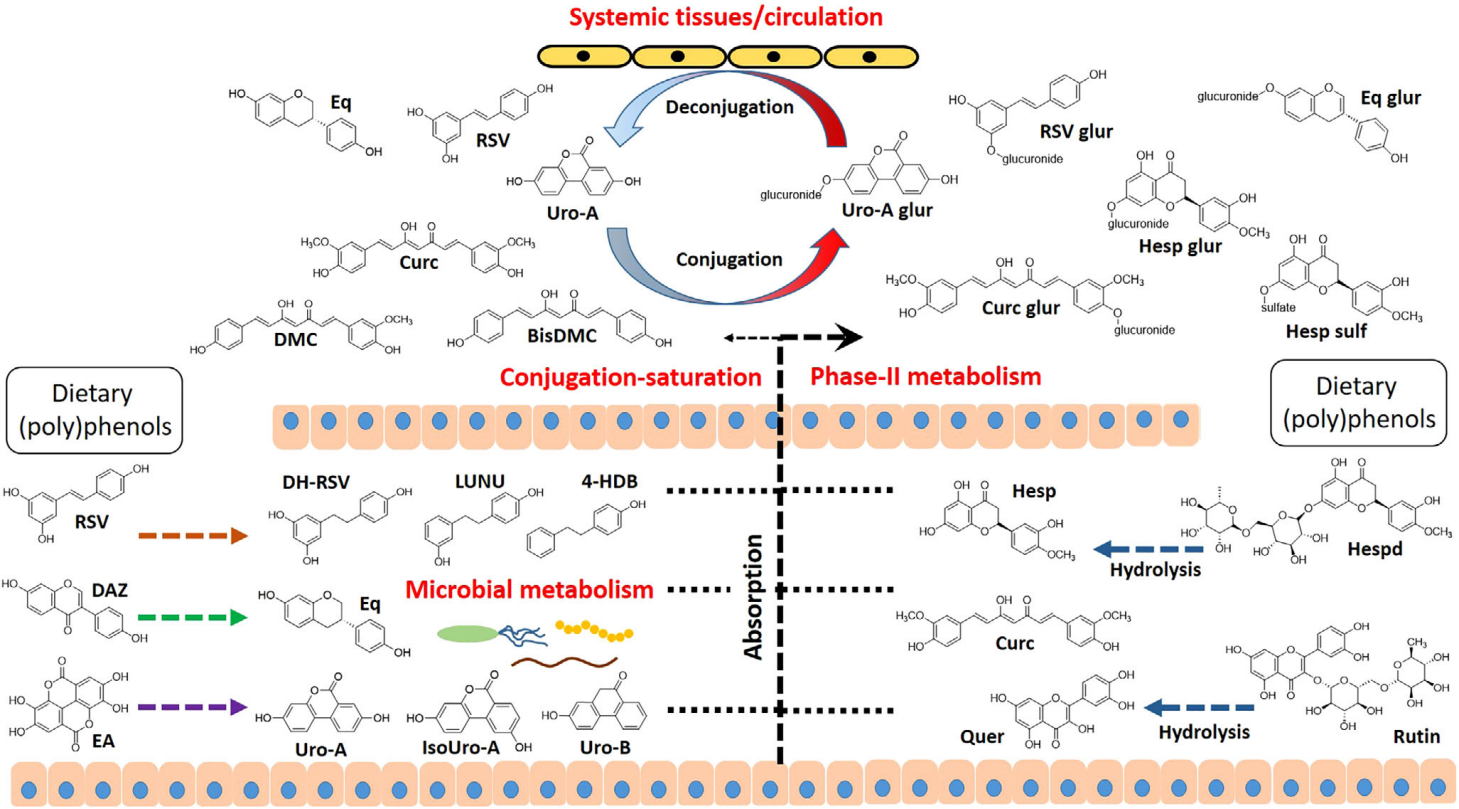
Illustration of the complex metabolite fate of dietary (poly)phenols. The metabolism of dietary (poly)phenols, at the intestinal level, determines whether these molecules reach the gastrointestinal tract in their original form (i.e., glycosides) followed by further metabolism (e.g., hydrolysis), or undergo microbial metabolism resulting in the biosynthesis of derived metabolites (equol, urolithins, LUNU, 4‐DHB). Conjugation with sulfate or glucuronic acid is the major pathway of Phase‐II metabolism of these compounds, which are circulating molecules that might enter a conjugation/deconjugation cycle via interaction with other cells (i.e., endothelial cells). The conjugation‐saturation mechanism is a minor pathway that contributes to explain the presence of dietary (poly)phenols (such as curcuminoids), when consumed in a (poly)phenolic cocktail, in the bloodstream and systemic tissues.

The documented in vivo effects observed in most studies reviewed above were clearly associated with the dietary (poly)phenol(s) consumed. Nevertheless, it is difficult to rationalize their effects considering the near absence of the parent (glycosylated) compounds or their free forms in the bloodstream and systemic tissues. These compounds show a low absorption in the small intestine (restricting their distribution in systemic tissues in their original form) and reach the gastrointestinal tract in their native structure (conjugated with sugar moieties or in their free form). As illustrated in Figure 2, the (poly)phenols can undergo different metabolic routes by interacting with the gut microbiota or intestinal enzymes at the intestinal level. Hence, ellagitannins and ellagic acid (present in walnuts or pomegranate), isoflavones (genistein and daidzein) or resveratrol (RSV) act as precursors of microbial metabolites known as urolithins (Uro) [142], equol and *O*‐desmethyl‐angolesin (ODMA) [143], or lunularin (LUNU) and 4‐hydroxy‐dibenzyl (4‐HDB) [144], respectively. Deglycosylation of quercetin 3‐rutinoside (rutin) results in the release of quercetin [145], while curcumin and flavanones (e.g., hesperidin and hesperetin) are detectable in the intestinal lumen for hours in their native form, reaching concentrations from µmol/L to mmol/L [146, 147]. Upon absorption, conjugation with glucuronic acid and(or) sulfate is the major Phase‐II metabolic pathway, and the derived products, glucuronides, and(or) sulfates are the major molecules detected (i.e., Uro‐A glucuronide, equol 7‐glucuronide, RSV 3‐glucuronide, or curcumin glucuronide) at the systemic level at concentrations from nmol/L to low µmol/L [148]. Mechanisms such as conjugation‐saturation reactions may constitute an alternative path to the conjugation route, allowing the detection of the free forms (e.g., curcuminoids) administered with a cocktail of phenolics in systemic tissues [149]. Deconjugation of phase‐II metabolites is another proposed mechanism of (poly)phenol metabolism. The circulating molecules (glucuronides and sulfates as major compounds) constitute a source of free moieties formed via deconjugation, such as luteolin [150, 151, 152], Uro‐A [153], quercetin [154, 155, 156, 157, 158, 159], resveratrol [160], equol [161], or curcumin [162, 163]. These free forms, in turn, can experience further reductive or oxidative metabolism (only described in vitro) to form bioactive molecules (oxidative metabolites of curcumin is a paradigmatic example) [164, 165], which can be relevant to rationalizing their biological activity (Figure 2).

The metabolism of (poly)phenols constitutes the basis to design relevant in vitro studies: intestinal cells treated can be with free forms, while systemic cellular types (from endothelium, liver, breast tissue, etc.) should be treated with conjugated molecules using concentrations achieved in vivo. Under some circumstances such as inflammation, in addition to the phase‐II metabolites, the use of free forms can also be relevant at the systemic level due to their release from the conjugated forms [153, 158, 162]. Nevertheless, the reduced biological activity described for phase‐II metabolites (compared with their counterparts) and the often lack of availability of these complex but relevant molecules are key drawbacks behind the lack of physiological relevant in vitro studies [27], making it difficult to draw conclusions about the active principle(s) in the modulation of angiogenesis.

### 2.5 (Poly)phenols and Endothelial Cells: Cellular and Molecular Mechanisms of In Vitro Angiogenesis Modulation

Research on angiogenesis and (poly)phenols in vitro is another key element of the intriguing equation that integrates, besides cell‐based assays, human studies, animal experimentation, bioavailability, metabolism, and natural products´ composition [37]. All these elements are part of an interconnected system in which in vivo assays determine the physiological conditions to design relevant cell‐based studies. At the same time, the in vitro results provide indispensable mechanistic information to refine the current in vivo preclinical and human strategies. Hence, the adherence of in vitro studies focused on angiogenesis modulation by (poly)phenols to the standards of excellence established elsewhere [27] is crucial to maintaining the equilibrium between the elements of the equation described above.

Cell line selection is one of the most important variables to consider in the accomplishment of reliable in vitro results [27]. There is a broad consensus about the role of endothelial cells as a reference model to investigate relevant cellular and molecular mechanisms in angiogenesis. One fundamental feature of endothelial cells that researchers do not always acknowledge is their tissue‐specific characteristics and vascular heterogeneity [166, 167, 168]. Human umbilical vein endothelial cells (HUVEC), an extensively used cell type isolated from fetal tissue, exhibit significant cellular and molecular differences compared to adult vascular endothelium cells (which resemble generic, functional, and morphological characteristics of the tissue of origin), such as human saphenous vein endothelial cells (HSVECs) [169] and human aortic endothelial cells (HAEC) [170]. Depending on the (patho)physiological research context, the use of human intestinal microvascular endothelial cells (HIMEC) [171, 172, 173], HSVEC [169], or HAECs [174] might be suitable models for angiogenesis investigation in intestinal (e.g., intestinal inflammation or colorectal cancer) or vascular (e.g., atherosclerosis) studies. In contrast, HUVEC could be more relevant in gestational vascular development research [175]. There are also differences between primary and immortalized human endothelial cells, for instance, HAECs and EA.hy 926 (hybrid between HUVEC and A549 cells) [176] or HUVEC and ECV304 (spontaneously transformed from HUVEC) [177]. While immortalized endothelial cells are a useful tool in angiogenesis and (poly)phenols research [178, 179], primary endothelial cells are more representative of an in vivo physiological context [27]. Studies combining primary and immortalized endothelial cells could enable a balance between physiological relevance and reproducibility in angiogenesis investigation as a target of (poly)phenols. Besides human cells, in light of the higher number of animal studies compared to human trials, primary endothelial cells from animal models [180, 181] are also an attractive and helpful instrument to understand better the interaction (poly)phenols/endothelial cells in angiogenesis at the cellular and molecular level in non‐clinical in vivo studies. A further important factor to consider regarding the primary cell lines is the health status of the donor. Comparison of human retinal endothelial cells (HREC), HUVEC, and HAEC from healthy and diabetic patients exemplifies the importance of this concept because of the notable differences exhibited in relevant cellular and biological pathways (e.g., TGF‐β) linked to vascular‐related complications [182, 183, 184]. Translation of preclinical findings into clinical settings can be more reliable when the studies consider the (patho)physiological origin of the cells. In the case of diabetic retinopathy studies, for example, using HREC from healthy and diabetic donors might be more appropriate than using only normal HREC [132, 185] to test the angiogenic effects of (poly)phenols. A third crucial variable to contemplate resides in the physiological‐like assay conditions. Incorporating metabolism and bioavailability information (molecular form, intestinal and systemic concentration, and exposure time) in in vitro designs is key to overcoming the limited physiological relevance of studies based on questionable experimental conditions [27]. Given the limitations to obtaining and working with primary endothelial cells and relevant (poly)phenol metabolites (intricate isolation procedures, reduced cellular half‐life, low availability of metabolites, elevated costs), there is limited evidence coming from well‐designed in vitro studies (Table 3).

Design elements should align with the physiological conditions of the research context (e.g., intestinal vs. systemic level). Intestinal‐isolated endothelial cells treated with dietary (poly)phenols and(or) unconjugated forms at plausible in vivo concentrations achieved in the gastrointestinal tract provide reliable insights into the effects of these compounds on angiogenic mechanisms. In this regard, the in vitro anti‐angiogenic effects exerted by curcuminoids and flavonols in HIMEC and RIMEC [189, 190] (Table 3) are consistent with the in vivo chemopreventive findings described in colon cancer models [194] (Table S1). At the systemic level, the design of the assays can be more challenging since the circulating conjugated compounds are the major metabolites detected. However, unconjugated metabolites and dietary‐free forms can also be present due to deconjugation or conjugation‐saturation processes (see Section 2.4). For this reason, a comparative strategy using single and(or) mixtures of representative (un)conjugated molecules to treat endothelial cells can mimic in vivo conditions and can provide a better comprehension of the effects exerted by these molecules. The studies included in Table 3 reveal a bidirectional interaction between the primary endothelial cells and (poly)phenols. While phase‐II metabolites and their free forms modulate the cellular and molecular response of the endothelial cells (Table 3), these molecules undergo endothelial (de)conjugation [161, 187, 188]. This in vitro evidence adds a layer of complexity to the studies of biological activity and raises interesting questions of whether the circulating metabolites exert direct effects via interaction with key targets (VEGF/VEGFR2 pathway) or go through metabolic transformation to form active metabolites. This underscores the need to implement endothelial metabolism in preclinical in vitro and in vivo studies to identify the active principle(s) and their target(s) involved in angiogenesis modulation.

## 3 Conclusions and Roadmap

There is evidence that (poly)phenols constitute a source of natural therapeutic molecules capable of targeting angiogenesis in different scenarios. More than 300 preclinical (in vivo) studies included in this review (Tables S1 and S2) describe how different groups of (poly)phenols exert chemoprevention or amelioration of vascular‐related ischemic disorders, promoting or inhibiting the formation of new blood vessels, depending on the physiological context. Despite the considerable pre‐clinical effects described, we are still far from establishing compelling proof about the active principle(s) identity and the underlying molecular mechanism(s) in a dietary context. In this regard, several important points deserve special attention:

Preclinical and clinical in vivo investigations require better‐designed studies. As stated above, the low number of human and clinical trials and less than optimal design are important factors behind the lack of conclusive evidence to establish an unequivocal link between the interaction of (poly)phenols/angiogenesis and associated beneficial effects. The number of animal studies included in this review is much higher, yet the evidence for some specific group of (poly)phenols (e.g., anthocyanins, flavanones, or ellagitannins) is still scarce. High‐quality clinical trials and preclinical (in vivo) research facing the known challenges in their design are critical to finding answers to fundamental questions and gaining health benefits.

In vitro methodologies need to be improved to meet new requirements. It is clear that primary endothelial cells have a higher relevance than commercial cell lines [27]. Considering the heterogeneity of the cells and the (patho)physiological context of the investigation will help to refine the in vitro design and obtain results closer to the in vivo scenario. Parallel in vivo and in vitro assays [135, 178, 195, 196] pave the way for bidirectional improvements of the experimental design (i.e., in vivo metabolism of (poly)phenols to set in vitro design; application of mechanistic in vitro information in animal studies), identification of similarities and discrepancies in cellular and molecular mechanisms, and description of effects in different biological systems. In addition to endothelial cells, the study of how (poly)phenols target other cellular models (involved in angiogenesis modulation) using monocultures [197], co‐cultures [198], or even 3D cultures and organoids (not approached yet) will provide a broader perspective of their effects on angiogenesis modulation.

(Poly)phenols can be more than single molecules. Traditional in vitro strategies focused on stable metabolites (detected in the intestine, plasma, or urine) overlook the possible role of these compounds as precursors of biologically active molecules. This raises the question of whether we are looking at the right molecule(s). Endothelial cell metabolism in studies of the biological activity of (poly)phenols is a critical element that defines the chemical structure of the molecules and their bioactivity [199]. Compounds formed via (de)conjugation (free forms vs. Phase‐II metabolites) or through oxidative reactions deserve attention in angiogenesis modulation.

Unprecedented molecular mechanisms should be studied. VEGFR2 is a mainstay in the inhibition (cancer treatment) or promotion (ischemic disorders) of angiogenesis, and the VEGF/VEGFR2 is the major signaling pathway targeted by (poly)phenols (Tables S1 and S2). The newly discovered 5‐lipoxygenase (5‐LOX)/COX‐2 cross‐over hemiketal eicosanoid HKE_2_ [200, 201] also enhances the activation of the receptor and promotes in vitro and in vivo angiogenesis [200, 202]. Targeting the HKE_2_/VEGFR2 link is an unexplored mechanism that offers a new concept to understand the role of (poly)phenols on angiogenesis.

The gut microbiota may be a key player. The gut microbiota is critical to understanding the biological activity of (poly)phenols on angiogenesis [203]. Still, little evidence exists about the effect of the interaction between (poly)phenols and gut microbiota on angiogenesis [204]. Addressing this complex interaction is essential to determine the identity of the main driver(s) (i.e., gut microbiota, (poly)phenols, or both) of the effects associated with the consumption of natural products.

Searching for novel (poly)phenols with angiogenic activity. The formation of the microbial metabolites, lunularin and 4‐HDB, is relevant since it allows the stratification of the population based on their dissimilar capacity to produce them (resveratrol metabotypes, i.e., producers vs. non‐producers) [144]. Information on their biological activity is an unexplored field. Particularly, the characterization of 4‐HDB and its possible derived metabolites (glucuronides and sulfates can be anticipated) as a modulator of angiogenesis will enlarge (i) the inventory of microbial metabolites with angiogenic capacity; (ii) our knowledge from physiological (anti‐ vs. pro‐angiogenic agent) and mechanistic perspectives of a new group of microbial metabolites, which could be relevant in future personalized therapies [148].

## Supporting information



Supporting information

Supporting information

## Data Availability

Data sharing is not applicable to this article as no new data were created or analyzed in this study.
