# Supplementary material for: Angiogenesis as a Therapeutic Target of (Poly)phenols: Tackling Cancer and Vascular‐Related Complications
Source: Mol Nutr Food Res. 2025 May 15;69(15):e70110. doi: 10.1002/mnfr.70110 (PMC12319488; doi:10.1002/mnfr.70110)
Supplement: Supplementary file 1 — Supporting information [file MNFR-69-e70110-s001.pdf]

Angiogenesis as a therapeutic target of (poly)phenols: tackling cancer and vascular-related complications

María Ángeles Ávila-Gálvez[1,*], Antonio Vico-Padilla[1], Claus Schneider[2], Juan Carlos Espín[1], Antonio González-Sarrías[1], Juan Antonio Giménez-Bastida[1,*]

[1]Laboratory of Food and Health, Research Group on Quality, Safety and Bioactivity of Plant Foods, Department of Food Science and Technology, CEBAS-CSIC, P.O. Box 164, 30100 Campus de Espinardo, Murcia, Spain

[2]Division of Clinical Pharmacology, Department of Pharmacology, and Vanderbilt Institute of Chemical Biology, Vanderbilt University Medical School, Nashville, Tennessee 37232, United States

Corresponding authors: Juan Antonio Giménez-Bastida[1,*], PhD (jgbastida@cebas.csic.es) and María de los Ángeles Ávila-Gálvez[1,*], PhD (mavila@cebas.csic.es); Telf. (+34) 968396200 Fax: (+34) 968396213

Table S1. Animal studies describing the effect of dietary phenolics-rich extracts or individual compounds

Animal model (related disease)	Extract/phenolic compound	Dose; duration	Main outcomes	Effect on angiogenesis	Reference
			**Anthocyanins**		
♂ F344 rats (4 – 5 weeks) treated with NMBA (esophagus cancer model)	Frozen dried BRB, anthocyanin fraction, or residue fraction	<u>BRB:</u> 5% (w/w) enriched diet (containing 3.8 μmol g^{-1}); 30 weeks<br><br><u>Anthocyanin fraction:</u> 3.8 μmol g^{-1}; 30 weeks<br><br><u>Residue fraction:</u> 0.02 μmol g^{-1}; 30 weeks	No effect on body weight (during first 25 weeks); ↓body weight in animals consuming BRB (last 5 weeks); ↓papilloma number and volume; ↓cell proliferation (Ki-67); ↑apoptosis in papilloma; ↓p42/44 level and Bcl-2/Bax ratio; ↓COX-2 and PGE$_2$ level; ↓NF-kB p50 nuclear translocation; ↓CD45 level	↓CD34, VEGF and HIF-1α protein level	Wang et al., 2009[1]
♀ Immunodeficient BALB/c nude mice (35 – 40 days) MDA-MB-453 xenograft (breast cancer model)	Anthocyanin-rich extract from black rice (in the presence or absence of VEGF)	100 mg kg^{-1} day^{-1} (p.o.); 28 days	↓Tumor growth and proliferation; ↓uPA (protein level)	↓Microvascular density; ↓MMP-2 and MMP-9	Hui et al., 2010[2]
♂ F344 rats (3 – 5 weeks) treated with NMBA (esophagus cancer model)	Frozen dried BRB, anthocyanin fraction, and protocatechuic acid	<u>BRB:</u> 6.1% (w/w) enriched diet (3.8 μmol g^{-1}); 35 weeks<br><br><u>Anthocyanin fraction:</u> 3.8 μmol g^{-1}; 35 weeks	No effect on body weight; ↓tumor growth; ↓CCL-5, IL-4, IL-6, TNF-α and IL1β and ↑IL-5, IL-12, IL-17A, IL-18, GM, CSF, INF-γ, CCL-3 and IL-10 (in serum); ↓IL1β, ↑IL-12 and no effect on IL-10;	↓Microvascular density (CD34) and serum VEGF	Peiffer et al., 2016[3]

		Protocatechuic acid: 500 ppm; 35 weeks	↓$CD68^{+}CD163^{-}$ macrophages migration into dysplastic lesions and esophageal papillomas; ↓$CD68^{+}$ macrophages trafficking and neutrophil accumulation into NMBA-treated esophageal tissues		
BALB/c mice 4T1 xenograft (breast cancer model)	Anthocyanins isolated from dark-sweet cherry	Equivalent to 150 mg cyanidin 3-glucoside kg^{-1} day^{-1} (gavage); 1 week	No effect on tumor volume; ↓tumor weight; ↓*Cenpf* expression (mRNA)	↓Angiogenesis area (H&E)	Silveira-Rabelo et al., 2022[4]
**Ellagitannins, ellagic acid, and derived metabolites**					
♀ BALB/c athymic nude mice injected with MDA-MB-231 xenograft (breast cancer model)	PGG	10 mg kg^{-1} day^{-1} (p.o.); 36 days	↓Tumor growth and metastasis, Ki-67, p-STAT;↑apoptosis	↓VEGF and CD34 level	Lee et al., 2011[5]
BALB/c nu/nu mice (4 – 6 weeks) injected with PANC-1 cells (prostate cancer model)	EA	40 mg kg^{-1} day^{-1} (gavage) once daily; 5 days a week for 6 weeks	No effect on body weight or toxicity in the liver, spleen, and intestine; ↓Tumor growth, cell proliferation (PCNA and Ki-67), Bcl-2, cyclin D1, CDK2, CDK6, p-Akt, Gli1, Gli2, Notch1, Notch3, Hey1; ↑PARP cleavage, caspase 3 activation, Bax	↓Number of blood vessels (CD31 and vWf), COX-2, HIF-1α, VEGF, VEGFR2, IL-6, IL-8, MMP-2, MMP-9 and Snail; ↑E-cadherin	Zhao et al., 2013[6]

			level, TRAIL-R1 (DR4), TRAIL-R2 (DR5)		
♂ Syrian hamsters (8 – 10 weeks) treated with DMBA (oral cancer model)	EA	0.1, 0.2, and 0.4% (w/w) enriched diet; 14 weeks	↓Tumor incidence; ↓PI3K, Akt and PDK-1, p-Akt^{Ser473}, p-GSK-3β, and mTOR (complete suppression of the PI3K/Akt pathway); ↓p-ERK, t-ERK, p-JNK, t-JNK level; ↓HDAC-1, HDAC-4, HDAC-6 (mRNA and protein level); EA binds catalytic domains of ERK1/2, JNK1, PI3Kγ, and PH domain of Akt1 (*in silico*)	↓HIF-1α, VEGF, and VEGFR2 (mRNA and protein level)	Kowshik et al., 2014[7]
♂ Athymic BALB/c mice injected with MiaPaCa2 cells and STZ (diabetes/cancer model)	PGG	20 μg g^{-1} (gavage); 5 times per week for 8 weeks	↓Living cell area in tumor sections; ↑necrotic area; ↓p-IR, t-Akt, p-Akt, p-MEK, and p-ERK; No effect on tumor weight or GLUT-1	↓HIF-1α, VEGF, HK-II AND PFK-1; ↓p-IGF1R, t-IFG1R	Hu et al., 2020[8]
			**Flavan-3-ols/proanthocyanidins**		
NOD/SCID mice (6 – 8 weeks) injected with Namalwa, RAP1-EIO, or HS-Sultan cells (non-Hodgkin's lymphoma models)	Green Tea	1.25% (w/v; containing 708 mg mL^{-1} EGCG); 3 days per week up to 60 days	↓Tumor growth and tumor volume; ↑survival rate; ↑apoptosis of cancer and endothelial cells	No significant changes in microvessel density; ↑mouse CD31^{+} CD34^{+} and 7AAD^{+} cells	Bertolini et al., 2000[9]

♂ TRAMP transgenic mice (8 weeks) (prostate cancer model)	Green tea (poly)phenols[a]	0.1% (w/v) in drinking water; 3 days per week for 24 weeks	↓PI3k, p-Akt and p-ERK/t-ERK (prostate tissue); ↓uPA level and gelatinolytic activity	↓IGF-I and ↑ IGFBP-3 level; ↓VEGF, MMP2, MMP9, TIMP-1, and TIMP-2 level (prostate tissue and serum); ↓MMP-2/TIMP-2 and MMP-9/TIMP-1 ratio	Adhami et al., 2004[10]
♂ C57/BL6N mice injected with matrigel containing Kaposi´s sarcoma conditioned medium (cancer-related angiogenesis model)<br><br>♂ CD-1/BR nude mice (7 weeks) injected with KS-IMM cells (Kaposi's sarcoma model)	Green Tea or EGCG	<u>Green tea (p.o.):</u> 12.4 g L^{-1}; every other day for 7 and 33 days<br><br><u>EGCG (p.o.):</u> 0.05% (w/v) in drinking water; every other day for 7 and 33 days	↓Tumor size	↓Hemoglobin content in matrigel; ↓Vessel number (tumor tissue)	Fassina et al., 2004[11]
♀ A/J mice (6 weeks) treated with NNK (lung tumor model)	Green Tea	0.1, 0.2, 0.4, or 0.6% (w/v) in drinking water; 16 weeks	↓ Tumor multiplicity (only at 0.6%); ↑apoptosis (only at dose 0.6%)	↓Microvessel density (vWf and CD31) and VEGF level (at dose 0.6%)	Liao et al., 2004[12]
♀ Athymic nude mice NCr-nu/nu (6 weeks) MDA-MB-231 xenograft (breast cancer model)	Nutrient mixture (containing standardized Green tea extract)[b]	0.5% (w/w) enriched diet; 4 weeks	↓Tumor growth and proliferation (Ki-67)	↓MMP-9 and VEGF level	Roomi et al., 2005[13]
♀ Athymic nude mice NCr-nu/nu (6 weeks) HCT-116 xenograft (colon cancer model)	Nutrient mixture (containing standardized Green tea extract)[b]	0.5% (w/w) enriched diet; 4 weeks	↓Tumor growth; no effect on cell proliferation (Ki-67); ↑fibronectin	↓MMP-9 and VEGF level	Roomi et al., 2005[14]

♀ Athymic nude mice NCr-nu/nu (6 weeks) PC-3 xenograft (prostate cancer model)	Nutrient mixture (containing standardized Green tea extract)[b]	0.5% (w/w) enriched diet; 4 weeks	↓Tumor growth and cell proliferation (Ki-67); ↑fibronectin	↓MMP-9 and VEGF level	Roomi et al., 2005[15]
♀ SKH-1 hairless mice (6 – 7 weeks) exposed to UVB radiation (skin carcinogenesis model)	Polyphenon-E[c]	2 g L^{-1} in water; 24 weeks	↓Tumor incidence, size and multiplicity; ↑apoptosis and cleaved-caspase 3; ↓proliferation (PCNA); ↑$CD8^+$ cells recruitment	↓MMP2 and MMP9 level and ↑TIMP-1 level; ↓CD31 level; ↓ VEGF protein expression	Mantena et al., 2005[16]
♀ Athymic nude mice NCr-nu/nu (6 weeks) HT-1080 xenograft (fibrosarcoma model)	Nutrient mixture (containing standardized Green tea extract)[b]	0.5% (w/w) enriched diet; 4 weeks	↓Tumor growth	↓MMP-9 and VEGF level	Roomi et al., 2006[17]
♀ Athymic nude mice NCr-nu/nu (6 weeks) A2058 xenograft (melanoma model)	Nutrient mixture (containing standardized Green tea extract)[d]	0.5% (w/w) enriched diet; 4 weeks	↓Tumor growth and cell proliferation (Ki-67)	↓MMP-9 and VEGF level	Roomi et al., 2006[18]
♀ Athymic nude mice NCr-nu/nu (6 weeks) MNNG-HOS xenograft (osteosarcoma model)	Nutrient mixture (containing standardized Green tea extract)[d]	0.5% (w/w) enriched diet; 4 weeks	↓Tumor growth and cell proliferation (Ki-67); no effect on fibronectin level; ↑ascorbic acid and alkaline phosphatase (serum); ↓total protein and globulin, whereas ↑albumin; no significant effect on AST and ALT	↓MMP-9 and VEGF level	Roomi et al., 2006[19]
♀ Athymic (*nu+/nu+*) mice(4 – 6 weeks) HEY xenograft (ovarian cancer model)	Green tea	12.4 g L^{-1} (p.o.);40 and 60 days	↓Tumor growth (after 60 days); ↓ET1 and ET_AR level (mRNA and protein); ↓Proliferation(Ki-67)	↓Microvessel density (CD31) and EGF (mRNA and protein)	Spinella et al., 2006[20]

♀ Nude mice (6 weeks) ovariectomized and MCF-7 xenograft (breast cancer model)	GTE (alone or together with TAM)	2.5 g L^{-1} in water; 64 days	↓ERα level; ↓tumor volume; ↑necrosis, and apoptosis (GTE + TAM was the most effective treatment)	↓Vessel density (vWf) (only GTE + TAM was significant)	Sartippour et al., 2006[21]
♀ Sprague Dawley rats (6 – 10 weeks) treated with DMBA	Polyphenon-B	0.05% (w/w); 12 weeks	↓Tumor incidence and pathological changes; ↓tumor size and PCNA level; ↓Cyt P450 and Cyt b_5; ↑GST, GGT and DTD; ↓TBARS, CD, LOOH and protein carbonyl; ↑SOD, CAT, GPx and GSH; ↓Bcl-2, Bax and Bcl-2/Bax ratio; ↑caspase 3 and PARP	↓VEGF level	Kumaraguruparan et al., 2007[22]
♀ C3H mice (6 – 8 weeks) MCa4 xenograft and irradiated (breast carcinoma model)	EGCG	658 μg g^{-1} enriched diet; 30 days	No effect on body weight; ↑EGCG concentration in plasma and tumor tissue; no radiomodifying effect or radioprotection	No effect on microvascular density	Lawenda et al., 2007[23]
♂ Syrian hamsters (6 – 10 weeks) treated with DMBA (oral carcinoma model)	Polyphenon B[e] and BTF-35[f]	0.05% (w/w) of each product; 18 weeks	↓Tumor incidence and burden; ↓hyperplasia and dysplasia; accumulation of p21 in the nucleus; ↓GST-P, NF-κB and cytokeratins; ↓Bcl-2 and ↑Bax, Cyt C, PARP, caspases 9 and 3 (protein); ↓*Ccnd1*, *Gst-p*, *Bcl-2* and ↑*Bax* (mRNA)	↓VEGF (mRNA and protein)	Letchoumy et al., 2007[24]

♂ Wistar rats (6 – 8 weeks) treated with MNNG (gastric cancer model)	Polyphenon-B	0.05% (w/w) enriched diet; 26 weeks	↑Body weight; ↓tumor incidence, multiplicity, and burden; ↓keratosis, hyperplasia, and dysplasia; ↓proliferation (PCNA), GST-π, 4-HNE, Bcl-2 and cytokeratins, whereas ↑caspase 3, Cyt C, Bax, TBARS, LOOH, CD, and protein carbonyl; ↑SOD, Mn-SOD, Cu-Zn SOD, CAT, Se-dependent-GPx, GST, Se-independent-GPx and GSH/GSSG ratio	↓VEGF level	Murugan et al., 2007[25]
♀ C3(1)/SV40 transgenic mice (breast cancer model)	Polyphenon E[g]	0.1, 0.3 and 0.5% (w/v) in drinking water; 8, 12, 15 and 20 weeks	↓Neoplasia and tumor volume and burden; ↓tumor invasiveness and ↑tumor latency; ↓proliferation (Ki-67)	↓VEGF level and microvascular density and area (CD31)	Leong et al., 2008[26]
♂ Syrian hamsters (6 – 10 weeks) treated with DMBA (oral carcinoma model)	Polyphenon B[e] (alone or together with bLF)	Enriched diet with 0.05% polyphenon B (w/w) alone or in combination with 0.2% bLF (w/w); 14 weeks	↓Tumor incidence, multiplicity and burden; ↓CYP1A1, CYP1B1 and 8-OH-dG; ↓proliferation (PCNA); ↓ *Gst-p* and *Ccnd1* (mRNA)	↓*Vegf*, *Vegfr1*, *Mmp-9* and *Timp-2* (mRNA)	Letchoumy et al., 2008[27]
Athymic nude mice BALC/c nu/nu (4-6 weeks) AsPC-1 xenograft (pancreas cancer model)	EGCG	60, 80, 100 mg kg^{-1} day^{-1} (p.o.) administered	↓Tumor growth, cell proliferation; ↑apoptosis; ↓ERK and ↑JNK and p38 activation;	↓Microvessel density (CD31) and VEGFR2 and VEGF level; ↓MMP-2,	Shankar et al., 2008[28]

		every day, 5 days a week		MMP-7, MMP-9 and MMP-12	
APC$^{Min/+}$ mice (cancer intestinal model)	EGCG or epicatechin gallate	0.01% (w/v) in drinking water; 2 months	↓Polyps number and tumor load (only EGCG)	↓bFGF and vWf level (only EGCG)	Sukhthankar et al., 2008[29]
♀ Combined immunodeficient mice (6 – 8 weeks) MDA-MB-231 xenograft (breast cancer model)	Grape seed extract (at least 85% w/w procyanidins)	50 mg kg^{-1} (gavage); 3 weeks	↓Tumor growth and MAPK phosphorylation	↓Vessel density	Wen et al., 2008[30]
♀ Athymic nude mice (6 – 7 weeks) A549 and H1299 xenograft (lung cancer model)	Grape seed procyanidins[h]	0.1, 0.2 and 0.5% w/w) enriched diet; 29 and 68 days	No effect on body weight; ↓tumor growth (significant at 0.2 and 0.5%) and proliferation (PCNA); ↑apoptosis	↑IGFBP3 and ↓CD31 level (at 0.5%)	Akhtar et al., 2009[31]
♂ Sprague Dawley rats (6 – 8 weeks) treated with dimethylaminoazobenzene (hepatic cancer model)	Polyphenon B	0.05% (w/w) enriched diet; 24 weeks	↓Hepatomas incidence; ↓HDAC-1 and ↑RECK (protein)	↓MMP-2 and MMP-9 and ↑TIMP-2 (mRNA and protein); ↓*Vegfr1* (mRNA); ↓HIF-1α and VEGF (protein)	Murugan et al., 2009[32]
♂ BALB/c nude mice (5 weeks) HuH7 xenograft	EGCG	0.01 and 0.1% (w/v) in drinking water; 5 weeks	No effect on body weight; ↓Tumor growth, t-Akt, p-Akt, p-ERK, Bcl-x_L, and Bcl-2	↓VEGFR2 level, p-VEGFR2 (protein), and *Vegfa* (mRNA)	Shirakami et al., 2009[33]
Copenhagen rats inoculated with AT6.1 metastatic prostate tumors	Green tea capsules (each one contains 95 mg EGCG)	Three capsules (285 mg EGCG) were diluted in ~300 mL drinking water; 1 – 2 weeks	No effect on tumor growth	No effect on angiogenesis	Fan et al., 2010[34]

♀ C3H/He mice (6 weeks) treated with BBN (bladder cancer model)	Green tea (poly)phenols	0.5% (w/v) in drinking water; 14 and 24 weeks	↓Tumor volume	↓Microvessel density (CD34 and vWf)	Sagara et al., 2010[35]
BALB/c nude mice SW837 xenograft (intestinal cancer model)	EGCG	0.01 and 0.1% (w/v) in drinking water; 35 days	↓Tumor growth (dose-dependent), p-ERK and p-Akt; no effect on t-ERK and t-Akt	↓VEGFR2 and p-VEGFR2; ↓*Vegfa* (mRNA; only at 0.1%)	Shimizu et al., 2010[36]
♂ SCID mice (5 weeks) LAPC4 xenograft (prostate cancer model)	Green Tea[i]	Green tea brewed in drinking water; daily for 13 weeks	No effect on body weight; ↓Tumor volume and tumor size (significantly correlated with green tea (poly)phenols detected in tumor tissue); detection of free forms (EGCG, epicatechin and epigallocatechin) and methylated metabolites (4'-methyl-EGCG and 4'-methyl-epigallocatechin) in samples treated with β-glucuronidase and sulfatase; ↓5-cytosine DNMT1 (gene and protein level); ↓oxidative DNA and protein damage 8-oxo-dG and protein carbonyl); no effect on macrophage infiltration	↓Vessel density (CD31 staining); ↓HIF-1α and VEGF level (protein)	Henning et al., 2012[37]

♂ Wistar albino rats (4 weeks) treated with DMH (colon cancer model)	Green tea	1% (w/v) in drinking water; 32 weeks	↓Tumor incidence and ↑survival rate; ↓*Nfκb*, *Tnfa*, *Ptgs2* and *Nos2* (mRNA); ↓NF-κB, TNF-α and CRP level (protein); protection of colonic architecture	↓*Vegfc* and *Mmp-7* (mRNA)	Sadik et al., 2013[38]
♂ BALB/c nude (nu/nu) mice A549 xenograft (lung cancer model)	EGCG	0.025 and 0.05% (w/v); 24 days	↓Tumor growth (dose-dependent)	↓Microvessel density (CD34; not significant effects)	Sakamoto et al., 2013[39]
♀ C57BL/6 mice (7 weeks) E0771 xenograft (breast cancer model)	EGCG	50–100 mg kg^{-1} day^{-1} in drinking water; 4 weeks	↓Tumor growth	↓Microvessel density (CD31) and VEGF level (plasma and tumor)	Gu et al., 2013[40]
BALB/c nu/nu mice (4 – 6 weeks) PANC-1 xenograft (pancreas cancer model)	ECGC	60, 80, and 100 mg kg^{-1} (gavage); 5 days a week for 28 days	No effect on body weight; ↓pancreas weight (dose-dependent); ↑apoptosis and ↓proliferation (dose-dependent); ↑Bim isoforms and cleaved PARP and ↓pro-caspase 3 level; ↓ERK, PI3K (Tyr^{458}), p-AKT (Ser^{473}) and p-FOXO3a (Ser^{256}); ↑PTEN; no significant effect on t-ERK, t-Akt and t-FOXO3a; ↑FOXO-DNA interaction (dose-dependent); ↓*Cdkn1b*, *Cxcl8* and *Il-6* (mRNA);	↓Microvessel density (CD31), HIF-1α, VEGF and VEGFR2 level (dose-dependent); ↑E-cadherin and ↓*Cdh1*, *Cdh2*, *Zeb-1*, *Mmp-2* and *Mmp-7* (mRNA)	Shankar et al., 2013[41]

			↓NRP2 and ↑ SEMAF3F level (protein)		
SPF healthy Kunming mice (4 – 6 weeks) H22 xenograft (hepatocarcinoma model)	Grape proanthocyanidins	50 and 200 mg kg^{-1} day^{-1} (gavage); 10 days		↓Microvessel density (CD31), VEGF (mRNA and protein)	Feng et al., 2014[42]
♀ Athymic nude mice (5 – 6 weeks) HeLa xenograft (osteosarcoma model)	Nutrient mixture[a]	0.5% (w/w) enriched diet; 4 weeks	↓Tumor growth; ↑areas with Ki-67-negative cells; ↑apoptosis and ↓Bcl-2 level; ↓COX-2, GST-π and iNOS level	↓Central accumulation of MMP-2; ↓MMP-9 and VEGF staining	Roomi et al., 2015[43]
♀ C3H/He mice (6 weeks) treated with BBN (bladder cancer model)	Green tea (poly)phenols	0.5% (w/v) in drinking water; 14 and 24 weeks	No effect on cancer frequency; attenuation of tumor volume and proliferation increase; attenuation of HuR, proliferation, HO-1, and COX-2 increase	Attenuation of microvessel density and ↑VEGF increase	Matsuo et al., 2017[44]
♀ CB17/SCID mice (6 – 8 weeks) subcutaneously injected with fresh human colorectal carcinoma fragments obtained intraoperatively from patients	EGCG (alone or together with Curc)	50 mg k^{-1} day^{-1} (p.o.) every other day; 4 weeks	↓Tumor weight and volume; ↓p-JAK, p-STAT3 and IL-8 (highest effect observed in combination)	↓Microvessel density (CD31); ↓Hemoglobin (highest effect observed in combination)	Jin et al., 2017[45]
♂ C57 mice (6 weeks) treated with CCl_4 (hepatocarcinoma model)	Procyanidin B2	50, 100, and 150 mg kg^{-1} (gavage)	No toxic effect on liver function or pathology; ↓ALT, AST and hydroxyproline (dose-dependent); ↓Col1, SMO, GLI1 and α-SMA	↓VEGF-A, HIF-1α, and CD31	Feng et al., 2019[46]

♂ Athymic BALB/c mice injected with STZ and MiaPaCa2 cells (diabetes/cancer model)	EGCG	50 µg g^{-1} (gavage); 5 times per week for 8 weeks	↓Total section and living cell area in tumor sections; ↓Tumor weight; ↓p-IR, t-IR, t-Akt, p-Akt, p-MEK, t-MEK, t-ERK and p-ERK; No effect on GLUT-1	↓HIF-1α, VEGF, HK-II AND PFK-1; ↓p-IGF1R, t-IFG1R	Hu et al., 2020[8]
♀ Athymic nude mice (nu/nu) RI95-2 and AN3 xenograft (endometrial cancer model)	EGCG	50 mg kg^{-1} day^{-1} (gavage); 5 weeks	No effect on body weight; no effect on tumor growth; ↑ALT concentration (in RL95-2 model)	↓Microvessel density (CD34)	Man et al., 2020[47]
♂ Golden Syrian hamster (6 – 8 weeks) treated with DMBA (oral cancer model)	Polymeric black tea (poly)phenol extract	1.5, 3, 5, and 10% (w/v) in drinking water; 16 weeks	↓Tumor multiplicity, volume and burden; ↓number and area of microscopic lesions; no cytotoxic effects in buccal tissue; ↓8-OH-dG, cyclin-D1, COX-2, PCNA, Bcl-2 and ↑Bax; ↓p-Akt, t-Akt and mTOR level	↓EGFR, HIF-1α and VEGF	Nimbalkar et al., 2022[48]
♂ Golden Syrian hamster (5 weeks) treated with DMBA (oral cancer model)	Green tea (alone or together with Curc)	6 mg tea solid mL^{-1} in drinking water (lavage); 18 weeks	Improvement of histological lesions; ↓proliferation (CD34); ↑apoptosis (highest effect in combination with Curc)	↓Microvessel density (CD34)	Saleh et al., 2023[49]
			**Flavanones**		
Hsd:athymic nude nu/nu mice (11 weeks) injected with 786-O (*VHL* mutant)	2-HF	0.0025, 0.005, and 0.01% (w/w) equivalent to 25,	↓Ki-67, proliferation marker, level (only in 786-O xenograft);	↓CD31 level (only in 786-O xenograft); ↑E-cadherin (only in 786-O xenograft)	Nagaprashantha et al., 2011[50]

and Caki-2 (*VHL*-wild-type) cells		50, and 100 mg kg^{-1} (p.o.) every other day	↑tumor suppression marker level (only in 786-O xenograft)		
♂ Albino Wistar rats (4 weeks) injected with DMH (colon cancer model)	Hesperetin	20 mg kg^{-1} enriched diet; up to 32 weeks	Protection against colonic dysplasia; ↓PCNA-positive cells number, Bcl2/Bax ratio and COX-2 (protein and mRNA) level	↓VEGF, bFGF and EGF level	Nalini et al., 2012[51]
♀ Swiss albino mice (6 – 8 weeks) subcutaneously injected with EAC cells (cancer model)	Nar (alone or together with Curc)	Nar: 50 mg kg^{-1}; Curc: 50 mg kg^{-1}; Nar + Curc: 20 mg kg^{-1} + 20 mg kg^{-1}; oral administration (alone or in combination); for 13 days	↓EAC number and ascetic fluid accumulation; Attenuation of the cellular architecture of hepatic lobules alteration (Nar + Curc exerted the highest protection)	↓Blood vessels formation in the peritoneal cavity (Nar + Curc exerted the highest inhibition); ↓HIF-1α, VEGF, Hsp90 and Akt phosphorylation level	Anand et al., 2012[52]
BALB/c nude mice (5 weeks) subcutaneously injected with HeLa cells (cancer model)	Liquiritigenin	10, 20 and 40 mg kg^{-1}; 4 weeks	↓Tumor weight and volume and PCNA-positive cells; No effect on TSP-1	↓Microvascular density (lower CD31 and αSMA positive cells) and VEGF (serum and protein) level	Liu et al., 2012[53]
C57BL/6J-$APC^{min/+}$, SW-480 xenograft BALB/c nude mice and CT26 xenografts BALB/c mice (colorectal cancer model)	Neohesperidin	50 and 100 mg kg^{-1}; 87 days (3 days alone + 12 weeks along with a high-fat diet)	↓Tumor number; ↑apoptosis of cancer cells; No effect on cell proliferation (also tested in vitro using HCT-166 and SW-480) or Wnt/β-catenin pathway	↓CD31 marker	Gong et al., 2019[54]
♀ 123 albino rats (4 – 6 weeks); MCF-7 xenograft model (breast cancer)	Hes alone or in combination with	Hes: 200 mg kg^{-1}; Hes + piperine (ratio 1:2 w/v);	↑*Bax* and *caspase 3* and ↓*Bcl2* gene expression (Hes alone or in	↓*Vegf* gene expression (Hes alone or in combination) in mammary	Khamis et al., 2024[55]

	piperine, bee venom, and/or TAM	Hes + piperine + bee venom (ratio 2:1:1 w/v); Hes + piperine + bee venom + TAM (ratio 4:1:1:1 w/v); One daily oral administration for 4 weeks	combination) in mammary glands compared to the untreated breast cancer group; Cell cycle arrest in G0/G1 phase (by Hes alone) and G2/M phase (Hes in combination with the other compounds)	glands compared to the untreated breast cancer group	
			**Lignans/Flavolignans/Flavonolignans**		
♀ Athymic nude mice (3 – 4 weeks), MDA-MB-435 xenograft (breast cancer model)	Ground FS	10% (w/w) ground FS in the basal diet; 6 weeks	↓Tumor growth and metastasis to distant organs	↓Extracellular VEGF	Dabrosin et al., 2002[56]
♀ Athymic nude mice (4 weeks), A2780 xenograft (ovarian cancer model)	Silybin-phosphatidylcholine complex (IdB 1016) (alone or together with cisplatin)	450 mg kg^{-1}; 20 days	Potentiated the antitumor effect of cisplatin	↓FGF/heparin-stimulated angiogenesis (Hb content in Matrigel implants)	Giacomelli et al., 2002[57]
♀ Athymic nude mice (4 weeks) A2780 xenograft (ovarian cancer model)	Silybin-phosphatidylcholine complex (IdB 1016)	450 mg kg^{-1} day^{-1}; 20 days	↓Tumor weight; ↑silybin bioavailability in plasma and tumor tissue	↓*Vegfr3* and ↑*Ang2* (mRNA) level	Gallo et al., 2003[58]
♂ Athymic nude mice, DU145 xenograft (prostate cancer model)	Silibinin	0.05% or 0.1% (w/w) enriched diet; 67 days	↓Tumor volume and weight; ↓proliferation (PCNA and Ki-67); ↑apoptosis (caspase 3 activation)	↓Microvessel density (CD31); ↓VEGF and ↑IGFBP-3 level	Singh et al., 2003[59]
♂ AJ mice (6 weeks) intraperitoneally injected	Silibinin	0.033, 0.1, 0.33 or 1.0% (w/w)	↓lung tumor multiplicities and growth; ↓PCNA-positive cells and	↓Tumor microvessel density, VEGF	Singh et al., 2006[60]

with urethane (lung cancer)		enriched diet; 18 and 27 weeks	iNOS and COX-2 level; No effect on body weight or diet consumption; No significant effect on apoptosis induction		
♀ BALB/c athymic nude mice (6 – 8 weeks) MCF-7 xenograft (breast cancer model)	Ground FS	10% (w/w) enriched diet; 5 weeks	↓tumor growth; no effect on cell proliferation (Ki-67)	↓Tumor angiogenesis (vWf) and VEGF	Bergman et al., 2007[61]
♀ SKH-1 hairless mice (5 weeks) exposed to UVB-induced skin carcinogenesis	Silibinin	1% (w/w) enriched diet; 25 weeks	No effect on diet consumption or body weight; delayed tumor appearance; ↓tumor multiplicity and volume; ↓cell proliferation (PCNA) and ↑apoptosis; ↓iNOS, COX-2, and STAT3/NF-κB pathway activation	↓VEGF and HIF-1α level; ↓PECAM-1-positive cells	Gu et al., 2007[62]
♂ Athymic nude mice (6 weeks) PC-3 xenograft (prostate cancer model)	Silibinin	0.5% (w/w) enriched diet; 67 days	↓Tumor volume and weight; ↓cell proliferation (PCNA) and ↑apoptosis; ↑p-ERK/t-ERK, Cip1/p21, Kip/p27 and ↓Bcl-2	↓VEGF, IGFBP3 and survivin level; ↓microvessel density (CD31)	Singh et al., 2007[63]
♂ Athymic nude mice (6 weeks) DU145 xenograft (prostate cancer model)	Isosilibinin, silymarin and silibinin	200 mg kg^{-1} (each compound); 5 days per week for 53 days	↓Tumor volume; ↑apoptosis and ↓cell proliferation (PCNA); dissimilar modulation of cell cycle markers (cyclin	↓Microvessel density (CD31-positive cells) and VEGF level in tumor tissue	Deep et al., 2008[64]

			A, cyclin D1, cyclin D3, cyclin E, Cdk2, Cdk4, Cdk6		
♂ Athymic nude mice (6 weeks), RT4 xenograft (bladder cancer model)	Silibinin	100 and 200 mg kg^{-1} day^{-1}; 12 weeks	↓ reduction in tumor volume, tumor weight, and cell proliferation (PCNA)<br>↑ apoptosis; ↑p53 and cleaved caspase 3 level	↓Microvessel density (CD31) and survivin level;	Singh et al., 2008c[65]
♀ Sprague-Dawley DMBA-induced (7 weeks);<br>♀ BALB/c athymic nude mice (4 – 5 weeks), ovariectomized, MCF-7 xenograft (breast cancer model)	Lariciresinol	Sprague Dawley: 3 or 15 mg kg^{-1}; 9 weeks<br><br>BALB/c: 20 or 100 mg kg^{-1}; 5 weeks	↓Tumor growth; Enterodiol and enterolactone detected in serum; ↑ERα, ERβ, and PR level (in combination with estradiol)	↓Tumor vessel number and density (CD34)	Saarinen et al., 2008[66]
♂ TRAMP mice (20 weeks) (prostate cancer model)	Silybin-phytosome	0.5% and 1% (w/w) enriched diet; 11 weeks	↓tumor growth; ↓progression from prostate neoplasia to adenocarcinoma; ↓incidence of seminal vesicle invasion and distant metastasis; ↑apoptosis; ↓Epithelial-Mesenchymal Transition	↓Microvessel density (CD31), VEGF, VEGFR-2, MMP-2, MMP-3, Snail-1, vimentin, and bFGF level; ↑E-cadherin level	Singh et al., 2008a[67]
♂ Athymic BALB/c nude mice (6 weeks) HT29 xenograft (colorectal cancer model)	Silibinin	200 mg kg^{-1} day^{-1}; 32 days	No effect on body weight or diet consumption; ↓tumor volume and tumor weight<br>↓cell proliferation (PCNA and cyclin-D1) and ↑apoptosis;	↓microvessel density (CD31), VEGF and HIF-1α level	Singh et al., 2008b[68]

			↓p-ERK/t-ERK and p-Akt/t-Akt, iNOS, NOS3, COX-1, COX-2; no effect on NOS1		
♂TRAMP mice (4 – 30 weeks) (prostate cancer model)	Silibinin	1% (w/w) enriched diet; 8-15 weeks, depending on the stage of the tumor	Stage-specific inhibition of tumor growth; ↓Cdk2, Cdk4, Cdk6, Cdc2, cyclin A, cyclin B1, cyclin E; dissimilar effects on p21 and p27; ↓uPAR level; ↓cell proliferation (PCNA) and metastasis	↓Microvessel density (CD-31), VEGF, VEGFR-2, HIF-1α, MMP-2, MMP-3, MMP-9, fibronectin, snail-1, and iNOS level in tumor tissue; ↑TIMP-2 and E-cadherin level; dissimilar effects on VEGFR1	Raina et al., 2008[69]
♂Wistar albino rats treated with NDEA (hepatocellular carcinoma model)	Silymarin	1000 ppm enriched diet; 16 weeks	↓Mast cell density; ↓tumor invasion and progression	↓MMP-2 and MMP-9 level	Ramakrishnan et al., 2009[70]
♂A/J mice (4 – 6 weeks), treated with urethane (lung cancer model)	Silibinin	742 mg kg^{-1}; 5 days per week for 10 weeks	↓Tumor number and burden; ↓IL-13, TNF-α level; ↓macrophages infiltration; ↓IL-1α, IL-6, IL-9, IL-16, INF-γ (antibody array); ↓p-p65NF-κB and p-STAT3	↓Microvessel area (CD31) and nestin level; ↑TIMP-1, TIMP-2, Tie-2 and ANG-2; no effect on VEGF or p-Tie2^{Tyr992}/Tie2; ↓p-HIF-1α	Tyagi et al., 2009[71]
♂Athymic nude mice (6 weeks) SW480 xenograft (colon carcinoma model)	Silibinin	200 mg kg^{-1}; 5 days/week for 28 days	↓Tumor volume and weight; ↓proliferation (PCNA) and ↑apoptosis; ↓β-catenin, p-GSK-3β, cyclin-D1 and c-Myc	↓Microvessel density (CD31), survivin, VEGF and iNOS level	Velmurugan et al., 2010[72]

♀ BALB/c athymic nu/nu mice (6 – 7 weeks), ovariectomized, implanted with estrogen pellets and MCF-7 xenograft (breast cancer model)	ENL (alone or combined with GEN)	100 mg kg^{-1}; up to 3 weeks	No effect on food intake; ↓Tumor growth (alone or combined with GEN)	Effects exerted by ENL alone or in combination with GEN: ↓Microvessel area (vWf); ↓tumor- and stroma-derived VEGF; ↑tumor-derived PIGF; ↓endothelial cell infiltration in subcutaneously implanted matrigel	Saarinen et al., 2010[73]
♀ BALB/c athymic nude mice (5 – 6 weeks), ovariectomized, MCF-7 xenograft (breast cancer model)	FS oil or SDG	FS oil (38.5 g kg^{-1} diet) or SDG (1 g kg^{-1} diet); 8 weeks	↓palpable tumor area and regression rate (FS oil); ↓ cell proliferation and increased apoptosis (FS oil and SDG); ↓Bcl2, HER2, pHER2, and pMAPK protein level	↓IGF-1R and ↓EGFR (SDG)	Saggar et al., 2010[74]
♂ APCmin/+ mice (6 weeks), spontaneous intestinal tumorigenesis	Silibinin	750 mg kg^{-1}; 5 days per week for 13 weeks	↓Polyps formation in small intestine and colon; ↓cell proliferation (PCNA and cyclin D1), COX-2 and PGE_2; ↑apoptosis and CC3; ↓β-catenin and eNOS	↓HIF-1α, VEGF, and nestin-positive microvessels; modulation of cytokines and angiogenesis markers (array analysis)	Rajamanickam et al., 2010[75]
♂ A/J mice (5 weeks old), treated with AOM (colon cancer model)	Silibinin	250 and 750 mg kg^{-1}; 25 weeks pre-treatment followed by 16 weeks post-treatment	↓tumor multiplicity and size (dose-dependent); ↓cell proliferation (PCNA, cyclin D1), p21, β-catenin, p-Akt, p-GSK-3β; ↑apoptosis (cleaved	↓VEGF and IGF-1Rβ; ↑IGFBP3 level	Ravichandran et al., 2010[76]

			caspase 3, PARP) and CC3; ↓iNOS and COX-2		
♀ BALB/c athymic nude mice (6 – 7 weeks), ovariectomized, MCF-7 xenograft (breast cancer model)	Ground FS or ENL (together with estradiol or TAM)	10% (w/w) FS and 100 mg ENL per kg in the basal diet; 21 days	↓Tumor growth; ↓IL-1β level and proliferation; no effect on IL-1α; ↑IL-1Ra level	↓Microvessel area (vWf)	Lindahl et al., 2011[77]
♂ B6/129 wild-type and ♂ B6/129-Nos2^{tm1Lau} (iNOS$^{-/-}$) KO mice (5 – 6 weeks), treated with urethane (lung cancer model)	Silibinin	742 mg kg^{-1}; 5 days per week for 18 weeks	↓Tumor multiplicity and size (no effect observed in KO mice); ↓cell proliferation (PCNA); ↓iNOS expression; no significant effect on eNOS and nNOS	↓Nestin-positive microvessels; ↓VEGFR2, pSTAT3 (Ser^{727}), p65NF-κB (Ser^{276}) and iNOS level	Ramasamy et al., 2011[78]
♀ Athymic nude mice (6 weeks) HT-29 xenograft (colon cancer model)	FS oil	10% FS oil; 25 days	No effect on body weight and food intake; ↓Tumor growth; ↑tumor necrosis; no effect on proliferation or apoptosis; no effect on antioxidant capacity, CRP, adiponectin, leptin, insulin, glucose, free fatty acids; ↓MCP-1, MIP-α, pentaxin-3	No significant effect on microvessel density (CD34); ↓11 angiogenic proteins in serum (Cyr61, endoglin, fractalkine, MCP-1, MIP-1α, pentraxin-3, SDF-1, serpin F1, TIMP-1, TIMP-4, VEGF, IGFBP-1); ↑IGFBP-2 and IGFBP-3; no effect on serum IGF-1 level	Nagel et al., 2012[79]
♂ Athymic nude mice (4 – 6 weeks) DU145 xenograft (prostate cancer model)	Silybin A, Silybin B, Isosilybin A, Isosilybin B	50 and 100 mg kg^{-1}; 10 weeks	↓Tumor growth and cell proliferation; ↑apoptosis via caspase pathway	↓VEGF, VEGFR1, and VEGFR2 level; ↓angiogenesis (CD31) in tumor tissues	Deep et al., 2012[80]
♂ Athymic nude mice (6 weeks), BxPC-3 and	Silibinin	0.5% (w/w) enriched diet; 7	In BxPC-3: ↓Tumor volume and weight;	↓Angiogenesis (CD31) in both models (36% in	Nambiar et al., 2013[81]

PANC-1 xenografts (pancreatic cancer model)		weeks for BxPC-3, 11 weeks for PANC-1	↓proliferation and ↑apoptosis<br><br>In PANC-1: ↓Tumor volume and weight	BxPC-3 and 24% in PANC-1 tumors)	
♀ Athymic nude mice (4 – 5 weeks), A375 melanoma xenograft (BRAF-mutated)	Silymarin	500 mg kg^{-1}; twice a week for 32 days	No effect on body weight; ↓Tumor growth and proliferation (PCNA, cyclin-D1, cyclin-D2, Cdk2, Cdk4, and Cdk6); ↑Cip1/p21 and Kip1/p27; ↑apoptosis; ↓Bcl-2 and Bcl-xl; ↑Bax and cleaved caspase 3	↓Angiogenesis (CD31), MMP-2, MMP-9 and VEGF in tumor tissues	Vaid et al., 2015[82]
♀ BALB/c mice (10 weeks), fumonisin B1-induced hepatotoxicity	Silymarin	100 mg kg^{-1}; 14 days	↓NF-κB activity, TNF-α, and caspase 8 levels; Improved liver antioxidant capacity and reduced liver damage	↓VEGF and FGF-2 level	Sozmen et al., 2014[83]
♀ Swiss albino mice with Ehrlich ascites carcinoma (EAC)	FS, FS oil, or FS meal	10% (w/w) enriched diet; 3 weeks	↓Tumor volume; no effect on GSH and MDA level; ↓ER, PR, proliferation (Ki-67); ↑caspase 3 level	↓IGF, VEGF, and MMP-2 level	Ezzat SM et al., 2018[84]
♂ Wistar rats, CCl_4-induced liver fibrosis	Silymarin (alone or in combination with Lisinopril)	30 mg kg^{-1}; 6 weeks	↑Mortality rate; ↓Necro-inflammation score (together with lisinopril) and fibrotic area; ↓ALT, AST and ALP; ↓TNF-α; ↓MDA, hydroxyproline and NO;	↓VEGF, α-SMA, TGF-β, MMP-2 and TIMP-1 level	Saber et al., 2018[85]

			↑GSH and SOD; ↓*Rela* expression (NF-κBp65 gene); ↓IκBα and NF-κB p-p65/p65 ratio		
♀ Laying hens (ovarian cancer model)	Whole FS, FS oil, or FS meal	15% (w/w) whole FS diet, 5% (w/w) FS oil, or 10% (w/w) FS meal; 1 year	↓Tumor incidence and severity; ↑apoptosis and ↓tumor progression (FS oil and FS meal)	↓Angiogenesis (CD31), VEGF, VEGFR2 and α-SMA level	Pal et al., 2019[86]
♂ Wistar albino rats (8 weeks) treated with CCl_4 to induce liver fibrosis	Silymarin	100 mg kg^{-1}; 8 weeks	↓Liver fibrosis; ↓serum ALT, AST, GGT and ALP levels (not significant); ↑GSH and TAC (not significant); ↓MDA and NO; ↓IL-6 and TNF-α; ↓*Caspase 3* (mRNA)	↓VEGF and SDF (not significant) level; ↓PDGF level (not significant); ↓*α-Sma* (mRNA)	Zein et al., 2022[87]
♀ Swiss albino mice (2 months), Ehrlich solid tumor	Silymarin	200 mg kg^{-1} (p.o.); 15 days	↓Tumor growth; Cell cycle arrest at Sub-G1 and G2/M phases; ↓cell Proliferation (Ki-67 and PCNA); ↑p53, Bax, caspases 3 and 9, and CD95 (Fas receptor) levels; ↓NF-κB, Bcl-2 level and neoplastic cells	↓VEGF and ↑E-cadherin levels in tumor tissues	Amer et al., 2022[88]
♂ Swiss albino mice, CCl_4-induced liver fibrosis	Silymarin	100 mg kg^{-1}; three times a week for 6 weeks	↓ALT, AST, TG, and TB levels (not significant) and TCh; ↓hepatic tissue alterations and inflammatory cells infiltration; moderated	↓VEGF (not significant) and CD34 tissue level	Melaibari et al., 2023[89]

			reduction of collagen fibers accumulation; ↑GSH, CAT, and ↓MDA; ↓IL-1β, TNF-α and IL-6 (not significant)		
		**Isoflavones**			
♂ SCID mice (8 weeks) LNCaP xenograft (prostate cancer model)	SPI and SPC	SPI: 20% (w/w; 415 mg ISF equivalents kg^{-1}) SPC: 0.2% (w/w; 314 mg ISF equivalents kg^{-1}) SPI + SPC: 20% + 0.2% (w/w; 756 mg ISF equivalents kg^{-1}) SPC: 1% (w/w; 1,075 mg ISF equivalents kg^{-1}) SPI + SPC: 20% + 0.2% (w/w; 2,120 mg ISF equivalents kg^{-1})	No effect on food intake or body weight; ↓tumor growth (significant in 1% SPC and the mixtures); ↑apoptosis and ↓cell proliferation (significant in SPC and the mixtures)	↓Microvessel density (vWf); ↓serum IGF-I (only measured in 20% SPI + 0.2% SPC)	Zhou et al., 1999[90]
BALB/c mice colon-26 xenograft (colon carcinoma model)	GCP	0.3 g day^{-1} (p.o.) once a day; 5 days		↓Angiogenesis	Miura et al., 2002[91]
♂ SCID-beige mice (8 weeks)	SPC (51.9% w/w; ISF: 50.8% GEN, 40.5% DAZ, and 8.7% glycitein equivalents) and SPC-	0.5% (w/w) SPC (812 mg GEN, 648 mg DAZ, 139 mg glycitein equivalents) and	No effect on food intake and body weight; ↓tumor growth and serum PSA (only significant in genistin and	↓Microvessel density (vWf); ↓tumor bFGF level; no effect on VEGF level	Zhou et al., 2002[92]

	purified genistin (90.1% GEN, 9.1% DAZ, and 0.8% glycitein aglycone equivalents)	0.14% (w/w) genistin (812 mg GEN equivalents); 2 weeks	SPC); ↓metastases (significant in SPC); ↑apoptosis; ↑tumor p53 level (only SPC); no effect on cell proliferation or p21/wal1 level in tumor tissue; ↓serum testosterone by genistin (non-significant); ↓serum DHT (only genistin); ↑testosterone/DHT ratio (only genistin); ↓serum androgen (non-significant)		
♂SCID mice (8 weeks) LNCaP xenograft (prostate cancer model)	SPC, BT, GT, SPC + BT, and SPC + GT	SPC (5g kg^{-1}), BT (15 g tea leaves per liter of water); GT (15 g tea leaves per liter of water); SPC + BT and SPC + GT (5g kg^{-1} + 15 g tea leaves per liter of water); 10 weeks	No effect on food intake or body weight; no effect on cell apoptosis; ↓tumorigenicity; ↓tumor metastasis (only mixtures); ↓tumor growth and serum PSA level (except GT); ↓proliferation; ↓serum testosterone (only GT) and DHT (only BT and SPC + GT)	↓Microvessel density (vWf)	Zhou et al., 2003[93]
♀SCDI mice (5 – 8 weeks) MCF-7 xenograft (breast cancer model)	SPC, GSI, BT, GT, SPC + BT, and SPC + GT	<u>SPC:</u> 0.1 and 0.5% <u>GSI:</u> 0.014 and 0.028% <u>BT:</u> 1.5%; (w/v)	↓Tumor growth (except BT); ↓cell proliferation;	↓Vessel density in the mixtures treatments (vWf); ↓IGF-I (only SPC + GT).	Zhou et al., 2004[94]

		GT: 1.5% (w/v) SPC + BT and SPC + GT: 0.1% + 1.5% (w/v); 10 weeks	↑apoptosis (BT, GT, and mixtures); ↓ER-α (BT and mixtures)		
♀ Severe combined immunodeficient mice (6 – 8 weeks) 253J B-V xenograft (bladder cancer model)	SPC (51.9% w/w ISF: 50.8% GEN, 40.5% DAZ, and 8.7% glycitein equivalents) and genistin	SPC: 0.5% (w/w); 2 weeks Genistin: 0.14% (w/w); 2 weeks	No effect on body weight or food intake; ↓Tumors weight; ↓lung metastasis and tumor NF-κB level (only significant in SPC); ↑apoptosis; no anti-proliferative effect	↓Angiogenesis (vWf); ↓serum IGF-I (only significant in SPC); no effect on serum IGFBP3 and bFGF	Singh et al., 2006[95]
♀ Athymic mice (5 – 6 weeks) ovariectomized, implanted with estrogen pellets and MCF-7 and MDA-MB-231 xenograft (breast cancer model)	SOYSELECT extract (SSE, Indena spa, Milan, Italy) containing 13 – 17% GEN and DAZ, and not <18% of B-group saponins	50 and 100 mg kg^{-1} day^{-1} (p.o.); 5 or 6 consecutive days per week	No effect on tumor growth or body weight; no effect on uterus weight; ↓*Cyclin D1* and *KLK6*, whereas ↑*Ps2* and *PR* (mRNA) in the absence of 17β-estradiol; ↓*Cyclin D1*, *Ps2*, *PR*, *KLK6,* and *TSP* in the presence of 17β-estradiol	↓Angiogenesis (Drabkin´s technique); ↓*TGFβ2* (mRNA)	Gallo et al., 2006[96]
♂ Syrian golden hamsters (6 weeks) treated with DMBA (oral cancer model)	GEN	10 mg kg^{-1} day^{-1} (p.o.); 18 weeks	↓Tumor incidence and growth (not significant); ↑poorly differentiated tumors	No significant effect on microvascular density in tumor tissue	Yang et al., 2006[97]
♀ Sprague-Dawley rats treated with DMBA (breast cancer model)	SP	20% (w/w) enriched diet; 172 days	↓Tumors number and incidence; absence of aggressive grade II and III tumors; ↑tumor latency; ↓proliferation (Ki-67)	↓Angiogenesis (CD31); ↓VEGF and bFGF level	Mukhopadhyay et al., 2006[98]

♀ Sprague-Dawley rats treated with DMBA (breast cancer model)	IDSP	3.3 gr rat^{-1} day^{-1} (dietary administration); 18 weeks (4 weeks before and 14 weeks after DMBA treatment)	↓Tumor growth and incidence; ↑latency; ↓HSP90 and NF-κB; ↑p53, p21, and caspase 3 level; ↑caspase 3 activity	↓VEGF level	Park et al., 2009[99]
♀ Sprague-Dawley rats ovariectomized and treated with DMBA (breast cancer model)	GEN, DAZ, and SPE	GEN or DAZ: 50 mg kg^{-1}; 20 weeks. SPE: 50 and 100 mg kg^{-1}; 20 weeks	↓Tumor weight (only after SPE diet); ↑apoptosis	↓Microvascular density (CD34); ↓VEGF level; ↑endostatin level (only DAZ and SPE); no effect on bFGF level	Kang et al., 2009[100]
♀ BALB/c athymic nu/nu mice (6 – 7 weeks) ovariectomized, implanted with estrogen pellets and MCF-7 xenograft (breast cancer model)	GEN (alone or combined with ENL)	100 mg kg^{-1}; up to 3 weeks	No effect on food intake; dissimilar effects on tumor growth in animals fed GEN (↑) or GEN + ENL (↓)	↓Microvessel area (vWf), stroma-derived VEGF and ↑tumor-derived PIGF (only in combination with ENL); distinct effect on endothelial cell infiltration in subcutaneously implanted matrigel in animals: ↑ by GEN and ↓ by GEN + ENL	Saarinen et al., 2010[73]
BALB/c nude mice (~4 weeks) SK-N-SH NB xenograft (neuroblastoma model)	GEN	2 mg (p.o.) daily; 15 days	↓Tumor growth; ↑ CHD5 level (mRNA and protein) and ↓CHD5 methylation; ↑*p53* and ↓*DNMT3b* (mRNA); no effect on *DNMT1* or *DNMT3a* (mRNA)	↓Microvessel density (vWf)	Li et al., 2012[101]

C57BL/6J mice LLC xenograft (lung cancer model)	Glyceollins isolated from soybean seeds (glyceollin I: 77.96%, II: 10.07% and III: 11.97%)	10 mg kg^{-1} (p.o.); every 24 hours for 24 days	↓Tumor growth; ↓p-ERK, p-JNK, p-P38, p-FAK in tumor tissues	↓Microvessel density (CD31-positive cells); ↓p-VEGFR2 in tumor tissue	Lee et al., 2013[102]
♀ Sprague-Dawley rats treated with PhIP (breast cancer model)	Soymilk (alone or together with 2 × 10^{11} CFU/kg *Lactobacillus casei* Shirota)	100 g kg^{-1} (total ISF: 335 mg kg^{-1} diet); 17 weeks	↓Tumor incidence and multiplicity (only significant in combination with L casi); ↓Tumor multiplicity and diameter; ↓body weight and liver relative weight; ↓ERα-positive and Ki-67-positive cells	↓Microvessel density (CD34-positive cells)	Kaga et al., 2013[103]
♀ Rats (3 months) ovariectomized (endometrial cancer)	GEN	50 mg kg^{-1} (p.o.); administered daily (1 mL) immediately after ovariectomy or 1 month later; 1 month	↑Endometrial area and thickness, epithelial thickness, number of endometrial glands	↑Number of blood vessels, VEGF-A and Ki-67; ↑*Esr1*, *Ccna1*, *Ccnd1*, *Cdkn1a*, *Cdkn2a*, *Ccne2* (mRNA); ↑*Ccna2*, *Cdnkn1b*, *Ccne1* (mRNA; no significant); no effect on *Esr2*, *Vegfa*, *Mki-67* (mRNA)	Carbonel et al., 2015[104]
C57BL/6J mice LLC xenograft (lung cancer model)	Glyceollins isolated from soybean seeds (glyceollin I: 77.96%, II: 10.07% and III: 11.97%)	10 mg kg^{-1} (p.o.) administered every 24 hours; 24 days	↓p-mTOR, p-Akt, p-CDK4 and p-CA9; no effect on HSP90	↓Microvessel density (CD31)	Lee et al., 2015[105]
BALB/c athymic mice (6 – 8 weeks) HCT-116-LU xenograft (colon cancer model)	GEN	25 and 75 mg kg^{-1} day^{-1} (p.o.); 5 days a week for 5 weeks	↓Lung and liver tumor metastasis; ↓tumor growth (no significant); no effect on behavior or	↓Microvessel density (CD34 staining) and MMP-2 and FLT-4 level in tumor tissues (dose-dependent)	Xiao et al., 2015[106]

			weight; ↓Ki-67 positive cells (no significant)		
♀ BALB/c mice MDA-MB-231 xenograft (breast cancer model)	Formononetin	100 mg kg^{-1} day^{-1} (p.o.); 25 days	↓Tumor growth; no effect in body weight and no lesions observed in vital organs; ↓ p-$STAT3^{Ser727}$ positive cells; ↓p-Akt^{Thr308}, p-$STAT3^{Ser727}$p-$PI3Kp85^{Tyr458}$	↓Microvessel density (CD31), p-$FGFR2^{Tyr463}$ positive cells; ↓p-$FGFR2^{Tyr463}$, MMP-2 and MMP-9 level (protein)	Wu et al., 2015[107]
♀ Wistar rats (3 months) ovariectomized (breast cancer model)	FSE rich in GEN and DAZ (Novasoy-ADM Natural Health & Nutrition, Decatur, IL)	150 mg kg^{-1} (p.o.); 1 month	No effect on Ki-67 level	No effect on VEGF-A protein level	Aparecida Santos et al., 2016[108]
♀ BALB/c mice 4T1 cell xenograft (breast cancer model)	SIE (alone or in combination with docetaxel)	100 mg kg^{-1} (0.01%); 1 week or until animals died	No significant effects in food intake; ↑survival rate and ↓tumor growth (no significant) in combination with docetaxel; ↓NF-κBp65 (mRNA and protein); no significant effect in Pgp	↓VEGFR2 protein level (no significant effect at the mRNA level)	Hejazi et al., 2017[109]
♂ Sprague Dawley rats (6 weeks) subcutaneously injected with MTX (chemotherapy model)	GEN	20 mg kg^{-1} (gavage) daily; 1 week alone + 5 days together with daily injections of MTX and sacrificed after 4 days	↓BM sinusoids dilatation; ↑apoptosis (cleaved caspase 3)	↑Pecam1 (mRNA) and VEGF level (mRNA and protein) in bone tissue	Hassansani et al., 2018[110]
♂ Sprague Dawley rats (4 – 6 weeks) fed MNNG in	Calycosin	40 and 80 mg kg^{-1}; 10 weeks	Amelioration of intestinal metaplasia and dysplasia;	Improvement of microvascular	Li et al., 2020[111]

drinking water (gastric carcinoma model)			protection of the gastric wall architecture; ↓NF-κB, p-NF-κB; DARPP-32, STAT3, and integrin β1	abnormalities and protection against vascular damage	
♀ Sprague Dawley rats (7 weeks) treated with DENA (hepatocarcinogenesis model)	SPI	20% (w/w) SPI-enriched diet (containing 12.81 mg daidzin, 2.73 mg DAZ, 29.42 mg genistin, and 7.47 mg GEN kg^{-1} diet); administered for 8 weeks (before mating, pregnancy, and lactation) followed by the consumption of a normal diet for 15 weeks	↑Mortality rate, tumor multiplicity and incidence, and liver damage; alteration in cholesterol metabolism; ↑serum GGT activity, AST, ALT, bilirubin and ↓total protein and albumin; ↓hepatic ABCA1/HSC70 and HDL; ↑hepatic β-catenin/HCS70 and ↓L-FABP/HCS70 (protein); ↑*Mmp9* and *Hmox1* (mRNA); ↑apoptosis via reduction of p-IKKβ/HSC70 and reduction of cleavage caspase 3/HSC70	↑Angiogenesis	Choi et al., 2020[112]
♂ SPF Sprague Dawley rats (8 weeks) injected with glacial acetic acid (gastric ulcer model)	Formononetin	25, 50 and 100 mg kg^{-1} (p.o.); once a day for 2 weeks	Protection of gastric mucosa architecture; ↓neutrophils infiltration; ↑ZO-1, and occludin; ↓TNF-α, IL-1β, IL-6 and MPO; ↓p-P65/P-65 and	↑CD34, VEGF, ET-1 and NO	Yi et al., 2022[113]

			p-IκBα/IκBα (NF-kB pathway)		
♀ BALB/cAnNRj-Foxn1^{nu} (7 weeks) ovariectomized, injected with MCF-7 and implanted with an estradiol pellet (breast cancer model)	Glyceollins soybean extracts	20 mg kg^{-1} (jelly enriched with a natural glyceollin extract); 8 weeks	↓Tumor growth; No effect on ERα; ↓Ki-67 and p-H_3S_{10}	↓CD31 and CD34 level	Ferriere et al., 2024[114]
		**Flavones**			
♂ ICR mice (5 weeks) treated with AOM (colon cancer model)	Hydroxylated PMF[j]	0.01 and 0.05% (w/w) enriched diet; 6 and 20 weeks	No effect on body or organ weight; ↓number of crypt aberrant foci and microadenoma; ↓β-catenin and ↑cyclin D1; ↓p-Akt (Ser^{476}) and p-GSK3β (^{Ser9}); ↑E-cadherin; ↓iNOS, COX-2 and ornithine decarboxylase (mRNA and protein) level; ↓NF-κB pathway activation; ↓p-STAT3 (Ser^{727} and Tyr^{705}) and Ras activation;↓PARP and caspase 3 activation and activity	↓p-EGFR (Tyr^{845}, Tyr^{992} and Tyr^{1068}) and p-ERK; ↓VEGF and MMP-9 level	Lai et al., 2011[115]
BALB/c nude mice HepG2 xenograft (hepatic cancer model)	TTF-1 and apigenin	<u>TTF-1:</u> 5, 10, 20 μmol kg^{-1} (p.o.) <u>Apigenin:</u> 10 μmol kg^{-1} (p.o.); once a day for 10 days	↓Tumor growth and tumor number; ↓COX-2 level	↓Microvessel density (CD34); ↓VEGF, VEGFR2, HIF-1α and bFGF (mRNA and protein) level	Liu et al., 2011[116]

♂ and ♀ Heterozygous C57BL/TGN TRAMP mice, Line PB Tag 8247NG (8 weeks) (prostate cancer model)	Apigenin	20 and 50 mg $mouse^{-1}$ day^{-1} (p.o.); 6 days per week for 20 weeks	↓Tumor incidence and metastases; ↓tumor differentiation; ↓PCNA tumor level; no effect on *PB-Tag* transgene expression; ↓p-Akt, p-ERK level and uPA	↓IGF-I, VEGF, MMP-2, MMP-9 and ↑IGFBP-3	Shukla et al., 2012[117]
♂ BALB/ca nude mice (4 – 5 weeks) SAS xenograft (tongue cancer model)	Scutellarin (alone or combined with ultrasound treatments)	10 mg kg^{-1} (p.o.); every 2-3 days for 18 days	↓Tumor growth; ↑apoptosis and ↓PCNA	↓Microvessel density (CD105) and lymphatic vessel density (D2-40); ↓MMP-2 and MMP-9	Li et al., 2013[118]
♂ Golden Syrian hamsters (8 – 10 weeks) treated with DMBA (oral cancer model)	Apigenin	2.5 mg kg^{-1} day^{-1} (p.o.); 16 weeks	↓Tumor growth and incidence; ↓PCNA, p53, Bcl-2 and ↑Bax; ↑caspases 3 and 9 and ↓COX-2 and c-fos level; ↓Ccnd1 and *Nfkb* (mRNA)	↓VEGF	Silvan and Manoharan 2013[119]
♂ C3H/He mice (5 weeks) subcutaneously injected with metastatic osteosarcoma LM8 cells (osteosarcoma model)	Wogonin	25 or 50 mg kg^{-1} (p.o.) twice daily for 35 days	↓Tumor weight and volume and PCNA-positive cells in the tumor; ↓MCP-1 level; ↓ LYVE-1 positive area and macrophage accumulation ($F4/80^+$ cells); ↓HIF-1α level (not significant)	↓CD31 marker	Kimura and Sumiyosi 2013[120]
♂ and ♀ Heterozygous C57BL/TNG TRAMP mice (8 weeks) (prostate cancer model)	Apigenin	20 and 50 mg $mouse^{-1}$ day^{-1}; 20 weeks	No toxicity or effect on weight observed; ↓genitourinary, dorsolateral, and ventral	↓VEGF	Shukla et al., 2015[121]

			prostate weight; ↓tumor differentiation grade; ↑non-cancerous prostate tissue; ↓metastases; ↓NF-κB/p65 and p-IKKα (Serine[176/177]); ↓IKKa kinase activity; ↑IκBα level and ↓p-IκBα; ↓PCNA, COX-2 and cyclin D1; ↓Bcl2, Bcl-xl and ↑Bax		
♂ Athymic BALB/c nu/nu mice (8 weeks) Caki xenograft (renal cancer model)	Hispidulin (alone or together with sunitinib)	Hispidulin (p.o.): 20 mg kg^{-1} day^{-1} Hispidulin + sunitinib (p.o.): 20 + 20 mg kg^{-1} day^{-1}; 4 weeks	↓Tumor growth; no effect on body weight; ↓Ki-67 level (mixture)	↓Microvessel density (CD31)	Gao et al., 2015[122]
♀ F344 rats (5 weeks)injected with AOM (colon cancer model)	Nobiletin (alone or together with atorvastatin)	Nobiletin: 0.1 and 0.05% (w/w); 40 weeks Nobiletin + atorvastatin: 0.05 + 0.02% (w/w); 40 weeks	No effect on body and organs weight; ↓tumor multiplicity (the mixture) and incidence; ↑p21 and ↓CDK2, CDK4 and cyclin D and E; ↑p53, cleaved caspases 7 and 3; ↑cleaved PARP; ↓IL-1β, IL-6, TNF-α and COX-2 level; ↓RhoA level	↓VEGF, MMP-9, p-EGFR, EGFR	Wu et al., 2017[123]
♂ Syrian hamsters treated with DMBA (oral carcinogenesis model)	Diosmin	100 mg kg^{-1} b.w.	↓Tumor incidence, body weight changes and growth rate; ↓IL-6, JAK1,	↓VEGF and HIF-1α	Rajasekar et al., 2016[124]

			STAT-3 nuclear and ↑STAT-3 cytosolic; ↓Bcl2 AND Bcl-xl; ↑BAX, caspases 3 and 9; Amelioration of abnormal expression of PCNA-positive cells and cyclin-D1; protection of buccal tissues		
♀ BALB/c nude mice (6 weeks) and ♀ NOD/SCID immunodeficient mice (6 weeks) inoculated with HS-2, U937, and U937/MDR (leukemia model)	Wogonoside	80 mg kg^{-1}; 10 and 21 days	↓Tumor growth and progression; ↑Survival rate	↓Blood vessel formation	Li et al., 2018[125]
♂ BALB/c thymic nude mice (6 weeks) A549 xenografts (lung cancer model)	Baicalein, baicalin and wogonin	<u>Baicalin:</u> 80 mg kg^{-1}; 4 weeks <u>Baicalein:</u> 40 mg kg^{-1}; 4 weeks <u>Wogonin:</u> 80 mg kg^{-1}; 4 weeks	↓Tumor volume (only significant for baicalein after 28 days) and weight; No effect on body weight	↓CD31, Id1, VEGF-A, N-Cadherin, and vimentin; ↑E-Cadherin	Zhao et al., 2019[126]
♀ BALB/c nude mice (6 weeks); MDA-MB-231 subcutaneously implanted (breast cancer)	Wogonoside	50 mg kg^{-1} (p.o.); once every other day for 15 days	↓Tumor volume and weight; ↓Gli1 nuclear translocation and expression in the nucleoplasm; ↓SMO protein expression; Strong interaction between wogonoside and SMO and weak	↓VEGF level	Huang et al., 2019[127]

			interaction with Gli1 (predicted in silico)		
♀ BALB/c nude mice (4 weeks) SiHa xenograft (cervical carcinogenesis model)	Engeletin	50 mg kg^{-1} (p.o.); 1 month	↓Tumor growth and cell density; ↓cell proliferation (Ki-67); ↓CCL2 and p-NFκB/NFκB	↓VEGF-A and MMP-9	Bai and Yin, 2020[128]
♂ BALB/c nude mice subcutaneously injected with A549 cancer cells (lung cancer model) and ICR mice (4 – 6 weeks)	Bicalein	<u>Baicalein alone (p.o.):</u> 50 mg kg^{-1} <u>Baicalein + docetaxel (p.o.):</u> 50 + 5 mg kg^{-1}, 50 + 10 mg kg^{-1}, 50 + 15 mg kg^{-1}); 15 days (p.o.)	↓Tumor weight (highest effect observed in combination with docetaxel); ↑therapeutic index (in combination); ↑apoptosis; no toxicity observed in non-tumor bearing mice	↓CD31 marker	Lu et al., 2020[129]
BALB/c nu/nu mice (5 – 6 weeks) A549 xenograft (lung cancer model)	Baicalein	10 mg kg^{-1} day^{-1} (p.o.); 3 weeks	↓Tumor growth; ↑tumor necrosis and bleeding; ↓ROCK1, ROCK2 and MLC (mRNA and protein)	↓Vasculogenic mimicry (CD34/PAS)	Zhang et al., 2020[130]
♂ TRAP rats with the genetic background of Sprague Dawley (6 weeks) and ♂ castrated nude mice PCai1 xenografts or 22Rv1 xenografts (prostate cancer model)	Luteolin	20 and 100 ppm (p.o.); 2, 6, and 8 weeks (rats, PCai1 xenograft mice, and 22Rv1 xenograft mice experiments, respectively)	<u>Rats experiment:</u> No effect on body and organs weight; ↑serum testosterone (dose-dependent); no effect on testosterone/estradiol ratio; ↓prostatic lesions progression; ↑tumor lesions and ↓incidence (at the highest dose); ↑apoptosis (dose-dependent) and ↓cell	<u>PCai1 and 22Rv1 xenograft mice experiment:</u> ↓Microvessel density (CD31)	Naiki-Ito et al., 2020[131]

			proliferation (Ki-67); ↑caspases 3 and 7; ↓p-NF-κB and cyclin D1; no effect on AR and SV40T level; ↓ROS (dose dependent); ↓Gpx2 and clusterin (mRNA and protein); ↓*HO-1* and *IL-1b* (mRNA)<br><br><u>PCai1 and 22Rsv1 xenograft mice experiment:</u><br>↓Tumor growth and ↑apoptosis; ↓Gpx2, AR-FL and AR-V7; ↑*mi8080* gene expression; synergic effect with enzalutamide		
♂ BALB/c thymic nude mice (6 weeks) A549 xenograft (lung cancer)	Baicalein	40 mg kg^{-1} day^{-1} (p.o.); 28 days	↓Nodules in lungs; ↓Id1, N-cadherin, vimentin (protein); ↓p-Src	↓VEGF-A and ↑E-cadherin	Zhao et al., 2019[132]
♀ Sprague Dawley rats (8 – 10 weeks) treated with DMBA (breast cancer model)	Tangeretin (alone or together with pomegranate)	<u>Tangeretin:</u> 50 mg kg^{-1} (p.o.); 1 month<br><u>Tangeretin + pomegranate:</u> 50 mg kg^{-1} + 5 g kg^{-1} (p.o.); 1 month	Attenuation of the effect of DMBA on body weight and growth rate; ↓tumor growth and incidence; ↓CA 15-3 level and CEA level; ↑p53 and Bax level (only the mixture); ↓Cyclin D1 (only tangeretin); no effect on β-catenin and Bcl-2;	↓VEGF serum level; no effect on serum MMP-9;	Gul et al., 2021[133]

			↓ERα, Ki-67 and ↑apoptotic index; ↓NF-κB serum level (only the mixture)		
♂ BALB/c mice (4 weeks) HepG2/C3A xenograft (liver cancer model)	Senensetin	40 mg kg^{-1} (p.o.); daily for 2 weeks	No effect on body weight; ↓tumor growth;	↓CD31 and VEGF level	Li et al., 2022[134]
BALB/c nude mice (4 – 6 weeks) FaDu cells xenograft exposed to radiotherapy (laryngeal carcinoma model)	Luteolin (effect tested with radiation treatment performed in parallel)	100 mg kg^{-1} (p.o.); 13 days	↓Tumor growth (effect attenuated when integrin β1 is overexpressed); ↓tumor Integrin β1-positive expression; ↓Ki-67 level and p-Akt/Akt ratio (effect attenuated when integrin β1 is overexpressed)	↓VEGF-A and microvessel density (CD31 level)	Li et al., 2023[135]
♀ BALB/c mice (6 – 8 weeks) 4T1 xenograft (breast cancer model)	LU-LNP, LU/SL-LPN, and LU/SL-SDPN (ratio LU/SL 1:1)	80 mg kg^{-1} day^{-1} (gavage) every other day; 4 weeks	↓Metastatic nodules (only LU/SL-SDPN significant)<br><br><u>Effects only tested with LU/SL-SDPN:</u><br>↑necrosis and inflammatory cell infiltration; ↑M1/M2 ratio and CD45$^+$ cells; ↑HMC-II$^+$/CD206$^+$ ratio; ↑IL-6 and CD86, whereas ↓IL-10 and CD206 (mRNA and protein); ↓*Stat3*,	<u>Effects only tested with LU/SL-SDPN:</u><br>↓TGF-β1, CD31 and MMP-9 level (protein); ↓*Hif1a* expression (mRNA)	Lu et al., 2023[136]

			Myc, *Slc2a1* and *Ldha* (mRNA); ↓Col1 and α-SMA		
♂ Swiss albino mice, CCl_4-induced liver fibrosis	Apigenin (alone or in combination with CCl_4)	2 and 20 mg kg^{-1}; three times a week for 6 weeks	↓ALT, AST, TG, and TB levels and TCh (not significant); ↓hepatic tissue alterations and inflammatory cells infiltration (highest protection at 20 mg kg^{-1}); moderated (at 2 mg kg^{-1}) and minimal (at 20 mg kg^{-1}) reduction of collagen fibers accumulation; ↑GSH, CAT, and ↓MDA (only significant at 20 mg kg^{-1}); ↓IL-1β and IL-6 (significant at 20 mg kg^{-1}); ↓TNF-α (significant at 2 and 20 mg kg^{-1}); no effect of apigenin alone	↓VEGF and CD34 tissue level (only significant at 20 mg kg^{-1}); no effect of apigenin alone	Melaibari et al., 2023[89]
			**Flavonol**		
♂ SCID mice (9 – 10 weeks) CWR22 xenograft (prostate cancer model)	Quer (alone or combined with TAM)	<u>Quer:</u> 50, 100 and 200 mg kg^{-1} (p.o.); 4 weeks<br><u>Quer + TAM:</u> 200 mg kg^{-1} (p.o.) + 10 mg kg^{-1} (i.p.); 4 weeks	↓Tumor growth and incidence (significant at the highest concentration and combined with TAM); no effect on body weight; ↓Ki-67-positive cells; no significant effect on cdc-2	↓Microvascular density (only combined with TAM); ↓$Vegf^{121}$ and $Vegf^{165}$ (combined with TAM) and ↑$Vegf^{121}$ and $Vegf^{165}$ (Quer alone)	Ma et al., 2004[137]

			phosphorylation; ↓Cyclin B1		
♀ BDF1 mice B16F10 xenograft (skin cancer model)	Quer or Quer-SBE7βCD complex	Quer: 25 and 50 mg kg^{-1} (p.o.); 3 weeks<br>Quer-SBE7βCD complex: equivalent to 25 and 50 mg kg^{-1} (p.o.); 3 weeks	↓Tumor growth	↓Microvessel density (highest effect exerted by the complex treatment)	Kale et al., 2006[138]
♂ Syrian hamsters (8 – 10 weeks) treated with DMBA (buccal cancer model)	Quer	25 mg kg^{-1}; 4 or 18 weeks	↓Tumor burden, ↑tumor latency, and ↓number of differentiated SCC; ↓PCNA, p21Cip1/Waf1 and p53 (protein); ↓*Ccnd1*, *Pcna*, *Cdkn1a* and *Gstp* (mRNA); ↓*Bcl2*, *Bcl2l1* and ↑*Bax* and *Bad* (mRNA); ↑*Fasl*, *Cycs*, *Apaf1* and ↓*Cflar* and *Api5* (mRNA); ↑Fas, survivin (nuclear), Bac, Apaf-1, caspases 2L, 6, 8, 9, and 3, Cyt-C and ↓survivin (cytoplasmic), Bcl-2, Mcl-1 (protein)	↓MMP-2, MMP9, TIMP-2, RECK and ↓HIF-1α, VEGF, HDAC-1 and DNMT1 (protein); ↓*Mmp-2*, *Mmp-9*, *Pigf*, *Vegfr1*, *Vegfr2* and ↑*Timp-2* (mRNA)	Priyadarsini et al., 2011[139]
♀ Sprague-Dawley rats treated with DMBA (breast cancer model)	TAM + Quer and TAM-Quer-NPs	TAM + Quer: 3 mg kg^{-1} + 6 mg kg^{-1}	↓Tumor growth; ↑survival rate (after TAM-Quer treatment).	↓MMP-2 and MMP-9 plasma level	Jain et al., 2013[140]

		(1:2 w/w)<br><br>TAM-Quer-NPs: equivalent to 3 mg kg^{-1} TAM (p.o. in a repeated dose of once in 3 days);<br><br>1 month (up to 2 months for survival study)	↑Quer plasma level; ↓tumor growth; ↑survival rate; ↓hepatotoxicity (after TAM-Quer-NPs treatment)		
♀ Sprague-Dawley rats treated with DMBA (breast cancer model)	TAM + Quer and TAM-Quer-SNEDDS	TAM + Quer: 3 mg kg^{-1} + 6 mg kg^{-1} (1:2 w/w)<br><br>TAM-Quer-SNEDDS: equivalent to 3 mg kg^{-1} TAM (p.o. in a repeated dose of once in 3 days); 1 month (up to 2 months for survival test)	↑Survival rate; ↓tumor growth and burden; ↓hepatotoxicity (TAM-quer-SNEDDS treatment was the most effective)	↓MMP-2 and MMP-9 plasma level	Jain et al., 2014[141]
♀ Sprague-Dawley rats treated with DMBA (breast cancer model)	Quer and Quer-SNEDDS	50 and 100 mg kg^{-1} (p.o.); 12 weeks	↑Tumor latency; ↓tumor burden; ↓hepatotoxicity (Quer-SNEDDS treatment was the most effective).	↓MMP-2, MMP-9 plasma level	Jain et al., 2014[142]

			↓TNF-α and IL-6 plasma level		
♀ Athymic nude mice (5 weeks) A375 or SK-MEL-28 xenograft (melanoma model)	Fisetin (alone or in combination with soranefin)	<u>Fisetin:</u> 45 mg kg^{-1} (p.o. 3 times per week)<br><u>Fisetin + soranefib:</u> 45 + 45 mg kg^{-1} (p.o. 3 times per week); 16 or 48 days	↓Tumor growth (highest effect with the mixture); ↓PCNA, Ki-67 and cyclin D1 level; ↑cleaved caspase 3 and PARP; ↑Bcl-2 and ↑Bax; ↓p-MEK1/2, p-ERK1/2, PI3K, p-Akt, p-$mTOR^{Ser2448}$ and ↑PTEN	↓CD31 and VEGF level	Pal et al., 2015[143]
♀ BALB/c nude mice MDA-MB-231 xenograft (breast cancer model)	Rhamnazin	200 mg kg^{-1} day^{-1} (i.g.); 25 days	↓Tumor growth; no effect on body weight; ↓p-$MAPK^{Thr180/Tyr182}$, p-Akt^{S473}, p-$STAT3^{Y705}$ level	↓p-$VEGFR2^{Tyr951}$ level and ↓microvessel density (CD31)	Yu et al., 2015[144]
♀ BALB/c nude mice MCF-7 xenograft (breast cancer model)	Quer	34 mg kg^{-1} (p.o.); 3 weeks	↓Tumor volume and weight; ↑tumor necrosis; ↓number of oncocytes; ↓Ki-67 and vWF level; ↓NFATc3 (protein and mRNA); ↓calcineurin activity	↓Microvessel density in tumor; ↓VEGF, VEGF-R2 (protein and mRNA); ↓vWF level	Zhao et al., 2016[145]
C57BL/6-Tg(TRAMP)8247Ng/J (prostate cancer model)	Quer (alone or together with RSV)	<u>Quer:</u> 60 mg kg^{-1}; 14 and 24 weeks<br><br><u>Quer + RSV:</u> 60 + 600 mg kg^{-1}; 14 and 24 weeks	No effect on diet intake; ↑caspases 8 and 9; ↓Bcl2 (mixture); no effect on *Bax* or *Keap1* (mRNA); ↓*Nrf2* expression (mRNA); ↓hnRNPA1 and ↑NKX3.1	↓*Il6*, *Egfr*, *Igf1* and ↑*Igfbp5* and *Nkx3-1* (mRNA); ↑*Apc* gene expression (only mixture); no effect on IGFBP3 and ↑IGFBP7 (protein); ↓VEGF and EGFR	Singh et al., 2020[146]

			<u>24 weeks' treatment:</u><br>↓Ki-67 (only mixture), PNCA, and survivin (no significant); ↓4HNE<br><br><u>14 weeks' treatment:</u><br>↓Ki-67 and PNCA (only mixture)		
♂ Wistar albino-strain rats (10 – 12 weeks) treated with DENA (hepatic cancer model)	Quer (alone or in combination with sorafenib)	<u>Quer:</u> 50 mg kg^{-1} day^{-1}; 7 weeks<br><u>Quer + sorafenib:</u> 50 + 7.5 mg kg^{-1} day^{-1}; 7 weeks	↓Tissue liver damage; Restoration of body weight; ↓TCh and TG level; ↓ALT, AST, ALP, and conjugated bilirubin; no effect on total protein; ↓CRP, IL-6 and LDH; ↑GSH and ↓MDA; ↓serum PIVKA-II and tissue homogenate AFP; ↓cell proliferation (Ki-67)	↓*Tnfa*, *Vegfa*, *Trp53* and *Nfkb* (mRNA)	Abdu et al., 2022[147]
♂ BALB/c nude mice (6 – 8 weeks) HT-29 xenograft (colon cancer model)	Isoquercetin	17 µg g^{-1} (0.5mL p.o. over a week)	↓Tumor growth;	↓Tumor vascularization; ↑VASH1 and ↓VASH2;	Castilho da Silva et al., 2022[148]
Kunming mice H22 xenograft (hepatic cancer model)	Quer (alone or encapsulated with PVLC-PVA-PEG micelles)	<u>Quer:</u> 150 mg kg^{-1} (p.o.)<br><u>Quer-encapsulated:</u> 100 and 150 mg kg^{-1} (p.o.); 18 days	No effect on body weight; ↓tumor growth; ↓cell proliferation (Ki-67)	↓CD31, VEGF, p-Akt and p-PI3K level	Qi et al., 2022[149]

BALB/c nude (nu/nu) mice Huh-7/shNC and Huh-7/VEGFR2 xenograft (hepatic cancer model)	Quer	50 mg kg^{-1} (p.o.); 20 days	↓Tumor weight and number of circulating cancer cells	↓CD31 level in tumor tissue	Xiong et al., 2024[150]
♂ PTEN KO mice (prostate cancer model)	Quer together with GT or GT + arctigenin	GT administered as drinking water, 0.2% Quer (w/w) in diet, and 30 mg kg^{-1} arctigenin (p.o.); 13 weeks	↓Tumor formation; no effect on food or water intake and absence of toxicity; attenuation of prostate changes; ↓Gleason score related to prostate lesions; ↓AR, p-Akt, t-Akt, and Ki-67	↓CD31 area	Hao et al., 2024[151]
			**Stilbenes**		
♀ C57BL/6 mice (7 weeks) B16 xenograft (melanoma model)	*trans*-RSV or *cis*-RSV	30 µg mL^{-1} in the drinking water; 18 days	Only *trans*-RSV: ↓Tumor growth	↓Microvessel density and CD31 level (mRNA and protein level)	Belleri et al., 2008[152]
♂ Athymic nude mice BALB/cAnNCr-*nu/nu* (5 weeks) LNCaP xenograft (prostate cancer model)	RSV	50 or 100 mg kg^{-1} (enriched diet) daily; 2 weeks before and 7 weeks after cells injection	No effect on body weight; ↓Tumor volume (only at 3 and 4 weeks); ↓PSA level (dose-dependent effect); No effect in cell proliferation; ↓Apoptosis	↑Microvessel formation (PECAM-1 staining) at the 100 mg dose; No differences in VEGF staining	Wang et al., 2008[153]
♂ Athymic nu/nu mice (4-weeks-old) MIA PaCa-2 xenograft (pancreatic carcinoma model)	RSV (alone or together with gemcitabine)	40 mg kg^{-1} once daily (p.o.); 35 days	↓Tumor volume; ↓proliferation (Ki-67), NF-κB, Bcl-2, Bcl-XL, survivin, XIAP, COX-2, c-Myc, pro-caspase 3, cyclin D1, and CXCR4 level	↓Microvessel density (CD31 staining); ↓VEGF, ICAM-1, MMP-9 and survivin level	Harikumar et al., 2009[154]

♀ BALB/c mice (6 – 8 weeks) subjected to injection into the inguinal mammary fat pad (pulmonary of murine mammary carcinoma 4T1-luc cells (breast cancer model)	Piceatannol	10 or 20 mg kg^{-1} day^{-1} (p.o.); 30 days	↓Tumor growth; ↓proliferation (KI-67), cyclin D1, cyclin A, CDK2, and CDK4 level; ↑Apoptotic cells; ↑Bax and cleaved caspase 3, whereas ↓Bcl-2 level; ↓p-NFκB p65, p-STAT3 and HIF-1α expression; ↓M-CSF and MCP-1; ↓Macrophage infiltration	↓VEGF-A, VEGFR-2, VE-cadherin, CD31 and lymphangiogenesis (VEGF-C, LYVE-1) by mRNA, IHC and IF analysis; ↓iNOS, COX-2 and HIF-1α; ↓MMP-9 (tissue and serum) and ↑TIMP-2	Song et al., 2015[155]
Nude mice AsPC1/luc or MP1070/luc xenograft (pancreas cancer model)	RSV (alone or together with EndoCD)	40 mg kg^{-1} resveratrol (p.o.) five times per week; 4 weeks	↓Tumor growth (only in combination); ↑Median survival (only in combination); ↓The amount of collagen, the number of activated PSCs (decreased α-SMA intensity) (only in combination); ↓Number of leukocytes (only in combination); ↑Apoptosis in tumors cells as well as in surrounding endothelial, pancreatic stellate, and immune cells (only in combination)	↓Vessel density (CD31 staining and stroma formation) only in combination	Chen et al., 2017[156]

♂ BALB/C *nu/nu* nude mice (6 weeks) HT-29 xenograft (colon cancer model)	RSV (alone or together with Ginkgetin) in the presence of 5-FU	RSV alone: 480, 960 or 1920 mg kg^{-1} (i.g.) daily; 30 days<br><br>Ginkgetin + RSV: i) 80 +240; ii) 160 + 480; iii) 320 + 960 mg kg^{-1} (i.g.) daily; 30 days	↓Tumor volume and body weight; ↓p-ERK; ↓TNF-α, IL-6 and 5-FU-induced COX-2 level (dose-dependent effect)	↓Microvessel density (CD31 staining) and CD31 protein level	Hu et al., 2019[157]
♀ BALB/c homozygous, Crl:NU(NCr)-Foxn1nu (6 weeks) MBCDF-T xenograft	RSV (alone or together with calcitriol)	1.2 g kg^{-1} (p.o. 3 times a week) and 3 weeks	No significant effect on tumor growth; ↓*Cyp27b1* (RSV and the mixture), *Cyp24a1* (RSV); ↑ *Cyp24a1* (only the mixture); No effect on *Cyp3a44* or *Gpx1* gene expression (mRNA)	↓CD31 area	García-Quiroz et al., 2019[158]
♂ C57BL/6 J (2 weeks) (*Tlr4*$^{-/-}$, *Syk*$^{fl/fl}$, *Alb*^{Cre}, *Cd4*^{Cre}, *LyzM*^{Cre} and *Cd19*^{Cre}) with induced liver fibrosis or hepatocellular carcinoma (CCL_4 + Diethylnitrosamine model)	Piceatannol	20 mg kg^{-1} (p.o.); 12 weeks	↓Liver fibrosis and α-SMA level; ↓serum transaminase levels and liver hydroxyproline level; ↓tumor nodules; ↓Liver/Body weight; ↓CD45+ cells; ↓BCL-xl and Smad4 protein expression; ↓oncogenic genes: *Apaf1*, *Bcl2l11*, *Birc3 (*apoptosis) *Mki-67* and *Ccnd2 (*cell cycle	↓*Angpt2* and *Ccl2* (mRNA) in liver	Torres-Hernández et al., 2019[159]

			regulation), and *Map2k1*, *Serpinb2 (*cellular senescence); ↑p16 and p53 protein expression; ↑IFN-γ, IL-10, and ↓TNF-α in liver		
C57BL/6-Tg(TRAMP)8247Ng/J (prostate cancer model)	RSV	600 mg kg^{-1}; 14 and 24 weeks	No effect on diet intake; ↑caspases 8 and 9; ↓*Bcl2* (mRNA); no effect on *Bax* or *Keap1* (mRNA); ↓*Nrf2* expression (mRNA)<br><u>- 24 weeks treatment:</u><br>↓Ki-67 (only mixture), PNCA and survivin (no significant); ↓4HNE; ↓hnRNPA1 and ↑NKX3.1<br><br><u>- 14 weeks treatment:</u><br>↓Ki-67 and PNCA (no significant)	↓*IL-6*, *Egfr*, *Igf1* and ↑*Igfbp5*, *Nkx3-1* and *Apc* (mRNA); ↓No effect on *Egfr3* expression; no effect on IGFBP3; ↑IGFBP7	Singh et al., 2020[146]
♀ Balb/c mice (6 weeks) H-357-CSCs xenograft (oral cancer model)	RSV nanoparticle-based polymer	40 mg kg^{-1} day^{-1} (dissolved in PBS) (p.o.) every other day; 30 days	↓Tumor volume; ↑Recovery from lost body weight; ↓Expression of CD44 in the liver, CXCR4 and Nanog in the kidney, and CXCR4 in brain tissues	↓VEGF-A in brain tissues	Pradhan et al., 2021[160]

♂ C57BL/6J mice (4 weeks) LLC xenograft (lung cancer model)	RSV	25 mg kg^{-1} day^{-1} (p.o.) in drinking water; 1 week before and 3 weeks after LLC implantation	↓Tumor volume and weight; ↓proliferation (not significant); ↑apoptosis, autophagy, and cross-talk between CAFs in LLC tumor masses; ↓infiltration of tumor-associated immune-cells in LLC-tumor microenvironment CD163, CD68 and CD3); ↓necrosis α-SMA and p62	↓Blood vessel density and integrity (CD31; not significant)	Savio et al., 2022[161]
♂ Prostate-specific MTA1-overexpressing mice (*R26^{MTA1}*; *Pten$^{+/f}$*) (3 weeks)	Gnetin or Pterostilbene	<u>Gnetin C:</u> 35 or 70 mg kg^{-1} (p.o.)<br><br><u>Pterostilbene:</u> 70 mg kg^{-1} (p.o.); 17 weeks	No effect on body weight or food intake<br><br>Delayed the progression of preneoplastic lesions; ↓Severity and number of prostatic intraepithelial neoplasia foci; ↓Cell proliferation; ↓IL-2 production in serum; ↑Phosphatase and tensin homolog expression; ↓MTA1 level (mRNA and protein); ↑PTEN level (mRNA and protein; only significant	↓Vessels area (CD31 staining)	Parupathi et al., 2022[162]

			with pterostilbene); no effect on p-Akt/t-Akt		
Prostate-specific MTA1 overexpressing mice *R26*^{MTA1}; *Pten*$^{+/f}$; mice (3 weeks)	Pterostilbene	100 mg kg^{-1} (p.o.) daily; 17 weeks	↓Prostate size; ↓Number of glands involved in prostatic intraepithelial neoplasia; ↓proliferation; ↓MTA1 level and associated CyclinD1 and Notch2 levels;↓IL-6, IL-1β and miRNA level (serum)	↓Vessels area (CD31 staining)	Hemani et al., 2022[163]
♀ Balb/c mice (6 weeks) H-357-CSCs xenograft (oral cancer model)	Polymer-based RSV-nano particle	40 mg kg^{-1} (p.o.) every other day; 30 days	↓Tumor volume; ↑recovery from lost body weight; IL-6, PD-L1, p-JAK2, and p- STAT3; ↓CD133, CXCR4, NANOG level (kidney, liver, brain, neck lymph nodes, and lung)	↓ANG-1 and VEGF-A level (kidney, liver, brain, neck lymph nodes, and lung)	Pradhan et al., 2023; 2024[164,165]
Inbred BALB/c mice (8 – 12 weeks) Ehrlich's ascites carcinoma in the right femur	RSV (alone or together with cisplatin)	50 mg kg^{-1} day^{-1} (p.o.); 5 days	↑Survival rate and regression of tumor volume (only in combination); ↓HDACs level, arginase activity, modulation of HSP70 and HSP90 level and ↑NO concentration and iNOs level in tumor tissue (only in combination)	↓Number of blood vessels; ↓VEGF and HIF-1α, level (only in combination); modulation of MMP2 and MMP9	Kučan et al., 2023[166]
			**Curcuminoids**		

♂ Nude mice (6 – 8 weeks) LNCaP xenograft (prostate cancer model)	Curc	2% (w/w) enriched diet; 4 or 6 weeks	↓Proliferation (BrdU); ↓tumor volume; ↑apoptosis	↓Microvessel density (CD31)	Dorai et al., 2001[167]
♀ Athymic nude mice (NCr-*nu*) HeyA8 and SKOV3ip1xenograft (ovarian cancer model)	Curc (alone or together with docetaxel)	Dose-dependent experiments: 0.1, 0.5, 1, and 2 g kg^{-1} (gavage) once daily; 1, 2, 3, or 6 days<br><br>Antitumor experiments: 500 mg kg^{-1} day^{-1} (gavage) once daily; ~3 – 4 weeks	↓Tumor weight, mass and burden; ↓Tumor nodules; ↓proliferation; ↑apoptosis; ↓NF-κB activation and p-STAT3; ↓COX-2	↓Microvessel density (CD31); ↓VEGF, IL-8, and MMP-9 level	Lin et al., 2007[168]
♂ Athymic nude mice *nu/nu* (4 weeks) MIA PaCa-2 xenograft (prostate cancer model)	Curc (alone or together with gemcitabine)	1 g kg^{-1} (p.o.) once daily; 28 days	↓Tumor volume; ↓proliferation (Ki-67); ↓NF-κB, ↓cyclin D1, c-Myc, COX-2, survivin, Bcl-2, Bcl-xL, and IAP-1 in pancreatic tumor tissues (highest effect in combination with gemcitabine)	↓Microvessel density (CD31), VEGF, ICAM-1, and MMP-9 (highest effect in combination with gemcitabine)	Kunnumakkara et al., 2007(A)[169]
♂ Athymic nude mice *nu/nu* (4 weeks) HCT 116 xenograft (colon cancer model)	Curc (alone or together with γ-radiation)	1 g kg^{-1} (p.o.) daily; 30 days	↓Tumor volume; ↓proliferation (Ki-67), NF-κB, cyclin D1, c-myc, COX-2, Bcl-2, Bcl-xL, cFLIP and IAP-1 level	↓Microvessel density (CD31), ICAM-1, survivin, VEGF and MMP-9	Kunnumakkara et al., 2008[170]

			(protein); ↑caspases 3 and 9		
BALB/c nude mice (4 – 6 weeks) LNCaP xenograft (prostate cancer model)	Curc	30 mg kg^{-1} (oral injection in normal saline); three times per week for 6 weeks	↓Tumor growth; ↑apoptosis; ↓proliferation (PCNA and Ki-67); ↑TRAIL-R1/DR4, TRAIL-R2/DR5, Bax, Bak, $p21^{Cip1/Waf1}$, and p27/KIP1 level; ↓NF-κB, p-p65, COX-2 and IL-8 level; ↓cyclin D1, uPA, Bcl-2, and Bcl-XL level	↓Blood vessel number (H&E, CD31 and vWF); ↓VEGFR-2 positive circulating endothelial cells; ↓VEGF, MMP-2, and MMP-9	Shankar et al., 2008[171]
♂ BALB/c nude mice HepG2 xenograft (hepatic cancer model)	Curc or THC	0.3 and 3 g kg^{-1} day^{-1} (p.o.); 21 days		↓Capillary vascularity (dose-dependent effect); amelioration of pathological angiogenic features (microvascular dilatation, tortuosity, and hyper-permeability)	Yoysungnoen et al., 2008[172]
♂ Kunming mice (clean) H22 xenograft (hepatic cancer model)<br><br>♂ C57BL/6 (clean) B16 xenograft (melanoma model)<br><br>♂ BALB/c-nu (SPF) SW480 xenograft (colon cancer model)	Curc or Cur-K30 solid dispersion	<u>Curc:</u> 200 mg kg^{-1} twice a day (intragastric administration); 6–10 days<br><br><u>Curc-K30:</u> 25, 50, 100 or 200 mg kg^{-1} twice a day (intragastric administration); 6–10 days	↓Tumor growth in the models tested;	↓Microvessel density (CD34) and VEGF level in SW480 xenograft nude mice	Chen et al., 2010[173]

♀ BALB/c nude mice (6 – 8 weeks) ACC-M xenograft (high metastasis adenoid cystic carcinoma model)	Curc	1 g kg^{-1} day^{-1} (p.o.) daily; 28 days	↓NF-κB and p-S6 activation ACC-M tissues	↓Microvessel density (CD31-positive staining); ↓VEGF and MMP-9 level	Sun et al., 2011[174]
♂ Golden hamsters (4 – 6 weeks) subjected to CCA via a combination of NDMA and *Opisthorchis viverrini* infection	Curc	1% (w/w; ~65 mg day^{-1}) in enriched diet; 21 days and 1, 2, 3, 4, 5 and 6 months	↓CCA incidence; ↑survival rate; ↓NF-κB, AP-1 and STAT-3 activation; ↓COX-2 and iNOS expression; ↓8-nitroguanine and 8-oxo-dG level; ↓bcl-2 and bcl-xL, cyclin D1 and c-Myc; ↑apoptosis, caspase activation, and PARP cleavage	↓Microvessel density (CD31), VEGF, MMP-9, and ICAM-1 level	Prakobwong et al., 2011[175]
♀ C57BL/6 mice (3 weeks) LLC xenograft (lung cancer model)	Curc	2 or 4% (w/w) enriched diet; 5 weeks before and 10 days after LLC injection	↑Metastatic tumor cross-sectional area and volume; No changes in size of metastatic tumors; ↑ IL-1β and MCP-1 in plasma	↑Angiogenin, bFGF, and VEGF	Yan, 2013[176]
♀ Nude (Foxn1*nu*/*nu*) mice (8 weeks) injected with MIA PaCa-2 cells transfected with RPF	Curc	0.6% (w/w) enriched diet; 6 weeks	↓NF-κB activation in orthotopic tumor tissue samples; ↓COX-2, cyclin D1, IKKα, and IKKβ	↓VEGF and MMP-9	Bimonte et al., 2013[177]
♂ Sprague-Dawley rats treated with CCl_4 (liver fibrosis model)	Curc	200 mg kg^{-1} (p.o.); 6 weeks	↓CCl_4-induced liver fibrosis, hydroxyproline content, hepatic stellate cell activation; ↓COX-2 and collagenIII level	↓Microvessel density (CD31 and vWF); ↓VEGFR-2, HIF-1α, VEGFR-1, PIGF, MMP-9 and ICAM-1 level	Yao et al., 2013[178]

			(protein); ↓*Col1α1* (mRNA)	(protein); ↓sinusoidal capillarization	
♀ C57BL/6 mice (18 – 20 g) LLC xenograft (lung carcinoma model) subjected to ischemia	Curc	300 mg kg^{-1} day^{-1} (p.o.); 22 days	↓Tumor weight; ↑survival rate; ↑Post-ischemic blood recirculation	↑Number of blood vessels in ischemic limb; ↓Capillary regeneration in cancer tissues (CD31); ↓Lung cancer progression via regulation of the HIF1α/mTOR/VEGF/VEGFR cascade	Fan et al., 2014[179]
♂ BALB/c nude mice (20 – 25 g) CaSki xenograft (cervical cancer model)	Curc	0.5, 1.0, and 1.5 g kg^{-1} day^{-1} (p.o.); 30 days	↓Tumor growth (at 1.0 and 1.5 g kg^{-1} doses; dose-dependent)	↓Microvessel density (CD31-positive); ↓VEGF, COX-2, and EGFR level (at 1.0 and 1.5 g kg^{-1} doses; dose-dependent)	Yoysungnoen-Chintana et al., 2014[180]
♀ BALB/c nude mice (20 – 25 g) subcutaneously injected with cervical cancer (CaSki) cells	THC	100, 300, or 500 mg kg^{-1} (p.o.); 30 days		↓Tumor angiogenesis and Microvessel density (CD31); ↓VEGF, VEGFR-2, and HIF-1α level (protein)	Yoysungnoen et al., 2015[181]
♂ Albino rats treated with DENA (hepatocellular carcinoma model)	Curc	100 mg kg^{-1} (p.o.); 15 days	Curc normalizes the serum values of liver marker enzymes (AST and ALT), lipid peroxidation and improves liver architecture; ↑GST activity; ↓Akt; ↑*Casp-3* (mRNA)	↓TGF-β	Abouzied et al., 2015[182]
♂ Wistar rats treated with NDEA (hepatocellular carcinoma model)	Curc	15 mg kg^{-1} (p.o.); five days a week for 15 weeks	↓Serum α-fetoprotein and α-L-fucosidase levels (serum); ↓*Ggt1* and *Hsp90b1* (mRNA);	↓VEGF level in serum	Ahmed et al., 2015[183]

			↓Proliferation (PCNA and Ki-67); ↑apoptosis (DNA fragmentation)		
BALB/c nude mice BEL-7402 xenograft (hepatocellular carcinoma model)	Curc: i) alone, ii) together with sorafenib, and iii) together with sorafenib encapsulated in SCN	80 mg kg^{-1} (p.o.; alone or with sorafenib); 25 days<br><br>8 mg mL^{-1} in SCN	↓Tumor weight and volume (only observed in combination and SCN); ↓proliferation (Ki-67; only in SCN); detection of SCN at the intestinal and systemic level	↓Neovasculature in tumor tissues (CD31; only in SCN)	Cao et al., 2015[184]
♀ Athymic nude *nu/nu* mice (3 – 4 weeks) FaDu xenograft (pharyngeal carcinoma model)	Curc (alone or together with KU-55933)	200 mg kg^{-1} day^{-1} (p.o.) daily; 4 weeks	↓Tumor growth; ↑cleaved-caspase 3 positive cells; ↓Ki-67-positive cells in tumor; ↓SIRT1 pathway	↓CD31 level; ↓MMP-2 and VEGF (mRNA and protein)	Hu et al., 2015[185]
♂ Balb/c nude mice (6 – 8 weeks) HT-29 xenograft (colon cancer model)	Curc or turmeric ethanolic extract (alone or together with bevacizumab)	Curc.: 75 mg kg^{-1}; 30 days<br><br>Turmeric ethanolic extract: 400 mg kg^{-1}; 30 days	No effect on body weight; ↓tumor volume (in combination with bevacizumab); ↓Tumor weight (only turmeric in combination with bevacizumab); no significant effects on COX-2, AKT1, cyclin D1, and survivin; ↑survival rate (only turmeric in combination with bevacizumab); no effect on AST, ALT, troponin and CK; ↑neutrophils only in combination with	↓Formation of neovasculature in tumor xenografts (CD31 and vWf)	Yue et al., 2016[186]

			bevacizumab); no significant effect on apoptosis		
Athymic *nu/nu* (BALB/c) mice (6 weeks) A549 xenograft treated with TM	Curc	100 mg kg^{-1} (gavage) every other day; 3 weeks	↓Tumor growth and size; ↓serum copper levels; ↓proliferation (Ki-67) and ERK activation; ↑apoptosis and cleaved caspase 3	↓Vascularization, VEGF level (serum), and $CD31^{+}$ cells in tumor	Zhang et al., 2016[187]
$ATM^{-/-}$, $ATM^{+/-}$ and $ATM^{+/+}$ mice Hep2 xenograft (head and neck squamous carcinoma model)	Curc	200 mg kg^{-1} day^{-1} (p.o.) daily; 4 weeks	No effect on body weight; ↓Tumor growth; ↓proliferation (Ki-67); ↑caspase 3; ↑p-ATM, p-Chk1 and p-Cdc25c level	↓Microvessel density (CD31); ↓HIF-1α level; ↓VEGF, MMP-9 and MMP-2 level	Hu et al., 2017[188]
♀ CB17/SCID mice (6 – 8 weeks) subcutaneously injected with fresh human colorectal carcinoma fragments obtained intraoperatively from patients	Curc (alone or together with EGCG)	50 mg k^{-1} day^{-1} (p.o.) every other day; 4 weeks	↓Tumor weight and volume; ↓p-JAK, p-STAT3 and IL-8 (highest effect observed in combination)	↓Microvessel density (CD31); ↓Hemoglobin (highest effect observed in combination)	Jin et al., 2017[45]
♂ Wistar rats (6 – 7 weeks) treated with DMBA (skin tumor model)	Turmeric paste (*Curcuma longa*)	5% (w/w) enriched diet together with topical application (2 g per rat); 28 days	↓Tumor volume; ↓mitotic indices: ↓Cell proliferation; ↓proliferation (PCNA); ↑apoptotic index; ↑caspases 2, 3, 8, and 9, PARP, and p53 level; ↓NF-κB; ↓CD4+, CD8+, and NK cells population	↓VEGF	Rajmani et al., 2017[189]

Athymic nude mice (6 – 8 weeks) NCI-H460 xenograft (ectopic lung cancer model) or H1975 xenograft (orthotopic lung cancer model)	Curc	100 mg kg^{-1} (gavage) daily; 28 or 60 days	↓Tumor weight and size (ectopic xenograft model); ↓Tumor weight; ↑Survival rate; ↓p-STAT3/t-STAT-3, p-JAK/t-JAK, Bcl-xL, cyclin D1 (ectopic xenograft model)	↓Hemoglobin content; ↓*Pecam-1* and *Eng* (mRNA); ↓VEGF (ectopic xenograft model)	Xu et al., 2017[190]
♂ BALB/c nude mice (5–6 weeks) HepG2 xenograft (hepatic cancer model)	Curc (alone or together with AS-IV)	100 mg kg^{-1} day^{-1} (gavage) daily; 21 days	↓Tumor weight; ↑miR-122 and ↓miR-221	↓Microvessel count (CD34); ↓HGF, TF and FVII (mRNA and protein); ↓bFGF, MMP2 and VEGF level	Zhang et al., 2017[191]
♂ nude mice (4 – 5 weeks) H22 xenograft (hepatocarcinoma model)	Curc	50 or 100 mg kg^{-1} day^{-1} daily; 2 weeks	↓Tumor growth and weight (dose-dependent effect); ↓PI3K and Akt (Ser/Thr kinase 1) level (mRNA and protein)	↓VEGF level (mRNA and protein)	Pan et al., 2018[192]
♂ BALB/c nude mice (4 – 5 weeks) HepG2 xenograft (hepatic cancer model)	Curc (alone or together with APS)	100 mg kg^{-1} day^{-1} (gavage) daily; 21 days	↑Behavioral state, appetite, and gastrointestinal reactions; ↓Tumor growth and weight (only in combination); ↑NG2 level	Improvement of morphological structure of tumor vessels (only in combination); ↓branches and areas of the vessels; ↓CD31 expression (only in combination)	Tang et al., 2019[193]
♂ BALB/c *nu/nu* mice (5 weeks) HepG2 xenograft (hepatic cancer model)	Curc (alone or together with TGn)	200 mg kg^{-1} day^{-1} (p.o.) daily; 21 days	↓Tumor volume; ↓PD1 (only in combination) and PD1-L1 ; ↓NF-κB and iNOS level	↓MMP9 level	Deng et al., 2020[194]

♂ BALB/c *nu/nu* mice (3 – 4 weeks) HepG2 xenograft (hepatic cancer model)	Curc	120 or 240 mg^{-1} kg^{-1} day^{-1} (p.o.) daily; 15 days	↓Tumor volume and weight; ↓MDSCs ($CD11^{+}$ $Gr\text{-}1^{+}$) and GM-CSF and G-CSF level; ↓TLR4/NF-κB signaling pathway; ↓IL-6, IL-1β, PGE_2 and COX-2 level in tumors	↓VEGF, αSMC (an arterial smooth muscle cell marker), and CD31 level (tumor tissue)	Tian et al., 2021[195]
♀ Balb/c mice (6 weeks) subcutaneously injected with fresh human breast cancer fragments obtained intraoperatively from patients	Curc (alone or together with Quinacrine)	20 mg kg^{-1} day^{-1} (p.o.); 2 weeks		↓New blood vessel formation	Nayak et al., 2023[196]
♀ Nude (Foxn1*nu/nu*) mice; MBA-MB-231 xenograft	Curc	0.6% (w/w) enriched diet; 6 weeks	↓Tumor growth, NF-kB, cyclin D1 and p65	↓Microvascular density (FITC-dextran staining) and PECAM-1	Bimonte et al., 2015[197]
♀ BALB/c homozygous, Crl:NU(NCr)-Foxn1nu; MBCDF-T cells xenograft	Curc (alone or together with calcitriol)	40 mg kg^{-1} (p.o. every day); 3 weeks	Effects of curcumin and calcitriol: ↓tumor volume; ↓*Cyp27b1* (Curc and the mixture), *Cyp24a1* (Curc); ↑ *Cyp24a1*, *Cyp3a44*, *Gpx1* gene expression (only the mixture); no significant effect of Curc on *Cyp3a44* and *Gpx1* (mRNA)	↓Microvessel density (ITGB3) in combination with calcitriol	García-Quiroz et al., 2019[158]
♂ Wistar rats (5 – 6 weeks) treated with MNNG (gastric cancer model)	Curc alone or together with *Oldenlandia diffusa* extract	200 and 400 mg kg^{-1} enriched (normal and keto) diet; 18 weeks (6	Amelioration of the MNNG-induced reduction of b.w.; improvement of MNNG-induced gastric	↓VEGF level	Xiao et al., 2022[198]

		weeks of cancer induction and 12 weeks of treatment)	damage; ↓mortality and tumor area; ↑SOD and CAT; Amelioration of the MNNG-induced reduction of MDA level; ↓TNF-α and IL-β level; ↓NF-κB and ↑Nrf2 level; ↓Akt and mTORC1 level; ↓*Bcl-2* and *Caspase 3* and ↑*Bax* and miRNA-340 (mRNA)		
♂ Golden Syrian hamster (5 weeks) treated with DMBA (oral cancer model)	Curc (alone or together with green tea)	10 mmoL diluted in paraffin and applied three times per week in the left pouch of the mouth; 18 weeks	Improvement of histological lesions; ↓proliferation (CD34); ↑apoptosis (highest effect in combination with green tea)	↓Microvessel density (CD34)	Saleh et al., 2023[49]
♂ and ♀ Wistar rats treated with NDEA (hepatotoxic model)	EECL and Curc (alone or together with NDEA)	<u>EECL:</u> 200, 400 and 600 mg kg^{-1} <u>Curc:</u> 200 mg kg^{-1}; 2 weeks	<u>Effects in males:</u> ↓*Mapk* expression (mRNA) by Curc and EECL at 200 and 400 (no effect at 600 mg kg^{-1}) in the presence of NDEA; ↑*Mapk* expression (mRNA) by Curc and EECL at 200 and 400 (no effect at 600 mg kg^{-1}) in the absence of NDEA; ↑*Afp* expression (mRNA) by Curc and EECL (400 mg	<u>Effect in male and female rats:</u> ↓*Vegf* expression (mRNA) by EECL (200 – 400 mg kg^{-1}) and Curc, and slight ↑ at 600 mg kg^{-1} in the presence of EECL; ↑*Vegf* expression (mRNA) by Curc and EECL in the absence of EECL	Onifade et al., 2023[199]

kg^{-1}), ↓ by EECL at 600 mg/kg and no effect at 200 mg kg^{-1} in the presence of NDEA; ↓*Afp* expression (mRNA) by EECL and ↑ by Curc in the absence of NDEA; ↓*Xiap* expression (mRNA) by Curc and EECL (400 – 600 mg kg^{-1}) and no effect at 200 mg kg^{-1} in the presence of NDEA; ↓*Xiap* expression (mRNA) by EECL and Curc, and no effect of EECL at 200 mg kg^{-1} in the absence of NDEA<br><br><u>Effects on females:</u><br>↓*Mapk* expression (mRNA) by Curc and EECL at 200, 400, and 600 mg kg^{-1} in the presence of NDEA; ↑*Mapk* expression (mRNA) by EECL at 200 mg kg^{-1}; ↓*Afp* expression (mRNA) by EECL (400 – 600 mg kg^{-1}) and ↑by Curc and EECL (200 mg kg^{-1}) in the presence of NDEA; ↓*Afp*	<u>Effect in male rats:</u><br>↓*Egfr* expression (mRNA) by Curc and EECL (200 – 600 mg kg^{-1}) in the presence of NDEA; ↓*Egfr* expression (mRNA) by Curc and EECL (400 and 600 mg kg^{-1}) and ↑ by EECL at 200 mg kg^{-1} in the absence of NDEA<br><br><u>Effect in female rats:</u><br>↓*Egfr* expression (mRNA) by Curc and EECL (200 and 600 mg kg^{-1}), ↑by EECL at 400 mg kg^{-1} in the presence of NDEA; ↑*Egfr* expression (mRNA) by Curc and EECL (400 and 600 mg kg^{-1}), and ↓by EECL at 200 mg kg^{-1}

		expression (mRNA) by EECL and no effect by Curc; ↓*Xiap* expression (mRNA) by EECL (400 – 600 mg kg^{-1}) and ↑ by Curc and EECL (200 mg kg^{-1}) in the presence of NDEA; ↓*Xiap* expression (mRNA) by EECL and Curc in the absence of NDEA	

Abreviattions: 2-HF: 2'-hydroxyflavanone; **4HNE:** 4-hydroxynonenal; **8-OH-dG:** 8-hydroxy-2'-deoxyguanosine; **8-oxo-dG:** 8-oxo-7,8-dihydro-2'-deoxyguanosine; **ABCA1:** ATP-binding cassette transporter; **AFP:** alpha-fetoprotein; **ALP:** alakaline phosphatase; **ALT:** alanine aminotransferase; **ANG:** angiopoietin; **AOM:** azoxymethane; **APAF1:** apoptotic peptidase activating factor 1; **APS:** Astragali polysaccharide; **AR:** androgen receptor; **AST:** aspartate aminotransferase; **AS-IV:** Astragaloside IV; **ATM:** ataxia-telangiectasia mutated; **BBN:** N-butyl-(-4-hydroxybutyl) nitrosamine; **bFGF:** basic fibroblasts growth factor; bLF: bovine lactoferrin; **BM:** bone marrow; **BRB:** black raspberries; **BrdU:** bromodesoxyuridine; **BT:** black tea; **b.w.:** body weight; **CA9:** carbonic anhydrase 9; **CA 15-3:** cancer antigen 15-3; **CAFs:** cancer-associated fibroblasts; **CAT:** catalase; **CCA:** cholangiocarcinogenesis; **CD:** conjugated dienes; **CD31/PECAM1:** platelet endothelial cell adhesion molecule 1; **CDC-2:** cell division control protein 2; **CDK:** Cyclin-dependent kinase 2; **CEA:** carcinoembryonic antigen; **cFLIP:** cellular FLICE-like inhibitor protein; **CHD5:** Chromodomain-helicase-DNA-binding protein 5; **CK:** creatin kinase; **COX-2:** cyclooxygenase-2; **CRP:** C-reactive protein; **Curc:** curcumin; **CYR61:** cysteine-rich angiogenic inducer 61; **Cyt-C:** cytochrome-C; **DAZ:** daidzein; **DENA:** diethylnitrosamine; **DHT:** dihydrotestosterone; **DMBA:** 7,12-mimethylbenz[a]anthracene; **DMH:** 1,2 dimethylhydrazine; **DNMT:** DNA-methyltransferase; **DR:** death receptor; **DTD:** DT-diasphore; **EA:** ellagic acid; **EAC:** Ehrlich ascites carcinoma; **EECL:** Ethanolic extract of *Curcuma longa*; **EGCG:** epigallocatechin gallate; **EGF:** epidermal growth factor; **EndoCD:** endostatin and cytosine deaminase linked to uracil phosphoribosyltransferase; **ENL:** enterolactone; **eNOS:** endothelial nitric oxide synthase; **ERα:** estrogen receptor alpha; **ERK:** extracellular signal-regulated kinases; **ET-1:** endothelin-1; **FITC:** fluorescein isothiocyanate; **FLT-4:** vascular endothelial growth factor receptor 3; **FS:** flax seed; **FSE:** fermented soybean extract; **FVII:** coagulation factor VII; **GCP:** genistein combined polysaccharide; **G-CSF:** granulocyte-colony stimulating factor; **GEN:** genistein; **GGT:** γ-glutamyl transferase; **GLUT-1:** glucose transporter 1; **GM-CSF:** granulocyte-macrophage colony-stimulating factor; **GPx:** glutathione peroxidase; **GSH:** glutathione; **GSI:** genistin-rich soy isoflavones; **GSK-3β:** Glycogen synthase kinase-3 beta; **GST:** glutathione-S transferase; **GST-P:** placental glutathione-S transferase; **GT:** green tea; **Hb:** haemoglobin; **HCC:** Hexahydroxycurcumin; **HDAC:** Histone deacetylase; **HDL:** high density lipoprotein; **Hes:** hesperidin; **HGF:** hepatocyte growth factor; **HIF-1α:** hipoxia inducible factor 1 alpha; **HO-1:** heme oxidase 1; **Hmox1:** heme oxygenase 1; **HuR:** human antigen R or ELAV like protein 1; **HSC70:** heat shock 70 kDa protein 8 or heat shock cognate 71 kDa protein; **HSP90:** heat shock protein 90; H&E: haematoxylin and eosin; **IAP-1:** inhibitor of apoptosis protein 1; **ICAM-1:** intracelular Adhesion Molecule 1; **IDSP:** isoflavone-depleted soy protein; **IGF-1:** insulin growth factor 1; **IFG1R:** insulin growth factor 1

receptor; **iNOS:** inducible nitric oxide synthase; **IR:** insulin receptor; **ISF:** isoflavone; **ITGB3:** integrin beta 3; **JNK:** c-Jun NH2-terminal kinase; **KLK6:** kallikrein 6; **KO:** Knock-out; **LDH:** lactate dehydrogenase; **LLC:** Lewis lung carcinoma cells; **L-NAME:** NG-nitroarginine methyl ester; **LOOH:** lipid hydroperoxides; **LPN:** lipid-polymer nanoparticle; **M1/M2:** pro-inflammatory macrophages/anti-inflammatory macrophages; **MCP-1:** monocyte chemoattractant protein 1; **MDA:** malondialdehide; **MDSCs:** myeloid-derived suppressor cells; **MEK:** mitoen-activated protein kinase kinase; **miR:** microRNA; **MLC:** myosin light chain; **MMP:** matrix metalloproteinase; **MNNG:** N-methyl-N′-nitro-Nnitrosoguanidine; **MPO:** myeloperoxidase; **MTA1:** metastasis associated protein 1; **mTOR:** mammalian target of rapamycin; **MTX:** methotrexate; **Nar:** naringenin; **NG2:** neural/glial antigen 2; **n.d.:** not described: **NDEA:** *N*-nitrosodiethylamine; **NFATc3:** nuclear factor of activated T cell 3; **NK:** natural killer; **NKX3.1:** NK3 homeobox-1; **NMBA:** *N*-nitrosomethylbenzylamine; **NNK:** 4-(methylnitrosamino)-1-(3-pyridyl)-1-butanone; **NO:** nitric oxide; **nNOS:** neuronal nitric oxide synthase; **NPs:** nanoparticles; **p-:** phosphorylated; **PARP:** Poly (ADP-ribose) polymerase; **PAS:** periodic acid-Schiff; **PCNA:** proliferating cell nuclear antigen; **PDK-1:** phosphoinositide-dependent kinase-1; **PD1:** programmed cell death 1; **PD-L1:** programmed cell death ligand 1; **PECAM-1/CD31:** platelet endothelial cell adhesion molecule; **PGG:** penta-*O*-galloyl-β-D-glucose; **Pgp:** P-glycoprotein; **PH:** pleckstrin homology domain; **PIGF:** placental growth factor; **PhIP:** 2-amino-1-methyl-6-phenylimidazo[4,5-b]pyridine; **PI3K:** phosphatidylinositol 3-kinase; **PIVKA-II:** protein induced by vitamin K absence-II; **PMF**: polymethoxyflavones; **PR:** progesterone receptor; **PSA:** prostate specific antigen; **PTEN:** phosphatase and tensin homolog; **Quer:** quercetin; **RECK:** reversion inducing cysteine rich protein with kazal motifs; **RhoA:** Ras homolog family member A; **ROCK:** Rho-associated coiled-coil containing protein kinases; **RPF:** red fluorescent protein; **RSV:** resveratrol; **SCN:** selfassembled nanoparticles; **SDG:** secoisolariciresinol diglucoside; **SDF-1:** stromal cell-derived factor 1; **SDPN:** sophorolipid-associated membrane-biometric choline phosphate-poly(lactic-*co*-glycolic) acid hybrid nanoparticle; **SIE:** soy isoflavone extract; **SIRT:** sirtuin; **αSMA:** alpha smooth muscle actin; **SMAD:** suppressor of mothers against decapentaplegic; **SMO:** smoothened; **SNEDDS:** self-nanoemulsifying drug delivery system; **SOD:** superoxide dismutase; **SP:** soy protein; **SPC:** soy phytochemical concentrate; **SPE:** soy protein extract; **SPI:** soy protein isolate; **SCC:** squamous cell carcinoma; **STZ:** streptozotocin; **TAC:** total antioxidant capacity; **TAM:** tamoxifen; **TBARS:** thiobarbituric acid reactive subtances; **TCh:** total cholesterol; **TF:** thrombosis-related tissue factor; **TG:** triglycerides; **TGF-β1:** transforming growth factor beta 1; **TGn:** total ginsenosides; **THC:** tetrahydrocurcumin; **Tie2:** angiopoietin-1 receptor; **TIMP:** tissue inhibitor of metalloproteinases: **TM:** ammonium tetrathiomolybdate; **TNF-α:** tumor necrosis factor alpha; **TRAP:** transgenic rat for adenocarcinoma of prostate; **TRAIL-R:** death receptor; **TSP:** thrombospondin; **TTF-1:** 5,2,4´-trihydroxy-6,7,5´-trimethoxyflavone; **uPA:** urokinase plasminogen activator; **VASH:** vasohibins; **VEGF:** vascular endothelial growth factor; **VEGFR2:** vascular endothelial growth factor receptor 2; **vWF:** von Willebrand protein.

[a]**Green tea (poly)phenols:** 62% EGCG, 24% epicatechin 3-gallate, 5% epigallocatechin, 6% epicatechin
[b]**Nutrient mixture:** 0.7 g Vit. C (as ascorbic acid, palmitate ascorbate, Mg and Ca), 1 g L-Lys, 0.75 g L-Pro, 0.5 g L-Arg, 0.2 g N-acetyl-Cys, 3×10^{-2} g Se, 2×10^{-3} g Cu, 1×10^{-3} g Mn and 1 g standardized green tea extract (80% (poly)phenols).
[c]**Polyphenon-E:** 11% epicatechin, 7% epicatechin gallate, 9% epigallocatechin, 60% EGCG, 5% gallocatechin gallate, 1% catechin gallate, and 1% catechins
[d]**Nutrient mixture:** 0.7 g Vit. C (as ascorbic acid, palmitate ascorbate, Mg and Ca), 1 g L-Lys, 0.75 g L-Pro, 0.5 g L-Arg, 0.2 g N-acetyl-Cys, 3×10^{-2} g Se, 2×10^{-3} g Cu, 1×10^{-3} g Mn and 1 g standardized green tea extract (80% (poly)phenols, 60% catechins, 35% EGCG, 1% caffeine).

[e]**Polyphenon-E (w/w):** 0.4% epicatechin, 1.4% EGCG, 0.1% epicatechin gallate, 0.2% gallocatechin gallate, 0.32% free theaflavin, 0.14% theaflavinmonogallate-A, 0.15% theaflavinmonogallate-B, 0.24% theaflavindigallate, 35.6% tannin and 4.9% caffeine.
[f]**BTF-35 (% w/w):** 0.1 epigallocatechin, 0.2 epicatechin, 2.6 EGCG, 2.1 epicatechin gallate, 0.3 gallocatechin gallate, 0.1 catechin gallate, 0.1 catechin, 7.1 free theaflavin, 8.3 theaflavinmonogallate-A, 2.6 theaflavinmonogallate-B, 9.8 theaflavindigallate, and 0.5 caffeine.
[g]**Polyphenon-E (w/w):** 65% EGCG, 10% epicatechin, 5% epigallocatechin, 5% epicatechin gallate, and 0.5% caffeine.
[h]**Grape seed procyanidins (w/w):** 89% proanthocyanidins (6.6% dimers, 5.0% trimers, 2.9% tetramers, and 74.8% oligomers).
[i]**Green Tea composition (mg L^{-1}):** 202±11 epigallocatechin, 397±28 EGCG, 52±5 epicatechin, 62±5 epicatechin gallate and 8±2 catechin.
[j]**PMF composition:** Nobiletin, heptamethoxyflavone, tangeretin, 5-hydroxy-6,7,3’,4-tetramethoxyflavone, 5-hydroxy-6,7,8,3’,4’-pentamethoxyflavone, 5-hydroxy-3,6,7,3’,4’-pentamethoxyflavone, 5-hydroxy-3,6,7,8,3’,4’-hexamethoxyflavone, 5-hydroxy-6,7,40-trimethoxyflavone and 5-hydroxy-6,7,8,4’-tetramethoxyflavone.

Acknowledgments

This work was supported by the Ramón y Cajal grant (RyC2021-032111-I) and CNS2022-135253 grant funded by the MCIN/AEI/10.13039/501100011033 and by the "European Union NextGenerationEU/PRTR" program. It was also supported by the grants PID2022-136419OB-I00 and PID2022-136915NA-I00 funded by MCIN/AEI/10.13039/501100011033 and "ERDF A way of making Europe" by the European Union, by the grant 22030/PI/22 funded by the Programa Regional de Fomento de la Investigación Científica y Técnica (Plan de Actuación 2022) de la Fundación Séneca-Agencia de Ciencia y Tecnología de la Región de Murcia, Spain, and by the AGROALNEXT program (MICIU, PRTR-C17.I1, Spain) with funding from the European Union NextGenerationEU (PRTR-C17.I1) and Fundación Séneca (Comunidad Autónoma Región de Murcia, Spain). CS was supported by NIH award R35GM144091 by the National Institute of General Medical Sciences. AV-P was supported by a predoctoral contract associated with the grant PID2022-136915NA-I00 funded by MCIN/AEI/10.13039/501100011033 and "ERDF A way of making Europe" by the European Union.

References

[1] L.-S. Wang, S. S. Hecht, S. G. Carmella, N. Yu, B. Larue, C. Henry, C. McIntyre, C. Rocha, J. F. Lechner, G. D. Stoner, *Cancer Prevent. Res.* **2009**, *2*, 84.

[2] C. Hui, Y. Bin, Y. Xiaoping, Y. Long, C. Chunye, M. Mantian, L. Wenhua, *Nutr. Cancer* **2010**, *62*, 1128.

[3] D. S. Peiffer, L.-S. Wang, N. P. Zimmerman, B. W. S. Ransom, S. G. Carmella, C.-T. Kuo, J.-H. Chen, K. Oshima, Y.-W. Huang, S. S. Hecht, G. D. Stoner, *Cancer Immunol. Res.* **2016**, *4*, 72.

[4] A. C. Silveira Rabelo, S. U. Mertens-Talcott, B. P. Chew, G. Noratto, *Molecules* **2022**, *27*, 7245.

[5] H.-J. Lee, N.-J. Seo, S.-J. Jeong, Y. Park, D.-B. Jung, W. Koh, H.-J. Lee, E.-O. Lee, K. S. Ahn, K. S. Ahn, J. Lü, S.-H. Kim, *Carcinogenesis* **2011**, *32*, 804.

[6] M. Zhao, S.-N. Tang, J. L. Marsh, S. Shankar, R. K. Srivastava, *Cancer Lett.* **2013**, *337*, 210.

[7] J. Kowshik, H. Giri, T. K. K. Kishore, R. Kesavan, R. N. Vankudavath, G. B. Reddy, M. Dixit, S. Nagini, *Anticancer Agents Med. Chem.* **2014**, *14*, 1249.

[8] L. Hu, X. Chen, S. Qiu, J. Yang, H. Liu, J. Zhang, D. Zhang, F. Wang, *Am. J. Chin. Med.* **2020**, *48*, 1005.

[9] F. Bertolini, L. Fusetti, C. Rabascio, S. Cinieri, G. Martinelli, G. Pruneri, *Leukemia* **2000**, *14*, 1477.

[10] V. M. Adhami, I. A. Siddiqui, N. Ahmad, S. Gupta, H. Mukhtar, *Cancer Res.* **2004**, *64*, 8715.

[11] G. Fassina, R. Venè, M. Morini, S. Minghelli, R. Benelli, D. M. Noonan, A. Albini, *Clin. Cancer Res.* **2004**, *10*, 4865.

[12] J. Liao, G.-Y. Yang, E. S. Park, X. Meng, Y. Sun, D. Jia, D. N. Seril, C. S. Yang, *Nutr. Cancer* **2004**, *48*, 44.

[13] M. W. Roomi, V. Ivanov, T. Kalinovsky, A. Niedzwiecki, M. Rath, *Med. Oncol.* **2005**, *22*, 129.

[14] M. W. Roomi, V. Ivanov, T. Kalinovsky, A. Niedzwiecki, M. Rath, *Oncol. Rep.* **2005**, *13*, 421.

[15] M. W. Roomi, V. Ivanov, T. Kalinovsky, A. Niedzwiecki, M. Rath, *In Vivo* **2005**, *19*, 179.

[16] S. K. Mantena, S. M. Meeran, C. A. Elmets, S. K. Katiyar, *J. Nutr.* **2005**, *135*, 2871.

[17] M. W. Roomi, V. Ivanov, T. Kalinovsky, A. Niedzwiecki, M. Rath, *Med. Oncol.* **2006**, *23*, 105.

[18] M. W. Roomi, V. Ivanov, S. Netke, T. Kalinovsky, A. Niedzwiecki, M. Rath, *In Vivo* **2006**, *20*, 25.

[19] M. W. Roomi, V. Ivanov, T. Kalinovsky, A. Niedzwiecki, M. Rath, *Med. Oncol.* **2006**, *23*, 411.

[20] F. Spinella, L. Rosanò, V. Di Castro, S. Decandia, A. Albini, M. R. Nicotra, P. G. Natali, A. Bagnato, *Mol. Cancer Ther.* **2006**, *5*, 1483.

[21] M. R. Sartippour, R. Pietras, D. C. Marquez-Garban, H.-W. Chen, D. Heber, S. M. Henning, G. Sartippour, L. Zhang, M. Lu, O. Weinberg, J. Y. Rao, M. N. Brooks, *Carcinogenesis* **2006**, *27*, 2424.

[22] R. Kumaraguruparan, P. B. Seshagiri, Y. Hara, S. Nagini, *Mol. Carcinog.* **2007**, *46*, 797.

[23] B. D. Lawenda, D. E. Smith, L. Xu, A. Niemierko, J. R. Silverstein, Y. Boucher, S. Kashiwagi, K. D. Held, R. K. Jain, J. S. Loeffler, D. M. Eisenberg, J. B. Blumberg, *J. Soc. Integr. Oncol.* **2007**, *5*, 11.

[24] P. V. Letchoumy, K. V. P. C. Mohan, D. Prathiba, Y. Hara, S. Nagini, *J. Carcinog.* **2007**, *6*, 19.

[25] R. S. Murugan, K. V. P. C. Mohan, K. Uchida, Y. Hara, D. Prathiba, S. Nagini, *J. Gastroenterol.* **2007**, *42*, 352.

[26] H. Leong, P. S. Mathur, G. L. Greene, *Breast Cancer Res. Treat.* **2008**, *107*, 359.

[27] P. V. Letchoumy, K. V. P. C. Mohan, J. J. Stegeman, H. V. Gelboin, Y. Hara, S. Nagini, *Oncol. Res.* **2008**, *17*, 193.

[28] S. Shankar, S. Ganapathy, S. R. Hingorani, R. K. Srivastava, *Front. Biosci.* **2008**, *13*, 440.

[29] M. Sukhthankar, K. Yamaguchi, S.-H. Lee, M. F. McEntee, T. E. Eling, Y. Hara, S. J. Baek, *Gastroenterology* **2008**, *134*, 1972.

[30] W. Wen, J. Lu, K. Zhang, S. Chen, *Cancer Prev. Res. (Phila)* **2008**, *1*, 554.

[31] S. Akhtar, S. M. Meeran, N. Katiyar, S. K. Katiyar, *Clin. Cancer Res.* **2009**, *15*, 821.

[32] R. S. Murugan, G. Vinothini, Y. Hara, S. Nagini, *Anticancer Res.* **2009**, *29*, 2301.

[33] Y. Shirakami, M. Shimizu, S. Adachi, H. Sakai, T. Nakagawa, Y. Yasuda, H. Tsurumi, Y. Hara, H. Moriwaki, *Cancer Sci.* **2009**, *100*, 1957.

[34] X. Fan, D. Mustafi, M. Zamora, J. N. River, S. Foxley, G. S. Karczmar, *Phys. Med.* **2010**, *26*, 111.

[35] Y. Sagara, Y. Miyata, K. Nomata, T. Hayashi, H. Kanetake, *Cancer Epidemiol.* **2010**, *34*, 350.

[36] M. Shimizu, Y. Shirakami, H. Sakai, Y. Yasuda, M. Kubota, S. Adachi, H. Tsurumi, Y. Hara, H. Moriwaki, *Chem. Biol. Interact.* **2010**, *185*, 247.

[37] S. M. Henning, P. Wang, J. Said, C. Magyar, B. Castor, N. Doan, C. Tosity, A. Moro, K. Gao, L. Li, D. Heber, *J. Nutr. Biochem.* **2012**, *23*, 1537.

[38] N. A. H. Sadik, *Cell Biochem. Funct.* **2013**, *31*, 196.

[39] Y. Sakamoto, N. Terashita, T. Muraguchi, T. Fukusato, S. Kubota, *Biosci. Biotechnol. Biochem.* **2013**, *77*, 1799.

[40] J.-W. Gu, K. L. Makey, K. B. Tucker, E. Chinchar, X. Mao, I. Pei, E. Y. Thomas, L. Miele, *Vascular Cell* **2013**, *5*, 9.

[41] S. Shankar, L. Marsh, R. K. Srivastava, *Mol. Cell Biochem.* **2013**, *372*, 83.

[42] L.-L. Feng, B.-X. Liu, J.-Y. Zhong, L.-B. Sun, H.-S. Yu, *Asian Pac. J. Cancer Prev.* **2014**, *15*, 737.

[43] M. W. Roomi, T. Kalinovsky, J. Cha, N. W. Roomi, A. Niedzwiecki, M. Rath, *Exp. The.r Med.* **2015**, *9*, 294.

[44] T. Matsuo, Y. Miyata, A. Asai, Y. Sagara, B. Furusato, J. Fukuoka, H. Sakai, *PLoS One* **2017**, *12*, e0171091.

[45] G. Jin, Y. Yang, K. Liu, J. Zhao, X. Chen, H. Liu, R. Bai, X. Li, Y. Jiang, X. Zhang, J. Lu, Z. Dong, *Oncogenesis* **2017**, *6*, e384.

[46] J. Feng, C. Wang, T. Liu, J. Li, L. Wu, Q. Yu, S. Li, Y. Zhou, J. Zhang, J. Chen, J. Ji, K. Chen, Y. Mao, F. Wang, W. Dai, X. Fan, J. Wu, C. Guo, *J. Cell Mol. Med.* **2019**, *23*, 6479.

[47] G. C. W. Man, J. Wang, Y. Song, J. H. Wong, Y. Zhao, T. S. Lau, K. T. Leung, T. H. Chan, H. Wang, J. Kwong, T. B. Ng, C. C. Wang, *BMC Cancer* **2020**, *20*, 964.

[48] V. K. Nimbalkar, J. Gangar, S. Shai, P. Rane, S. K. Mohanta, S. Kannan, A. Ingle, N. Mittal, S. Rane, M. B. Mahimkar, *Sci. Rep.* **2022**, *12*, 14516.

[49] M. M. Saleh, Z. E. Darwish, M. I. El Nouaem, N. A. Fayed, G. M. Mourad, O. R. Ramadan, *BDJ Open* **2023**, *9*, 30.

[50] L. D. Nagaprashantha, R. Vatsyayan, J. Singhal, P. Lelsani, L. Prokai, S. Awasthi, S. S. Singhal, *Carcinogenesis* **2011**, *32*, 568.

[51] N. Nalini, S. Aranganathan, J. Kabalimurthy, *Toxicol. Mech. Methods* **2012**, *22*, 397.

[52] K. Anand, A. Sarkar, A. Kumar, R. K. Ambasta, P. Kumar, *Nutr. Cancer* **2012**, *64*, 714.

[53] Y. Liu, S. Xie, Y. Wang, K. Luo, Y. Wang, Y. Cai, *Molecules* **2012**, *17*, 7206.

[54] Y. Gong, R. Dong, X. Gao, J. Li, L. Jiang, J. Zheng, S. Cui, M. Ying, B. Yang, J. Cao, Q. He, *Pharmacol. Res.* **2019**, *148*, 104460.

[55] A. A. Khamis, E. M. M. Ali, E. I. Salim, M. A. A. El-Moneim, *Sci. Rep.* **2024**, *14*, 1510.

[56] C. Dabrosin, J. Chen, L. Wang, L. U. Thompson, *Cancer Lett.* **2002**, *185*, 31.

[57] S. Giacomelli, D. Gallo, P. Apollonio, C. Ferlini, M. Distefano, P. Morazzoni, A. Riva, E. Bombardelli, S. Mancuso, G. Scambia, *Life Sci.* **2002**, *70*, 1447.

[58] D. Gallo, S. Giacomelli, C. Ferlini, G. Raspaglio, P. Apollonio, S. Prislei, A. Riva, P. Morazzoni, E. Bombardelli, G. Scambia, *Eur. J. Cancer* **2003**, *39*, 2403.

[59] R. P. Singh, G. Sharma, S. Dhanalakshmi, C. Agarwal, R. Agarwal, *Cancer Epidemiol. Biomarkers Prev.* **2003**, *12*, 933.

[60] R. P. Singh, G. Deep, M. Chittezhath, M. Kaur, L. D. Dwyer-Nield, A. M. Malkinson, R. Agarwal, *J. Natl. Cancer Inst.* **2006**, *98*, 846.

[61] M. Bergman Jungeström, L. U. Thompson, C. Dabrosin, *Clin. Cancer Res.* **2007**, *13*, 1061.

[62] M. Gu, R. P. Singh, S. Dhanalakshmi, C. Agarwal, R. Agarwal, *Cancer Res.* **2007**, *67*, 3483.

[63] R. P. Singh, G. Deep, M.-J. Blouin, M. N. Pollak, R. Agarwal, *Carcinogenesis* **2007**, *28*, 2567.

[64] G. Deep, K. Raina, R. P. Singh, N. H. Oberlies, D. J. Kroll, R. Agarwal, *Int. J. Cancer* **2008**, *123*, 2750.

[65] R. P. Singh, A. Tyagi, G. Sharma, S. Mohan, R. Agarwal, *Clin. Cancer Res.* **2008**, *14*, 300.

[66] N. M. Saarinen, A. Wärri, R. P. M. Dings, M. Airio, A. I. Smeds, S. Mäkelä, *Int. J. Cancer* **2008**, *123*, 1196.

[67] R. P. Singh, K. Raina, G. Sharma, R. Agarwal, *Clin. Cancer Res.* **2008**, *14*, 7773.

[68] R. P. Singh, M. Gu, R. Agarwal, *Cancer Res.* **2008**, *68*, 2043.

[69] K. Raina, S. Rajamanickam, R. P. Singh, G. Deep, M. Chittezhath, R. Agarwal, *Cancer Res.* **2008**, *68*, 6822.

[70] G. Ramakrishnan, S. Jagan, S. Kamaraj, P. Anandakumar, T. Devaki, *Invest. New Drugs* **2009**, *27*, 233.

[71] A. Tyagi, R. P. Singh, K. Ramasamy, K. Raina, E. F. Redente, L. D. Dwyer-Nield, R. A. Radcliffe, A. M. Malkinson, R. Agarwal, *Cancer Prev. Res.* **2009**, *2*, 74.

[72] B. Velmurugan, S. C. Gangar, M. Kaur, A. Tyagi, G. Deep, R. Agarwal, *Pharm. Res.* **2010**, *27*, 2085.

[73] N. M. Saarinen, A. Abrahamsson, C. Dabrosin, *Int. J. Cancer* **2010**, *127*, 737.

[74] J. K. Saggar, J. Chen, P. Corey, L. U. Thompson, *Mol. Nutr. Food Res.* **2010**, *54*, 415.

[75] S. Rajamanickam, B. Velmurugan, M. Kaur, R. P. Singh, R. Agarwal, *Cancer Res.* **2010**, *70*, 2368.

[76] K. Ravichandran, B. Velmurugan, M. Gu, R. P. Singh, R. Agarwal, *Clin. Cancer Res.* **2010**, *16*, 4595.

[77] G. Lindahl, N. Saarinen, A. Abrahamsson, C. Dabrosin, *Cancer Res.* **2011**, *71*, 51.

[78] K. Ramasamy, L. D. Dwyer-Nield, N. J. Serkova, K. M. Hasebroock, A. Tyagi, K. Raina, R. P. Singh, A. M. Malkinson, R. Agarwal, *Clin. Cancer Res.* **2011**, *17*, 753.

[79] J. M. Nagel, M. Brinkoetter, F. Magkos, X. Liu, J. P. Chamberland, S. Shah, J. Zhou, G. Blackburn, C. S. Mantzoros, *Nutrition* **2012**, *28*, 67.

[80] G. Deep, S. C. Gangar, S. Rajamanickam, K. Raina, M. Gu, C. Agarwal, N. H. Oberlies, R. Agarwal, *PLoS One* **2012**, *7*, e34630.

[81] D. Nambiar, V. Prajapati, R. Agarwal, R. P. Singh, *Cancer Lett.* **2013**, *334*, 109.

[82] M. Vaid, T. Singh, R. Prasad, S. K. Katiyar, *Mol. Carcinog.* **2015**, *54*, 1328.

[83] M. Sozmen, A. K. Devrim, R. Tunca, M. Bayezit, S. Dag, D. Essiz, *J. Vet. Sci.* **2014**, *15*, 51.

[84] S. M. Ezzat, S. A. Shouman, A. Elkhoely, Y. M. Attia, M. S. Elsesy, A. S. El Senousy, M. A. Choucry, S. H. El Gayed, A. A. El Sayed, E. A. Sattar, N. El Tanbouly, *Sci. Rep.* **2018**, *8*, 544.

[85] S. Saber, R. Goda, G. S. El-Tanbouly, D. Ezzat, *Int. Immunopharmacol.* **2018**, *64*, 340.

[86] P. Pal, K. Hales, J. Petrik, D. B. Hales, *J. Ovarian Res.* **2019**, *12*, 49.

[87] N. Zein, F. Yassin, S. Makled, S. S. Alotaibi, S. M. Albogami, G. Mostafa-Hedeab, G. E.-S. Batiha, Y. H. A. Elewa, *Biomed. Pharmacother.* **2022**, *150*, 113020.

[88] M. E. Amer, M. A. Amer, A. I. Othman, D. A. Elsayed, M. A. El-Missiry, O. A. Ammar, *Mol Biol. Rep.* **2022**, *49*, 4659.

[89] M. Melaibari, H. M. Alkreathy, A. Esmat, N. A. Rajeh, R. A. Shaik, A. A. Alghamdi, A. Ahmad, *Biomedicines* **2023**, *11*, 1342.

[90] J. R. Zhou, E. T. Gugger, T. Tanaka, Y. Guo, G. L. Blackburn, S. K. Clinton, *J. Nutr.* **1999**, *129*, 1628.

[91] T. Miura, L. Yuan, B. Sun, H. Fujii, M. Yoshida, K. Wakame, K. Kosuna, *Biosci. Biotechnol. Biochem.* **2002**, *66*, 2626.

[92] J.-R. Zhou, L. Yu, Y. Zhong, R. L. Nassr, A. A. Franke, S. M. Gaston, G. L. Blackburn, *Prostate* **2002**, *53*, 143.

[93] J.-R. Zhou, L. Yu, Y. Zhong, G. L. Blackburn, *J. Nutr.* **2003**, *133*, 516.

[94] J.-R. Zhou, L. Yu, Z. Mai, G. L. Blackburn, *Int. J. Cancer* **2004**, *108*, 8.

[95] A. V. Singh, A. A. Franke, G. L. Blackburn, J.-R. Zhou, *Cancer Res.* **2006**, *66*, 1851.

[96] D. Gallo, C. Ferlini, M. Fabrizi, S. Prislei, G. Scambia, *Carcinogenesis* **2006**, *27*, 1404.

[97] Y. Yang, Z. T. Zhou, J. P. Ge, *Carcinogenesis* **2006**, *27*, 578.

[98] S. Mukhopadhyay, B. R. Ballard, S. Mukherjee, S. M. Kabir, S. K. Das, *Mol. Cell Biochem.* **2006**, *290*, 169.

[99] K. Park, K. Choi, H. Kim, K. Kim, M. H. Lee, J. H. Lee, J. C. Kim Rim, *Exp. Mol. Med.* **2009**, *41*, 371.

[100] X. Kang, S. Jin, Q. Zhang, *J. Food Sci.* **2009**, *74*, H237.

[101] H. Li, W. Xu, Y. Huang, X. Huang, L. Xu, Z. Lv, *Int. J. Mol. Med.* **2012**, *30*, 1081.

[102] S. H. Lee, J. Lee, M. H. Jung, Y. M. Lee, *Mol. Nutr. Food Res.* **2013**, *57*, 225.

[103] C. Kaga, A. Takagi, M. Kano, S. Kado, I. Kato, M. Sakai, K. Miyazaki, M. Nanno, F. Ishikawa, Y. Ohashi, M. Toi, *Cancer Sci.* **2013**, *104*, 1508.

[104] A. A. F. Carbonel, M. L. Calió, M. A. Santos, C. R. A. Bertoncini, G. da S. Sasso, R. S. Simões, M. J. Simões, J. M. Soares, *Climacteric* **2015**, *18*, 389.

[105] S.-H. Lee, J.-G. Jee, J.-S. Bae, K.-H. Liu, Y. M. Lee, *J. Cell Physiol.* **2015**, *230*, 853.

[106] X. Xiao, Z. Liu, R. Wang, J. Wang, S. Zhang, X. Cai, K. Wu, R. C. Bergan, L. Xu, D. Fan, *Oncotarget* **2015**, *6*, 3225.

[107] X. Y. Wu, H. Xu, Z. F. Wu, C. Chen, J. Y. Liu, G. N. Wu, X. Q. Yao, F. K. Liu, G. Li, L. Shen, *Oncotarget* **2015**, *6*, 44563.

[108] M. A. Santos, R. Florencio-Silva, C. P. Teixeira, G. R. da S. Sasso, D. S. Marinho, R. S. Simões, M. J. Simões, A. F. Carbonel, *Climacteric* **2016**, *19*, 77.

[109] E. Hejazi, M. Tavakoli, M. Jeddi-Tehrani, M. Kimiagar, J. Hejazi, M. Houshyari, Z. Amiri, H. Edalatkhah, J. Nasrollahzadeh, F. Idali, *Nutr. Cancer* **2017**, *69*, 1036.

[110] M. Hassanshahi, Y.-W. Su, S. Khabbazi, C.-M. Fan, K.-M. Chen, J.-F. Wang, A. Qian, P. R. Howe, D.-W. Yan, H.-D. Zhou, C. J. Xian, *J. Cell Physiol.* **2019**, *234*, 11276.

[111] D. Li, L. Zhao, Y. Li, X. Kang, S. Zhang, *Drug. Des. Devel. Ther.* **2020**, *14*, 2207.

[112] J. Choi, S. B. Won, Y. H. Kwon, *Nutrients* **2020**, *12*, 571.

[113] L. Yi, Y. Lu, S. Yu, Q. Cheng, L. Yi, *J. Recept. Signal Transduct. Res.* **2022**, *42*, 16.

[114] F. Ferriere, F. Percevault, N. Plu, Y. Le Page, T.-H. Pham, S. Lecomte, N. Costet, C. Surel, T. Efstathiou, F. Pakdel, *J. Food Sci.* **2024**, *89*, 59.

[115] C.-S. Lai, M.-L. Tsai, A.-C. Cheng, S. Li, C.-Y. Lo, Y. Wang, H. Xiao, C.-T. Ho, Y.-J. Wang, M.-H. Pan, *Mol. Nutr. Food Res.* **2011**, *55*, 278.

[116] C. Liu, X.-W. Li, L.-M. Cui, L.-C. Li, L.-Y. Chen, X.-W. Zhang, *World J. Gastroenterol.* **2011**, *17*, 4875.

[117] S. Shukla, G. T. MacLennan, P. Fu, S. Gupta, *Pharm. Res.* **2012**, *29*, 1506.

[118] H. Li, H. Fan, Z. Wang, J. Zheng, W. Cao, *PLoS One* **2013**, *8*, e59473.

[119] S. Silvan, S. Manoharan, *Arch. Oral Biol.* **2013**, *58*, 94.

[120] Y. Kimura, M. Sumiyoshi, *Phytomedicine* **2013**, *20*, 328.

[121] S. Shukla, E. Shankar, P. Fu, G. T. MacLennan, S. Gupta, *PLoS One* **2015**, *10*, e0138710.

[122] H. Gao, Q. Jiang, Y. Han, J. Peng, C. Wang, *Cell Biochem. Biophys.* **2015**, *71*, 757.

[123] X. Wu, M. Song, P. Qiu, K. Rakariyatham, F. Li, Z. Gao, X. Cai, M. Wang, F. Xu, J. Zheng, H. Xiao, *Carcinogenesis* **2017**, *38*, 455.

[124] M. Rajasekar, K. Suresh, K. Sivakumar, *Biomed. Pharmacother.* **2016**, *83*, 1064.

[125] B. Lin, K. Zhao, D. Yang, D. Bai, Y. Liao, Y. Zhou, Z. Yu, X. Yu, Q. Guo, N. Lu, *J. Cell Physiol.* **2019**, *234*, 1913.

[126] Z. Zhao, B. Liu, J. Sun, L. Lu, L. Liu, J. Qiu, Q. Li, C. Yan, S. Jiang, N. Mohammadtursun, W. Ma, M. Li, J. Dong, W. Gong, *Int. J. Biol. Sci.* **2019**, *15*, 1500.

[127] Y. Huang, J. Fang, W. Lu, Z. Wang, Q. Wang, Y. Hou, X. Jiang, O. Reizes, J. Lathia, R. Nussinov, C. Eng, F. Cheng, *Cell Chem. Biol.* **2019**, *26*, 1143.

[128] H. Bai, H. Yin, *Biochem. Biophys. Res. Commun.* **2020**, *526*, 497.

[129] L. Lu, M. Zhang, X. Wang, Y. Zhang, Z. Chai, M. Ying, J. Guan, W. Gong, Z. Zhao, L. Liu, Y. Hu, W. Lu, J. Dong, *Phytother. Res.* **2020**, *34*, 104.

[130] Z. Zhang, L. Nong, M. Chen, X. Gu, W. Zhao, M. Liu, W. Cheng, *ABBS* **2020**, *52*, 1007.

[131] A. Naiki-Ito, T. Naiki, H. Kato, K. Iida, T. Etani, Y. Nagayasu, S. Suzuki, Y. Yamashita, S. Inaguma, M. Onishi, Y. Tanaka, T. Yasui, S. Takahashi, *Carcinogenesis* **2020**, *41*, 1145.

[132] Z. Zhao, B. Liu, J. Sun, L. Lu, L. Liu, J. Qiu, Q. Li, C. Yan, S. Jiang, N. Mohammadtursun, W. Ma, M. Li, J. Dong, W. Gong, *Evid. Based Complement. Alternat. Med.* **2019**, *2019*, 9806062.

[133] H. F. Gul, N. Ilhan, N. Ilhan, I. H. Ozercan, T. Kuloglu, *J. Nutr. Biochem.* **2021**, *89*, 108566.

[134] X. Li, Y. Li, Y. Wang, F. Liu, Y. Liu, J. Liang, R. Zhan, Y. Wu, H. Ren, X. Zhang, J. Liu, *Exp. Ther. Med.* **2022**, *23*, 360.

[135] Z. Li, H. Ge, Y. Xie, Y. Zhang, X. Zhao, W. Sun, M. Song, *Tissue Cell* **2023**, *85*, 102235.

[136] X. Gu, R. Zhang, Y. Sun, X. Ai, Y. Wang, Y. Lyu, X. Wang, Y. Wu, Z. Wang, N. Feng, Y. Liu, *J. Nanobiotechnology* **2023**, *21*, 206.

[137] Z.-S. Ma, T. H. Huynh, C. P. Ng, P. T. Do, T. H. Nguyen, H. Huynh, *Int. J. Oncol.* **2004**, *24*, 1297.

[138] R. Kale, M. Saraf, A. Juvekar, P. Tayade, *J. Pharm. Pharmacol.* **2006**, *58*, 1351.

[139] R. V. Priyadarsini, G. Vinothini, R. S. Murugan, P. Manikandan, S. Nagini, *Nutr. Cancer* **2011**, *63*, 218.

[140] A. K. Jain, K. Thanki, S. Jain, *Mol Pharm* **2013**, *10*, 3459.

[141] A. K. Jain, K. Thanki, S. Jain, *Pharm Res* **2014**, *31*, 946.

[142] A. K. Jain, K. Thanki, S. Jain, *Nanomed.: Nanotechnol. Biol. Med.* **2014**, *10*, e959.

[143] H. C. Pal, R. D. Baxter, K. M. Hunt, J. Agarwal, C. A. Elmets, M. Athar, F. Afaq, *Oncotarget* **2015**, *6*, 28296.

[144] Y. Yu, W. Cai, C. Pei, Y. Shao, *Biochem. Biophys.l Res. Commun.* **2015**, *458*, 913.

[145] X. Zhao, Q. Wang, S. Yang, C. Chen, X. Li, J. Liu, Z. Zou, D. Cai, *Eur. J. Pharmacol.* **2016**, *781*, 60.

[146] C. K. Singh, G. Chhabra, M. A. Ndiaye, I. A. Siddiqui, J. E. Panackal, C. A. Mintie, N. Ahmad, *Cancers* **2020**, *12*, 2141.

[147] S. Abdu, N. Juaid, A. Amin, M. Moulay, N. Miled, *Molecules* **2022**, *27*, 8082.

[148] D. de C. da Silva, G. D. C. Orfali, M. G. Santana, J. K. Y. Palma, I. R. de O. Assunção, I. M. Marchesi, A. Y. K. Grizotto, N. P. Martinez, S. Felliti, J. A. Pereira, D. G. Priolli, *Oncotarget* **2022**, *13*, 307.

[149] X. Qi, C. Gao, C. Yin, J. Fan, X. Wu, G. Di, J. Wang, C. Guo, *Toxicol. Appl. Pharmacol.* **2022**, *437*, 115889.

[150] W. Xiong, B. Zheng, D. Liu, M. Pu, S. Zhou, Y. Deng, *Mol. Carcinog.* **2024**, *63*, 2254.

[151] Q. Hao, S. M. Henning, C. E. Magyar, J. Said, J. Zhong, M. B. Rettig, J. V. Vadgama, P. Wang, *Biomolecules* **2024**, *14*, 105.

[152] M. Belleri, D. Ribatti, M. Savio, L. A. Stivala, L. Forti, E. Tanghetti, P. Alessi, D. Coltrini, A. Bugatti, S. Mitola, S. Nicoli, V. Vannini, M. Presta, *Mol. Cancer Ther.* **2008**, *7*, 3761.

[153] T. T. Y. Wang, T. S. Hudson, T.-C. Wang, C. M. Remsberg, N. M. Davies, Y. Takahashi, Y. S. Kim, H. Seifried, B. T. Vinyard, S. N. Perkins, S. D. Hursting, *Carcinogenesis* **2008**, *29*, 2001.

[154] K. B. Harikumar, A. B. Kunnumakkara, G. Sethi, P. Diagaradjane, P. Anand, M. K. Pandey, J. Gelovani, S. Krishnan, S. Guha, B. B. Aggarwal, *Int. J. Cancer* **2010**, *127*, 257.

[155] H. Song, J. I. Jung, H. J. Cho, S. Her, S.-H. Kwon, R. Yu, Y.-H. Kang, K. W. Lee, J. H. Y. Park, *J. Nutr. Biochem.* **2015**, *26*, 1368.

[156] C.-T. Chen, Y.-C. Chen, Y. Du, Z. Han, H. Ying, R. R. Bouchard, J. L. Hsu, J.-M. Hsu, T. M. Mitcham, M.-K. Chen, H.-L. Sun, S.-S. Chang, D. Li, P. Chang, R. A. DePinho, M.-C. Hung, *Am J. Cancer Res.* **2017**, *7*, 657.

[157] W.-H. Hu, G. K.-L. Chan, R. Duan, H.-Y. Wang, X.-P. Kong, T. T.-X. Dong, K. W.-K. Tsim, *Cancers (Basel)* **2019**, *11*, 1828.

[158] J. García-Quiroz, R. García-Becerra, C. Santos-Cuevas, G. J. Ramírez-Nava, G. Morales-Guadarrama, N. Cárdenas-Ochoa, M. Segovia-Mendoza, H. Prado-Garcia, D. Ordaz-Rosado, E. Avila, A. Olmos-Ortiz, S. López-Cisneros, F. Larrea, L. Díaz, *Cancers* **2019**, *11*, 1739.

[159] A. Torres-Hernandez, W. Wang, Y. Nikiforov, K. Tejada, L. Torres, A. Kalabin, Y. Wu, M. I. U. Haq, M. Y. Khan, Z. Zhao, W. Su, J. Camargo, M. Hundeyin, B. Diskin, S. Adam, J. A. K. Rossi, E. Kurz, B. Aykut, S. A. A. Shadaloey, J. Leinwand, G. Miller, *Oncogene* **2019**, *38*, 4512.

[160] R. Pradhan, S. Chatterjee, K. C. Hembram, C. Sethy, M. Mandal, C. N. Kundu, *J. Nutr. Biochem.* **2021**, *92*, 108624.

[161] M. Savio, A. Ferraresi, C. Corpina, S. Vandenberghe, C. Scarlata, V. Sottile, L. Morini, B. Garavaglia, C. Isidoro, L. A. Stivala, *Biomedicines* **2022**, *10*, 1784.

[162] P. Parupathi, G. Campanelli, R. A. Deabel, A. Puaar, L. S. Devarakonda, A. Kumar, A. S. Levenson, *Cancers (Basel)* **2022**, *14*, 6038.

[163] R. Hemani, I. Patel, N. Inamdar, G. Campanelli, V. Donovan, A. Kumar, A. S. Levenson, *Cancer Prev. Res. (Phila)* **2022**, *15*, 87.

[164] R. Pradhan, S. Paul, B. Das, S. Sinha, S. R. Dash, M. Mandal, C. N. Kundu, *J. Nutr. Biochem.* **2023**, *113*, 109257.

[165] R. Pradhan, S. Paul, S. S. Acharya, S. Sinha, S. R. Dash, C. N. Kundu, *J. Nutr. Biochem.* **2024**, *125*, 109568.

[166] D. Kučan, N. Oršolić, D. Odeh, S. Ramić, B. Jakopović, J. Knežević, M. Jazvinšćak Jembrek, *Int. J. Mol. Sci.* **2023**, *24*, 11073.

[167] T. Dorai, Y. C. Cao, B. Dorai, R. Buttyan, A. E. Katz, *Prostate* **2001**, *47*, 293.

[168] Y. G. Lin, A. B. Kunnumakkara, A. Nair, W. M. Merritt, L. Y. Han, G. N. Armaiz-Pena, A. A. Kamat, W. A. Spannuth, D. M. Gershenson, S. K. Lutgendorf, B. B. Aggarwal, A. K. Sood, *Clin. Cancer Res.* **2007**, *13*, 3423.

[169] A. B. Kunnumakkara, S. Guha, S. Krishnan, P. Diagaradjane, J. Gelovani, B. B. Aggarwal, *Cancer Res.* **2007**, *67*, 3853.

[170] A. B. Kunnumakkara, P. Diagaradjane, S. Guha, A. Deorukhkar, S. Shentu, B. B. Aggarwal, S. Krishnan, *Clin. Cancer Res.* **2008**, *14*, 2128.

[171] S. Shankar, S. Ganapathy, Q. Chen, R. K. Srivastava, *Mol. Cancer* **2008**, *7*, 16.

[172] P. Yoysungnoen, P. Wirachwong, C. Changtam, A. Suksamrarn, S. Patumraj, *World J Gastroenterol.* **2008**, *14*, 2003.

[173] C. Chen, X. Huang, H. Cai, J. Xu, *Zhong Nan Da Xue Xue Bao Yi Xue Ban* **2010**, *35*, 1029.

[174] Z.-J. Sun, G. Chen, W. Zhang, X. Hu, Y. Liu, Q. Zhou, L.-X. Zhu, Y.-F. Zhao, *Mol. Pharmacol.* **2011**, *79*, 106.

[175] S. Prakobwong, J. Khoontawad, P. Yongvanit, C. Pairojkul, Y. Hiraku, P. Sithithaworn, P. Pinlaor, B. B. Aggarwal, S. Pinlaor, *Int. J. Cancer* **2011**, *129*, 88.

[176] L. Yan, *Int. J. Cancer* **2013**, *132*, 269.

[177] S. Bimonte, A. Barbieri, G. Palma, A. Luciano, D. Rea, C. Arra, *Biomed. Res. Int.* **2013**, *2013*, 810423.

[178] Q. Yao, Y. Lin, X. Li, X. Shen, J. Wang, C. Tu, *Toxicol. Lett.* **2013**, *222*, 72.

[179] S. Fan, Y. Xu, X. Li, L. Tie, Y. Pan, X. Li, *Biochim. Biophys. Acta* **2014**, *1842*, 1742.

[180] P. Yoysungnoen-Chintana, P. Bhattarakosol, S. Patumraj, *Biomed. Res. Int.* **2014**, *2014*, 817972.

[181] B. Yoysungnoen, P. Bhattarakosol, S. Patumraj, C. Changtam, *Biomed. Res. Int.* **2015**, *2015*, 391748.

[182] M. M. M. Abouzied, H. M. Eltahir, M. A. Abdel Aziz, N. S. Ahmed, A. A. Abd El-Ghany, E. A. Abd El-Aziz, H. O. Abd El-Aziz, *Tumour Biol.* **2015**, *36*, 1763.

[183] H. H. Ahmed, W. G. Shousha, A. B. Shalby, H. A. El-Mezayen, N. N. Ismaiel, N. S. Mahmoud, *Tumour Biol.* **2015**, *36*, 1667.

[184] H. Cao, Y. Wang, X. He, Z. Zhang, Q. Yin, Y. Chen, H. Yu, Y. Huang, L. Chen, M. Xu, W. Gu, Y. Li, *Mol. Pharm.* **2015**, *12*, 922.

[185] A. Hu, J.-J. Huang, R.-L. Li, Z.-Y. Lu, J.-L. Duan, W.-H. Xu, X.-P. Chen, J.-P. Fan, *Sci. Rep.* **2015**, *5*, 13429.

[186] G. G.-L. Yue, H.-F. Kwok, J. K.-M. Lee, L. Jiang, E. C.-W. Wong, S. Gao, H.-L. Wong, L. Li, K.-M. Chan, P.-C. Leung, K.-P. Fung, Z. Zuo, C. B.-S. Lau, *Pharmacol. Res.* **2016**, *111*, 43.

[187] W. Zhang, C. Chen, H. Shi, M. Yang, Y. Liu, P. Ji, H. Chen, R. X. Tan, E. Li, *Phytomedicine* **2016**, *23*, 1.

[188] A. Hu, J.-J. Huang, J.-F. Zhang, W.-J. Dai, R.-L. Li, Z.-Y. Lu, J.-L. Duan, J.-P. Li, X.-P. Chen, J.-P. Fan, W.-H. Xu, H.-L. Zheng, *Oncotarget* **2017**, *8*, 50747.

[189] R. S. Rajmani, P. Singh, L. V. Singh, *Nutr. Cancer* **2017**, *69*, 1245.

[190] X. Xu, Y. Zhu, *Am. J. Transl. Res.* **2017**, *9*, 3633.

[191] S. Zhang, D. Tang, W. Zang, G. Yin, J. Dai, Y. U. Sun, Z. Yang, R. M. Hoffman, X. Guo, *Anticancer Res.* **2017**, *37*, 465.

[192] Z. Pan, J. Zhuang, C. Ji, Z. Cai, W. Liao, Z. Huang, *Oncol. Lett.* **2018**, *15*, 4821.

[193] D. Tang, S. Zhang, X. Shi, J. Wu, G. Yin, X. Tan, F. Liu, X. Wu, X. Du, *Integr. Cancer Ther.* **2019**, *18*, 1534735418824408.

[194] Z. Deng, X.-Y. Xu, F. Yunita, Q. Zhou, Y.-R. Wu, Y.-X. Hu, Z.-Q. Wang, X.-F. Tian, *World J. Gastrointest. Oncol.* **2020**, *12*, 1091.

[195] S. Tian, L. Liao, Q. Zhou, X. Huang, P. Zheng, Y. Guo, T. Deng, X. Tian, *Oncol. Lett.* **2021**, *21*, 286.

[196] D. Nayak, S. Paul, C. Das, S. Bhal, C. N. Kundu, *J. Cell Commun. Signal.* **2023**, *17*, 609.

[197] S. Bimonte, A. Barbieri, G. Palma, D. Rea, A. Luciano, M. D'Aiuto, C. Arra, F. Izzo, *BioMed Res. Int.* **2015**, *2015*, 878134.

[198] Q. Xiao, B. Deng, A. Akbari, Q. Liu, B. Zhu, *J. Food Biochem.* **2022**, *46*, e14407.

[199] O. F. Onifade, O. A. Akinloye, O. A. Dosumu, A. L. A. Shotuyo, *Food Chem. Toxicol.* **2023**, *182*, 114096.
