# Supplementary material for: Angiogenesis as a Therapeutic Target of (Poly)phenols: Tackling Cancer and Vascular‐Related Complications
Source: Mol Nutr Food Res. 2025 May 15;69(15):e70110. doi: 10.1002/mnfr.70110 (PMC12319488; doi:10.1002/mnfr.70110)
Supplement: Supplementary file 2 — Supporting information [file MNFR-69-e70110-s002.pdf]

Angiogenesis as a therapeutic target of (poly)phenols: tackling cancer and vascular-related complications

María Ángeles Ávila-Gálvez[1,]*, Antonio Vico-Padilla[1], Claus Schneider[2], Juan Carlos Espín[1], Antonio González-Sarrías[1], Juan Antonio Giménez-Bastida[1,]*

[1]Laboratory of Food and Health, Research Group on Quality, Safety and Bioactivity of Plant Foods, Department of Food Science and Technology, CEBAS-CSIC, P.O. Box 164, 30100 Campus de Espinardo, Murcia, Spain

[2]Division of Clinical Pharmacology, Department of Pharmacology, and Vanderbilt Institute of Chemical Biology, Vanderbilt University Medical School, Nashville, Tennessee 37232, United States

Corresponding authors: Juan Antonio Giménez-Bastida[1,]*, PhD (jgbastida@cebas.csic.es) and *María de los Ángeles Ávila-Gálvez[1,]*, PhD (mavila@cebas.csic.es); Telf. (+34) 968396200 Fax: (+34) 968396213

Table S2. Animal studies describing the effect of dietary phenolics-rich extracts or individual compounds

Animal model (related disease)	Extract/phenolic compound	Dose; duration	Main outcomes	Effect on angiogenesis	Reference
			**Anthocyanins**		
♂ Normotensive Wistar rats underwent ligature on the left femoral artery (ischemic peripheral vascularization model)	Delphinidin or RWPC (Provinols^{TM})[a]	RWPC: 0.2, 2 and 20 mg kg^{-1} day^{-1} (gavage); 3 weeks<br><br>Delphinidin: 0.06 and 0.6 mg kg^{-1} day^{-1} (gavage); 15 days	Effect of RWPC:<br>↓Body weight and blood pressure (at the highest dose of RWPC); no effect on body weight at medium and low dose RWPC; ↓blood flow at the highest dose, whereas ↓blood flow at the lowest dose (no effect at the medium dose); t-eNOS: ↑ischemic and non-ischemic tissue and aorta, whereas ↓at high dose in non-ischemic tissue;<br>p-eNOS: ↑ischemic and non-ischemic tissue and aorta<br>p-eNOS/t-eNOS: ↑aorta and non-ischemic tissue:<br>HSP90: ↑ischemic and non-ischemic tissue and aorta, whereas	Effect of RWPC:<br>↓Vascular density at the highest dose, whereas ↑vascular density at the lowest dose (no effect at the medium dose); ↓capillary density (at the highest dose); ↓arteriolar density at the highest dose, whereas ↑arteriolar density at the lowest dose (no effect at the medium dose); ↑VEGF in ischemic tissue (no effect in aorta and non-ischemic tissue); ↓MMP-2 activity at high dose; pro-angiogenic effect of RWPC in the presence of anti-VEGF antibody<br><br>Effect of delphinidin:<br>↓Vascular density (at the highest dose)	Baron-Menguy et al., 2007[1]

			↓at low dose in aorta;<br>caveolin-1: ↑non-ischemic tissue;<br>t-Akt: ↑ischemic and non-ischemic tissue and aorta;<br>p-Akt: ↑ischemic tissue and aorta;<br>PI3K: ↑ischemic and non-ischemic tissue and aorta;<br>p-p38: ↑non-ischemic tissue and aorta;<br>t-p38: ↑non-ischemic tissue, whereas ↓aorta;<br>NF-κB: ↑ischemic, whereas in non-ischemic tissue ↑at low dose and ↓at high dose<br><br><u>Effect of delphinidin:</u><br>↓Blood flow (at the highest dose)		
Apo-E deficient mice (8 weeks) (atherosclerosis model)	Bilberry anthocyanin-rich extract (Antho 50® supplied by Ferlux Mediolanum SA, Cournon d'Auvergne, France) containing 52%	0.02% (w/w) diet; 2 weeks	No effect on body weight; no effect on TG, HDL/LDL ratio and ORAC; ↓TCh	Modulation of gene expression related to VEGF and TGF-β signaling pathway (microarray analysis)	Mauray et al., 2012[2]

	of pure anthocyanins (total (poly)phenols content equivalent to 0.62 gr gallic acid g^{-1} extract)				
♂ db/db mice (C57BLKS/+$Lepr^{db}$ lar) and the non-diabetic db/m littermates C57BLKS/J) (8 weeks) (diabetes model)	Anthocyanin-rich purple corn extract	10 mg kg^{-1} in drinking water; 8 weeks	↓Cell proliferation (Ki-67)	↓Plasma VEGF and TSP-1 level; ↓VEGF, VE-cadherin and HIF-1α in kidney (protein); ↓VEGFR2 and p-VEGFR2; ↓Ang1, Ang2, Tie-2 and p-Tie-2	Kang et al., 2013[3]
♂ Sprague Dawley rats (8 weeks) treated with MCT (Hypertension model)	Cyanidin 3-*O*-β-glucoside	200 and 400 mg kg^{-1} b.w. (gavage); 4 weeks	Improvement of hemodynamic parameters (mPAP, RVSP AND RVHI); ↓medial wall thickness (only at the highest concentration) and area; ↑pH and partial pressure PaO_2 and $PaCO_2$ (only at the highest concentration); ↓partial pressure $PaCO_2$; ↓*Il-6*, *Tnf-α* and ↑*Il-10* (mRNA); ↓IL-6, ↑IL-10 and no significant effect on TNF-α (plasma); ↑SOD activity and ↓MDA level (at the highest concentration); ↓Bcl-2	↑vWf, ICAM-1 and VCAM-1 level (mRNA and protein); ↓VEGF and PDGF-BB (only the highest concentration), SM22, TGF-β1, and α-SMA	Ouyang et al., 2021[4]

			and ↑Bax; ↓p-CREB/t-CREB (only highest concentration) and p-P38/t-P38		
C57BL/6 mice treated with STZ (diabetic retinopathy model)	Cyanidin 3-*O*-β-glucoside	20 mg kg^{-1} b.w. (gavage); 1 and 2 months	↓TNF-α, IL-1β and IL-6 (mRNA and plasma); ↓p-p65/t-p65 and p-IκB/t-IκB; ↓Iba-1; ↑Occludin, ZO-1 and Claudin-1 (mRNA and protein)	↓Vessel leakage; ↓*Vegf*, *Vegfr1*, *Cd31* and *Vegfr2* (mRNA); ↓VEGF and CD31 level (protein and immunofluorescence staining)	Zhao et al., 2021[5]
♂ Wistar rats (7 – 8 weeks) fed anthocyanins (systemic circulation model)	Cyanidin 3-*O*-glucoside	1 mg kg^{-1} day^{-1} (p.o.); single oral dose or 2 weeks	↑Cremasteric blood flow, ↑p-Akt/t-Akt, and no effect on eNOS phosphorylation (single dose); ↓MBP and ↑eNOS level and myofiber size; ↑cross-section myofiber area (rat soleus muscle)	↑CD31 and CD31/myofiber ratio	Fushimi et al., 2023[6]
			**Ellagitannins, ellagic acid, and derived metabolites**		
C57BL/6J stimulated with pellets containing 50 ng bFGF (corneal angiogenesis model)	PGG	20 mg kg^{-1} day^{-1} (gavage); n.d.		↓Blood vessel area	Cryan et al., 2013[7]
♂ Wistar-NIN rats (3 months) injected with STZ (diabetes model)	EA	0.2 and 2% (w/w) administered in an enriched diet; 12 weeks	No influence on food intake and body weight; ↓Blood glucose (non-significant), CML, RAGE, Bax, and HbA_{1c}	↓GFAP, VEGF, and HIF-1α (mRNA and protein)	Raghu et al., 2017[8]

			level (dose-dependent effect); improvement of oscillatory potentials (observed in electroretinograms)		
♂ Athymic BALB/c mice injected with MiaPaCa2 cells and STZ (diabetes/cancer model)	PGG	20 μg g^{-1} (gavage); 5 times per week for 8 weeks	↓Living cell area in tumour sections; ↑Necrotic area; ↓p-IR, t-Akt, p-Akt, p-MEK and p-ERK; No effect on tumor weight or GLUT-1	↓HIF-1α, VEGF, HK-II and PFK-1; ↓p-IGF1R, t-IFG1R	Hu et al., 2020[9]
♂ DBA/1J (7 – 8 weeks) injected with complete Freund-s adjuvant: type II collagen (1:1)	Chebulinic acid	50 mg kg^{-1} day^{-1} (p.o.); 2 weeks	No effect on animal weight, blood pressure, neutropenia and thrombocytopenia, liver enzymes and creatinine level and blood urea nitrogen; ↓CIA severity (gross paw swelling, mean paw diameter, and mean articular index)	↓VEGFR2 phosphorylation, ESM-1 and Apelin	Lu et al., 2020[10]
♂ C57BL/6 (12 weeks) (muscular angiogenesis model)	Uro-A	10 mg kg^{-1} day^{-1} (i.g.); 16 weeks	No toxic effects; ↓P16, ATM and 8-OH-dG (muscle aging markers); ↑ATP, NAD^+ and NAD^+/NADH	Up-regulation of angiogenic pathways (transcriptome profiling analysis); ↑*Gata6*, *Hgf*, *Nrp1*, *Dab2*, *Cyr61*, *Vegfa*, *vWf*, *Vegfr2*, *Pecam1*, *Gata2*, *CD105*, *Tnc* (mRNA)	Gosh et al., 2020[11]

Sprague Dawley rats	Uro-A	25 mg kg^{-1} day^{-1} (i.g.); 3 weeks	↑Wound healing; ↑collagen deposition (type I and III) and cytokeratin expression	↑Formation of capillaries (H&E staining and CD31)	Feng et al., 2022[12]
			**Dihydrochalcone**		
♂ Sprague Dawley rats treated with MCAO (cerebral artery occlusion)	Trilobatin (alone or in combination with MCAO)	20 mg kg^{-1} (gavage); 1, 3, 7, 14 and 28 days	Long-term improvement of neurological functions; ↑CDK4, cyclin D1, and SIRT7 (at 3, 7, 14, and 28 days)	↑Number and functional cerebral microvessels (at 7, 14, and 28 days); ↑VEGFA and VEGFR2 (at 3, 7, 14, and 28 days)	Huang et al., 2021[13]
			**Flavan-3-ols/proanthocyanidins**		
Mice implanted with VEGF pellets (angiogenesis model)	Green tea	4.69 mg mL^{-1} (1.25% w/v) of green tea diluted in drinking water (containing 708 μg mL^{-1} EGCG)		↓VEGF-induced corneal neovascularization measured by blood vessel length (55% reduction) as well as clock hours (35% reduction) and area of neovascularization (70% reduction)	Cao & Cao et al., 1999[14]
♂ New Zealand white rabbits fed HCD	Green tea brew	2.5% (w/v) in drinking water; 17 weeks	↓Atherosclerotic plaque surface	↓Foam cells and SMC positively stained for VEGF in atherosclerotic lesions	Kavantzas et al., 2006[15]
♂ C57BL/6N (1 year-old) underwent treadmill test	(-)-Epicatechin (alone or combined with exercise)	1 mg kg^{-1} (gavage); twice a day for 15 days	Level of (-)-epicatechin reached in plasma 0.67 ± 0.38 mmoL L^{-1}mM (in samples pretreated with β-glucuronidase and sulphatase); ↑p-PI3K/-t-PI3K, p-PDK/t-PDK, p-Akt/t-Akt, p-Src/t-Src	↑Capillary density and CD31 level; ↑HIF-1α, VEGF, p-VEGFR2/t-VEGFR2, ↑eNOS and p-eNOS (Ser^{617} and Ser^{1177}) level and activity; ↑NO_2^- and NO_3^- and cGMP; ↑MMP-2 and MMP-9 level and activity	Ramírez-Sánchez et al., 2012[16]

BALB/c mice (8 weeks) underwent miles assay, and Sprague Dawley rats (7 weeks) underwent RVP (VEGF injected in both assays)	EGCG	200 mg kg^{-1} (p.o.); single dose for 1.5 h (miles assay) or fed for 4 days (RVP assay)		↓Vascular permeability and vascular leakage in the skin; ↓Vascular permeability in the blood-retinal barrier of the eyeball	Sung-Lee et al., 2014[17]
Sprague Dawley rats (5 weeks) treated with ouabain (hypertension model)	GSPE	250 mg kg^{-1} day^{-1}; 5 and 8 weeks	No effect on SBP; ↓profilin-1 and ↑eNOS and NO level (mRNA and protein); improvement of cellular morphology and function; and p-Akt level; no effect on t-Akt and HIF-1β	↑VEGF level (mRNA and protein); ↓HIF-1α level	Hao et al., 2018[18]
♂ Athymic BALB/c mice injected with STZ and MiaPaCa2 cells (diabetes/cancer model)	EGCG	50 μg g^{-1} (gavage); 5 times per week for 8 weeks	↓Total section and living cell area in tumor sections; ↓Tumour weight; ↓p-IR, t-IR, p-IGF1R, t-IFG1R, t-Akt, p-Akt, p-MEK, t-MEK, t-ERK, and p-ERK; No effect on GLUT-1	↓HIF-1α, VEGF, HK-II AND PFK-1	Hu et al., 2020[9]
			**Flavanones**		
Wistar albino rats treated with STZ (diabetes model)	Hesperetin	200 mg kg^{-1} (p.o.); 24 weeks	No effect on body weight; ↓Blood glucose level, HbA_{1c}	↓VEGF and PKC-β; ↓Blood vessels dilation and prevention in capillary basement dilation; Improvement in the angiograms clinical grading	Kumar et al., 2012[19]

♂ Sprague Dawley rats treated with STZ (diabetic model)	Hesperidin	100 and 200 mg kg^{-1} b.w. (p.o.); 2 and 10 weeks	↑retinal thickness and SOD activity; ↓Blood glucose, aldose reductase activity, MDA, TNF-α, ICAM-1, IL-1β, and AGE level	↓Blood-retina breakdown and VEGF level	Shi et al., 2012[20]
♂ Sprague Dawley rats treated with STZ (diabetic model)	Naringin	20, 40 and 40 mg kg^{-1} (p.o.); 16 days	↓Food intake; ↓Glucose, glycated Hb, MDA, and MPO level, creatinine; ↑Body weight and wound healing capacity; ↑SOD, GSH, and hydroxyproline level; Improvement of the lipid profile	↑*Ang-1*, *IGF-1*, *TGF-β*, *VEGF-c* (mRNA); ↑number of blood vessels and mesenchymal matrix deposition along with complete re-epithelialization, well-formed granulation tissue, mature collagen bundles, and structured dermal layers (only at the highest concentration of naringin)	Kandhare et al., 2014[21]
♂ Sprague Dawley rats (flap animal model)	Naringin	40 and 80 mg kg^{-1} (p.o.); 7 days	↑Flap survival area and SOD activity; ↓Edema, vascular dilation and infiltration of inflammatory cells; ↓MDA, TNF-α, and IL-6 level	↑Microvascular density and blood flow; ↑VEGF level	Cheng et al., 2017[22]
♂ C57Bl/6 (6 weeks) fed a high-fat diet (diabetic model)	XN and 8-PN	10 mg L^{-1} administered in an enriched diet; 20 weeks	↓Weight gain, glycemia, and insulin level	↓CD31 level in the kidneys and ↑in the left ventricle; ↑Plasma and left ventricle VEGF-A level (only 8PN); ↓Kidney VEGF-A level (both compounds); ↓Plasma, kidney and left ventricle VEGF-B level	Costa et al., 2017[23]

				(both compounds); ↓VEGFR2, ERK and Akt phosphorylation (only XN) in kidney; ↑VEGFR2 Akt phosphorylation in left ventricle (both compounds); ↓ERK phosphorylation (only 8PN) in kidney; ↓VEGFR1 and NP-1 in kidney and left ventricle; ↓PFKFB3 in kidney and ↑ in left ventricle	
♂ Sprague Dawley rats treated with L-NAME (hypertension model)	Hesperidin	15 and 30 mg kg^{-1}; once daily for 5 weeks (p.o.)	Hypertension prevention; attenuation of wall thickness, cross-sectional area, and fibrosis; ↓Oxidative stress and MDA formation; ↓TNF-α, and TNF-R1	↓TGF-β1, MMP-2 and MMP-9; ↑Plasma NO	Maneesai et al., 2018[24]
♂ Sprague Dawley rats (8 – 10 weeks); skin irradiation using cobalt-60 radiotherapy (skin lesion model)	Hesperidin	100 mg kg^{-1} b.w.; single oral administration 30 min before irradiation		↑*Vegfa* gene expression	Haddadi et al., 2018[25]
♂ Sprague Dawley rats injected with STZ (diabetes model)	Hesperidin	25, 50 and 100 mg kg^{-1} (p.o.); 21 days	Attenuation of diabetic symptoms (body weight loss, low insulin level, high glucose level, and increased water and food intake); Wound	↑*Vegf*, *Ang-1*, *Tie2*, *Tgf-β*, *Smad-2/3* at the mRNA level (only significant at 50 and 100 mg kg^{-1}); ↑blood vessel number and re-epithelialization and	Li et al., 2018[26]

			healing improvement through ↓wound area and ↑wound contraction (only significant at 50 and 100 mg kg^{-1}); ↑SOD, GSH, and hydroxyproline, whereas ↓MDA and NO (only significant at 50 and 100 mg kg^{-1})	↓polymorphonuclear leukocyte infiltration	
♀ Sprague Dawley rats (endometriosis model)	Naringenin	50 mg kg^{-1} body w.t./day; 21 days	↓Weight and volume of endometrial lesions, as well as size and number of glands; No effect on body weight	↓TNF-α and NO serum level	Kapoor et al. 2019[27]
♂ Sprague Dawley rats (6 weeks) treated with STZ (diabetic model)	Hesperetin	100 mg kg^{-1} (p.o.) once a day for 1 week	↑Wound healing rate, GPX4 and SIRT3 level; ↓ACSL4 accumulation	↑Blood flow intensity	Yu et al., 2024[28]
			**Isoflavones**		
♂ Wistar rats treated with STZ (diabetes model)	Biochanin A	10 and 15 mg kg^{-1} b.w./day; 6 weeks	↓Blood glucose and retina TNF-α and IL-1β level; protection of the retina tissues	↓Retina VEGF level	Eskandary et al., 2018[29]
			**Lignans/Flavonolignans**		
♂ Sprague-Dawley rats with induced myocardial infarction	SDG	20 mg kg^{-1} day^{-1}; 2 weeks	↑LVDP, aortic flow and no effect on heart rate or coronary flow (30 min ischemia followed by	↑Capillary density (CD31); ↑VEGF, Ang-1, and p-eNOS	Penumathsa et al., 2006[30]

			reperfusion model); ↓infarct size and cardiomyocytes apoptosis; ↑ejection fraction, LVPW and fraction shortening; ↓LVID and E/A ratio; no effect on LVAW (myocardial infarction model)		
Brown Norway rats subjected to intravitreal VEGF injection (AMD model)	Silibinin	500 mg kg^{-1}; 21 days	↓Retinal edema	↓Retinal permeability and microvascular angiogenesis induced by VEGF treatment and hypoxia	Lin et al., 2013[31]
Offspring of ♀Wistar rats injected with STZ (diabetes model)	FS oil or FS flour	HFD + 70 g kg^{-1} FS oil or HFD + FS flour (25% w/w) during pregnancy and lactation; offspring of diabetic rats sacrificed at 100 days	No significant effect on MCP-1, TCh, TG, HDL, LDL, VLDL; ↓Intima-media layer thickness of the aorta; no effect on the aortic lumen area; ↑elastin	No significant effect on VEGF	Vicente et al., 2015[32]
♂Wistar rats with pressure overload-induced myocardial hypertrophy (aortic stenosis model)	FLC	FLC (400 mg kg^{-1}) (alone or together with ω-3 fatty acids); 4 weeks	↓Heart weight/b.w., LV weight/b.w. and lung weight/b.w.; ↑survival rate (synergic effect); amelioration of LV dysfunction; ↓cardiomyocyte apoptosis and oxidative stress;	↑*Vegfa* (mRNA); Improved myocardial angiogenesis	Ghule et al., 2015[33]

			↓MABP, SBP and DBP (synergic effect); ↓systolic and diastolic duration; improvement of contractibility index, exponential tau, and pressure time index; ↓MDA, MPO, TCh, TG, and VLDL; ↑GSH, SOD, GPx, GST, membrane-bound phosphatase enzymes; ↓serum CKMB, LDH, and TNF-α; ↓myocardial damage, inflammation extent and nuclear pyknosis; improvement of myocardial architecture		
♀ Wistar rats underwent transplantation of uterine horns to the intestinal mesenteric area (endometriosis model)	Silymarin	50 mg kg^{-1}; 28 days	↓Number of endometrial like-legions developed and size; ↑collagen bundles and apoptosis; ↓$GDNF^{+}$ and gfrα1^{+} cells (protein and mRNA level); ↓Bcl-2 and Bcl-6b level (protein and mRNA	↓Tissue vascular distribution area	Nahari et al., 2018[34]

			level); ↑ERK1/2 expression (protein and mRNA level)		
♀ Swiss albino mice (6 weeks) injected with STZ (diabetes model)	Silymarin	10.41 mg kg^{-1} or 104.1 mg kg^{-1}; 10 days	Effects on diabetic animals:<br><br>↓Glycemia; no effect on MPO, collagen, Hb, nitrite, N-AGM; ↑SOD and no effect on LPO in pancreas; ↓TNF-α and no effect on amylase (pancreas); no effect on GST and GSH (pancreas); ↑CAT, NADH and succinate oxidase activity (liver); no effect on MPO, AST, GST or SOD (liver); ↑CAT and ↓MPO in kidney; no effect on GSH, LPO, creatinine or nephrossomatic index (kidney); no effect on plasma urea level<br><br>Effects in non-diabetic animals:<br><br>↑CAT (kidney and liver) and SOD (liver)	No effect on vessel number and vascular area	Stolf et al., 2018[35]

Pigmented rabbits with visible-light-induced retinal damage	FS oil	233 mg kg^{-1} day^{-1}; 3 weeks (2 weeks pre-illumination and 1-week post-illumination)	↑C18:3 ω-3 in plasma; Protection of outer nuclear layer and against retinal damage; ↓Bx/Bcl-2 ratio and caspase-9 activation; ↑retinal CAT, SOD, *Nrf2* and *Hmox-1* (mRNA); no significant effect on *RelA*, *Il-1β*, *Tnf-α* and *Il-8* (mRNA); ↓*Ptgs2* (mRNA)	No significant effect on VEGF and HIF-1α level	Deng et al., 2018[36]
**Flavones**					
♂ Sprague Dawley rats subjected to ischemia/reperfusion (neuronal damage model)	Scutellarin	25, 50, 75 mg kg^{-1} (i.g.); 1 week	↓Infarct area; improvement of neurological deficit; ↓BBB permeability and NOx production	↓eNOS, iNOS, and nNOS in hippocampus and cortex tissues; ↓VEGF and bFGF level	Hu et al., 2005[37]
♂ C57BL/6 mice fed HFD (diabetes/obesity model)	Luteolin	0.002 and 0.01% (w/w) enriched diet; 12 weeks	↓Body and adipose tissue weight; ↓adipocyte diameter; ↓insulin, leptin, adiponectin, TNF-α, MCP-1; ↓IL-6 (no significant); improvement of glucose intolerance and insulin sensitivity; ↑p-Akt/t-Akt and pm-GLUT4/GLUT4;	↓$CD31^+$ area in adipose tissue	Xu et al., 2014[38]

			↓Cathepsin S, K, B and L (mRNA and protein); ↑apoptosis; ↓macrophage and mast cells recruitment; ↓*F4/80*, *Mcp-1*, *TNF-α*, *IL-6* (mRNA) in adipose tissue; ↓*mMcp-5* and *mMcp-6* (mRNA)		
♂ Sprague Dawley rats fed HFD and treated with STZ (diabetes model)	Scutellarin	40 mg kg^{-1} day^{-1} (p.o.); 8 weeks	↓Resistivity index and ↑blood flow velocity	↓VEGF, VEGFR2 and vWf level	Wang et al., 2017[39]
♂ Sprague Dawley rats (model of cerebral ischemia)	Wogonin	50 μmol L^{-1} (10 mg kg^{-1} day^{-1} for 10 days every 24 h) for 2 weeks	↑Recovery of nerve function; ↓apoptosis and ↓edema	↑Angiogenesis after cerebral ischemia; ↑TGF-β1 protein level	Kong et al., 2019[40]
♂ Sprague Dawley rats (8 weeks) with abdominal aorta exposed to $CaCl_2$ (aneurysm model)	Apigenin	40 and 80 mg kg^{-1} (p.o.); 3 months	↓Aortic diameter and expansion; no effect on body weight; ↓NF-κB activation; protection of SMC and ↑SM-22α and α-SMA	↓Elastin degradation; ↓MMP-2 and -9 (mRNA and protein)	Li et al., 2019[41]
♂ Sprague Dawley rats fed HFD and treated with STZ (diabetic model)	Scutellarin	40 mg kg^{-1} day^{-1} (p.o.); 8 weeks	No effect on blood glucose level or body weight loss; no effect on the retinal vessel blood flow velocity; attenuation of the resistive index (resistance of blood	↓Number of neovascular nuclei; no effect on PDGF; ↓VEGF, p-ERK/t-ERK, p-FAK/t-FAK, p-Src/t-Src	Long et al., 2019[42]

			flow) of the retinal vessels		
♂ Sprague Dawley rats subjected to the McFarlane flap technique (skin model)	Apigenin	20 and 50 mg kg^{-1} b.w. (p.o.) per day; 1 week	↓Necrosis and tissue edema (dose-dependent); protection of the skin architecture; ↓neutrophil infiltration (dose-dependent); ↑SOD activity and ↓MDA level	↑Microvessel density and ↑blood perfusion (dose-dependent); ↑neovascularization; ↑ VEGF and ↓IL-1β (tissue); ↓IL-6, and TNF-α (serum and tissue)	Ma et al., 2021[43]
C57BL/6 mice (8 weeks) ovariectomized (osteoporosis model)	Acacetin	20 mg kg^{-1} day^{-1} (p.o.); 8 weeks	No toxicity in the liver or kidney; ↓bone loss; ↑bone area and ↓fat accumulation in the bone marrow; ↑number of osteoblasts on the surface of the trabecular bone; ↑osteoclasts on the trabecular bone; ↓osteoclastogenic activity; ↑*Acp5* and *Rankl/Opg*	↑$CD31^{hi}EMNC^{hi}$ vessels and PDGF-BB^{+}/$TRAP^{+}$ cells	Lin et al., 2022[44]
♂ Sprague Dawley rats treated with MCT (pulmonary hypertension model)	Wogonin	20 mg kg^{-1} day^{-1} (gavage); 3 weeks	↓RVSP and RVHI, the thickness of the pulmonary vascular wall, PCNA-positive cells, phosphorylated	↓αSMA positive cells and ↑CD31 (modulation of endothelial to mesenchymal transition); ↓TGF-β1 level	Wang et al., 2024[45]

			SMAD1/5, SMAD2, and snail levels<br><br>Amelioration of the lung/body weight ratio and no effect on the liver/body weight ratio	Wogonin shows affinity to TGF-β1 receptor (in silico assay)	
			**Flavonol**		
♂ Albino rats treated with titanium dioxide nanoparticles (renal damage model)	Quer	200 mg kg^{-1} (p.o.); 3 weeks	↓Serum kidney function biomarkers (urea, creatinine, and uric acid); ↓blood glucose and CRP; ↓serum IgG and ↑GSH; improvement of histomorphological kidney features	↓TNF-α, IL-6, VEGF and NO	Al-rasheed et al., 2013[46]
♂ ApoE knockout mice (C57BL/6 background) (4 weeks) fed HFD and treated with STZ	Rutin	60 mg kg^{-1} day^{-1} (0.1 mL in distilled water); 6 weeks	↓TCh, TG, LDL and fasting blood glucose; amelioration of glucose intolerance and insulin resistance; improvement of ventricular remodeling; alleviation of HDF-induced systolic and diastolic dysfunction; ↓cardiac fibrosis and collagen accumulation; ↓apoptosis and	↓TGF-β and ↑capillary density (Isolectin IB4 and CD31)	Huang et al., 2017[47]

			cleaved caspase-3; ↓lipid deposition in cardiac myocytes; ↓ROS production, ↑SOD, GSH and GSH/GSSG ratio; ↓GSSG, MDA, total non-heme iron and Nox4; ↑p-Akt/t-Akt, p-JNK/t-JNK, p-ERK/t-ERK, p-P38/t-P38		
♀ Sprague Dawley rats (8 weeks) fed HFD (diabetes model)	Quer	20 mg kg^{-1} b.w. in diet; 6 weeks	No significant effect on body weight or biochemical parameters; ↓plasma TNF-α; amelioration of glucose intolerance and insulin resistance; ↓adipocyte size and ↑BAT and ↓WAT/BAT ratio; ↑PPAR-γ, C/EPBα, adiponectin and FAS; ↓TLR-4, CD68, MCP-1, p-JNK (Thr183/Tyr185), ATF-6 and XBP-1	↓HIF-1α and ↑VEGF-A and VEGFR2	Perdicaro et al., 2020[48]
♂ C57BL/6 mice (5 – 6 weeks) subjected to aortic application of CaCl2 (aortic aneurism model)	Quer	60 mg kg^{-1} (p.o.); 6 weeks	↓Elastin proteolysis and loss of collagen; ↓PGE_2	↓Microvessel density (CD31); ↓VEGF-A and HIF-1α (mRNA and protein); ↓*ICAM-1*, *VCAM-1*, *COX-2* and	Wang et al., 2020[49]

				VE-cadherin (mRNA); no effect on *TGF-1* and *FGF* (mRNA)	
♂ Sprague Dawley rats (8 weeks) treated with STZ (diabetes model)	Quer (alone or in combination with Zn protoporphyrin)	Quer: 150 mg kg^{-1} (i.g.)<br><br>Quer + Zn protoporphyrin: 150 mg kg^{-1} (i.g.) + 30 mg kg^{-1} (i.p.); 16 weeks	No effect on blood glucose level; ↑Retinal layer thickness and number of ganglion cells; ↑retinal HO-1; ↓HMGB1, ASC, caspase-1 and NLRP3 activation (mRNA and protein); ↓retinal IL-1β, IL-18, IL-6 and TNF-α level; ↓TLF-4 and NF-κBp65; ↓apoptosis; ↑BDNF and NGF; effects prevented in combination with Zn protoporphyrin (HO-1 inhibitor)	↓VEGF and sICAM-1	Chai et al., 2021[50]
♂ Sprague Dawley rats (6 – 8 weeks) subjected to hypoxia (pulmonary hypertension model)	Kaempferol	25, 50 and 100 mg kg^{-1} day^{-1} (p.o.); 4 weeks	↓Pulmonary artery pressure and ventricular hypertrophy; ↓pAkt/Akt, p-GSK3β/t-GSK3β;↓*AKT1*, *CDK4*, *CCND1*, *PCNA* and *GSK3β* (mRNA); ↓PCNA, CDK2, CDK4, cyclin D1, cyclin A2;	↓Vascular remodeling and α-SMA	Zhang et al., 2023[51]

			↓Bcl2; no effect on Bax and Bax/Bcl-2; ↓caspase-3/β-actin		
		**Stilbenes**			
♂ C57BL6/J (6-7 weeks) implanted with micro pellets (80 ng bFGF and 160 ng VEGF) in the corneal pocket (neovascularization assay)	RSV	0.4 μg mL^{-1} in drinking water; 15 days		↓Corneal neovascularization	Bråkenhielm et al., 2001[52]
♂ Sprague Dawley rats (myocardial infarction model)	RSV	1 mg kg^{-1} day^{-1} in tap water; 14 days	↓Myocardial infarct size	↑Capillary density and regional myocardial blood flow; ↓VEGF and ↑HO-1 and Trx-1 (protein)	Kaga et al., 2005[53]
♂ Sprague Dawley rats underwent ligation of the LAD coronary artery (myocardial infarction model)	RSV	10 mg kg^{-1} (p.o); once a day for 7 days	↑Capillary density; ↓myocardial infarct area; ↑DNA binding activity of NF-κB and SP-1	↑VEGF and VEGFR2; ↑eNOS and iNOS	Fukuda et al., 2006[54]
♂ Sprague Dawley rats treated with STZ (diabetes model)	RSV (alone or together with L-NAME)	2.5 mg kg^{-1} day^{-1} (p.o.); 15 days	↓Glucose levels; Improved left ventricular function via reperfusion; ↓Infarct size and cardiomyocyte apoptosis; ↑p-AKT, p-eNOS, Trx-1, HO-1, and MnSOD activity (only with RSV); ↓p-AKT, p-eNOS, Trx-1, HO-1, and	↑VEGF (only with RSV) and ↓VEGF (only in combination)	Thirunavukkarasu et al., 2007[55]

			MnSOD activity (only in combination)		
Sprague-Dawley rats fed HCD underwent ischemia or LAD occlusion (MI model)	RSV (alone or together with pravastatin)	20 mg kg^{-1} day^{-1}; 2 weeks	↓TCh, TG, and LDL, whereas ↑HDL (synergic effect); improvement of cardiac function (LVDP, heart rate, d*p*/d*t*, coronary or aortic flow) and recovery of postischemic myocardial function (synergic effect); ↓Infarct size and apoptosis; ↑p-Akt/t-Akt and p-eNOS/t-eNOS; ↑β-catenin translocation	↑*Vegfa* level (mRNA) and capillary density (CD31)	Penumathsa et al., 2007[56]
♂ Sprague Dawley rats fed HCD underwent LAD (MI model)	RSV	20 mg kg^{-1} (p.o.) daily; 2 weeks	↓TCh, TG, LDL AND ↑HDL level; ↓Cav-1 and eNOS; ↑HO-1 and p-eNOS; improvement of left ventricular function	↑Capillary (CD31) and arteriolar density; ↑VEGF level	Penumathsa et al., 2008[57]
♂ Balb/c mice underwent cerebral focal ischemia surgery	RSV	50 mg kg^{-1} day^{-1} (gavage); 7 days	Improvement of neurological functions; ↓infarct size	↓MMP-2 and VEGF (mRNA and protein level); ↓vascular density in cortex (CD34 staining)	Dong et al., 2008[58]
♂ Yorkshire mini swine fed HCD underwent ameroid	RSV	100 mg kg^{-1} day^{-1}; 7 weeks	↓BMI, TCh, LDL, blood glucose, and SBP; no significant difference	No effect on blood vessel formation (CD31), endostatin, VE-cadherin, TSP-1, TIMP-2	Robich et al., 2010[59]

constrictor on the coronary artery (hypercholesterolemic chronic ischemia model)			in regional myocardial function and Rentrop collateral scores	MMP-2, MMP-9, or angiopoietin; ↑angiostatin	
Yorkshire mini swine fed HCD underwent constriction of the left circumflex artery (hypercholesterolemic chronic myocardial ischemia model)	RSV	100 mg kg^{-1} (p.o.) daily; 11 weeks	↓TCh; ↑Endothelium-dependent microvessel relaxation; ↑Tissue flow during stress; ↑Inferolateral function; ↑ NFκB, and p-Akt (Thr^{308})	No differences in capillary density (PECAM-1 staining); ↑VEGF, p-eNOS (Ser^{1177})	Robich et al., 2010[60]
♂ Yorkshire swine (8 weeks) fed HCD diet underwent left circumflex ameroid constrictor placement (hypercholesterolemic chronic ischemia model)	RSV (together with VEGF)	10 mg kg^{-1} (p.o.) daily; 7 weeks	↓Improvements in myocardial perfusion and arteriolar density; ↑Endothelium-dependent microvessel relaxation; ↑Akt and p-eNOs endothelial level; ↓Total protein oxidative stress and MPO level; ↓p-VE-cadherin and β-catenin	↓Arteriolar density (CD31 staining)	Chu et al., 2011[61]
Vldlr$^{-/-}$ (B6;129S7-*Vldlr$^{tm1Her/J}$* gene mutation) and wild type (pathological retinal	RSV	<u>First experiment:</u> weaned (Postnatal day (P): 21) mouse pups fed diets enriched with 2.4 g RSV kg^{-1}	↑*Rho* gene expression (mRNA); ↓*Gfap* gene expression (mRNA); no effect on *Cone opsin* gene expression (mRNA)	↓Neovascularization lesions observed at P30 and P60; ↓*Vegfa* gene expression (mRNA)	Hua et al., 2011[62]

neovascularization model)		chow/day; from P21 to P60 (39 days)<br><br><u>Second experiment:</u> 1g micronized RSV kg^{-1} b.w. day^{-1} (p.o.) administered to mouse pups from P10 to P30 (20 days)			
♂ Sprague-Dawley rats treated with STZ (diabetic nephropathy model)	RSV	20 mg kg^{-1} day^{-1} (p.o.);	↓Blood glucose, body, and kidney weight, albuminuria and creatinine clearance; ↓glomerular diameter, matrix index (ECM accumulation), and GBM thickness; ↓PAI-1, FN, CTGF, collagen IV, and TGF-β1	↓VEGF, ANG-1, Flk-1 level and ↑Tie-2 (protein); ↓*Angpt2* (mRNA)	Wen et al., 2013[63]
♂ Sprague Dawley rats (ischemia/reperfusion model)	RSV (alone or together with γ-tocotrienol) or longenivex[b]	5 mg kg^{-1} day^{-1} (p.o.) daily; 21 days	Modulation of the miRNAs expression pattern; improvement of cardiac function (aortic and coronary flow and LV development pressure), which was abolished by antagomir miRNA20b; ↓infarct size, ROS, and	↓VEGF and HIF1-α at the protein level (effect abolished using antagomir miRNA20b); differential expression of miR-21 and miR-20b (anti-angiogenic)	Mukhopadhyay et al., 2012[64]

			cardiomyocyte apoptosis		
♀ C57/BL6 mice (6 weeks) underwent laser-induced choroidal neovascularization	RSV or grape powder	Up to 25 mg kg^{-1} day^{-1} in drinking water; 16 or 24 days<br><br>50 mg kg^{-1} (p.o.) daily; 16 days		↓Blood vessel formation (*ad libitum* intake in water) or ↑blood vessel formation (daily oral gavage) measured by ICAM-2 staining	Kanavi et al., 2014[65]
Neonatal C57Bl/6 mice (P5–P17), oxygen-induced retinopathy model + transgenic Sirt1 knockout in retinal neurons (*Nes-Sirt1OE*) and vascular endothelial cells (*Tie2-Sirt1OE*)	RSV	400 mg kg^{-1} day^{-1} (gavage) from P5 to P17	↑retinal vaso-obliteration	No protection on pathological neovascularization related to *Sirt1* overexpression	Michan et al., 2014[66]
♀ Japanese macaques (*Macaca fuscata*; 9-13 years) fed WSD during pregnancy and effect in offspring (islet vascularization and innervation model)	RSV	0.37% (w/w) in WSD diet; consumed ad libitum from 3 months before the breeding season up to GD130	↑TH^+ fibers islet capillary area in utero	↑Islet capillary area, density, and size (PECAM-1); ↓$Ki67^+$/$PECAM\text{-}1^+$ in fetal islets; ↓*VEGFA, NOS3, KDR,* and *ANGPT2* (mRNA expression)	Pound et al., 2014[67]
♀ Sprague Dawley rats subjected to uterine horn transplantation (endometriosis model)	Polydatin (together with PEA)	10 mg kg^{-1} (gavage) daily; 14 days	↓Endometric lesion size; ↓uterine pain behaviors, tail-flick, and hot plate latency; ↓mast cell density and histological damage; ↓N-Tyr and PAR	↓VEGF, ICAM-1, and MMP-9 levels	Di Paola et al., 2016[68]

			formation in lesions; ↓NF-kB, MPO and NGF level; ↑Iκb-α level		
♂ C57/BL6 mice (5 weeks) to quantify CSCs after 7 days; ♂ C57/BL6 mice (6 weeks) subjected to injection with CSCs into the peri-ischemic area and subjected to left anterior descending coronary artery ligation (acute MI model)	RSV	2.5 mg kg^{-1} (p.o.) daily dissolved in PBS; 7 days (CSCs model) or 4 weeks (AMI model)	↑Number of endogenous Sca-1^{+} CSCs in heart tissue after 7 days; ↑Left ventricle function and ↓Cardiomyocyte apoptosis (after AMI); ↑SDF-1α level in the myocardium	↑Capillary density (CD31 staining) in the peri-ischemic myocardium; ↑VEGF level in the myocardium	Ling et al., 2017[69]
♂ Balb/c mice (6 to 7 weeks) treated with STZ (diabetes model)	RSV	200 mg kg^{-1} day^{-1} (p.o.); 14 days	↓Blood glucose levels after 2 hours of GTT (and it was maintained for 2 weeks); ↑insulin and BS-1 level; ↑endogenous β-cell mass and insulin/glucagon ratio (not significant); ↑Ki67 and insulin immunostaining (compared to total cell) at 3 days, but no differences at 14 days; ↓8-OH-dG and ROS	↑Vascular volume per islet graft ($C31^{+}$ staining)	Lee et al., 2018[70]

			level; ↓Cell death in isolated islets in hypoxic conditions; ↑*Sirt-1* and *Ins1* (mRNA)		
Diabetic mice (*db/db*) and their control littermates (*db/m*) and C57BL/6 mice (8 weeks) (diabetes model)	RSV	50 mg kg^{-1} day^{-1} (p.o.); 4 weeks	↓Fasting blood glucose and plasma insulin levels; ↑Hyperglycemia impaired endothelial cell proliferation (Ki67) and ↓Hyperglycemia-induced endothelial apoptosis (c-Caspase-3); SIRT1-, FoxO1- and c-Myc-mediated effects	↓Hyperglycemia-induced endothelial impairment (CD31 staining)	Huang et al., 2019[71]
♂ Wistar rats (6 – 8 weeks) treated with MCT (pulmonary hypertension model)	RSV	25 mg kg^{-1} day^{-1} in the drinking water; 28 days	↓Pulmonary vascular remodeling (H&E) and vascular wall thickness	↓Muscularized vessels; ↑miR-638 expression and ↓NR4A3 protein level	Liu et al., 2020[72]
♀ C57BL/6 (7 weeks) subjected to argon laser impact (induced choroidal neovascularization model)	RSV or Resvega[c]	RSV (20 µmol L^{-1}) or Resvega (12 µmol L^{-1}) (p.o.); 14 days	↓Lesion on optic nerve area; Differentially expressed proteins (positive regulation of organelles organization, JAK-STAT cascade; the adaptive immune system, and extrinsic signaling	Only Resvega: Down-regulation of differentially expressed proteins related to cell migration, vasculature development, blood vessel development, angiogenesis, blood vessel morphogenesis, and epithelial cell proliferation	Courtaut et al., 2021[73]

			pathways via death domain receptors; Up-regulation of proteins involved in metabolic process, localization, cellular component organization, cell proliferation; Detection of phase-II RSV metabolites, DH-RSV and RSV aglycone in retinal tissue		
C57BL/6JGpt mice (10 weeks) subjected to ligation of the left femoral artery (hind limb ischemia model)	RSV	40 mg kg^{-1} day^{-1} (gavage; diluted in 50% saline, 40% PEG400, and 10% DMSO); 7 days	↑Blood restoration and ↓apoptotic cells in gastrocnemius muscles; ↑RSV 4-glur concentration; ↑p-FoxO1/t-FoxO1	↑Vessel density (CD31) in gastrocnemius muscles	Fan et al., 2021[74]
			**Curcuminoids**		
♂ WNIN rats (3 months) treated with STZ (diabetic retina model)	Curc or turmeric (containing 1-2% curc)	0.002% or 0.01% Curc (w/w), or 0.5% turmeric (w/w) in AIN-93 diet; 8 weeks		↓VEGF and CML (mRNA and protein)	Mrudula et al., 2007[75]
♂ Swiss albino mice (6 – 8 weeks) treated with STZ (diabetes model) and i.v. transplanted with bone marrow cells	Curc	80 mg kg^{-1} (p.o.); 30 days	↓Blood glucose level; ↑organ regeneration; ↑insulin level and ↓fasting plasma glucose and caspases 3 and 9 levels; attenuation of b.w.	↑VEGF and PECAM-1 level	Arivazhagan et al., 2015[76]

			loss; the highest effects were observed when the animals received Curc together with bone marrow cells		
♂ C57BL/B6 mice (4 – 6 weeks) treated with STZ or sodium citrate and subjected to unilateral hindlimb artery devascularization (diabetic mouse and hindlimb ischemia model)	Curc	1000 mg kg^{-1} day^{-1} (p.o) in 300 µL olive oil once a day; 14 days	↑Blood flow recovery in diabetic hindlimb ischemia	↑Neovessel density (IB4-stained cells) in ischemic hindlimbs	You et al., 2017[77]
♂ Wistar rats (7–8 weeks) treated with $CaCl_2$ (periaortic application) to induce thoracic aortic aneurysm	Curc	100 mg kg^{-1} day^{-1} (p.o.); 28 days	↓Thoracic aortic diameter; ↓Elastin fragmentation and preserved the elastic laminar wave; ↓inflammatory cell infiltration (CD68-positive cells) in the aorta; ↓MCP-1 and TNF-α	↓Number of microvessels in the aortic wall; ↓VEGF in the aorta, but no effect in the heart; ↓VCAM-1 and ICAM-1	Li et al., 2017[78]
♂ Wistar rats treated with STZ (diabetes model)	Curc (alone or together with PBM)	40 mg kg^{-1} day^{-1} (p.o.); 6 days per week for 15 days	↑Inflammatory response modulation in terms of macrophages, neutrophils, and	↑Angiogenesis; ↓*Hif-1a* (only in combination at day 4) and ↑*Fgf2* gene expression (mRNA)	Amini et al., 2019[79]

			fibroblasts; ↑*Cxcl12* gene expression by Curc or PBM at day 4; ↓*Cxcl12* gene expression by Curc together with PBM		
♂ Balb/c mice (14 weeks) subjected to ligation and excision of the femoral artery (hindlimb ischemia model)	Curc	1,000 mg kg^{-1} day^{-1} (p.o.); 2 weeks	↑Perfusion recovery; ↑miR-93 expression in ischemic muscle tissue	↑Capillary density (CD31 staining)	Zhang et al., 2019[80]
♂ C57BL/6 mice whose inferior vena cava was subjected to a 30-sec application of a narrow strip pre-soaked with $FeCl_3$ (venous thrombosis model)	Curc	1,000 mg kg^{-1} day^{-1} (p.o.); 14 days	↓Weight of thrombus; ↓*miR-499* and *miR-21* expression; ↑*miR-126* and *miR-150* expression	↑Vessel density (H&E staining); ↑VEGF and Ang-1 level (mRNA and protein)	Wang et al., 2021[81]
♂ C57BL/6 mice (6 weeks) treated with STZ (diabetes model)	Curc	100 mg kg^{-1} day^{-1} (p.o.); 8 weeks	Curc prevented diabetes-induced bone loss	↑Vessel formation (CD31 staining)	Fan et al., 2022[82]
♂ Wistar rats treated with L-NAME (hypertension model)	HCC	20, 40 and 80 mg kg^{-1} day^{-1} (p.o.); 3 weeks	↓SBP, ROS production, and MDA level (dose-dependent effect); ↑SOD activity, eNOS, p-eNOS, NO, p-NF-κB, NF-κB and TNF-α level (significant	↑Vascular response and improvement of endothelial dysfunction; ↓ICAM-1 and VCAM-1; ↓media thickness/lumen ratio in thoracic aorta sections and cross-sectional area (dose-	Panthiya et al., 2022[83]

			at the highest concentration); ↓p-ERK/t-ERK, p-JNK/t-JNK and p-p38/t-p38 (at the highest concentration); no effect of HCC (80 mg kg^{-1}) in the absence of L-NAME	dependent effect); ↓MMP-9, TGF-β1, OPN and COL1 level; no effect on α-SMA level; no effect of HCC (80 mg kg^{-1}) in the absence of L-NAME	
♂ Sprague Dawley rats (4 – 6 weeks) treated with STZ (diabetic foot ulcer model)	Curc	300 mg kg^{-1} day^{-1} (i.g.); 12 days	↑Wound recovery, SOD, Ki-67, Bcl-2 and K14; ↓MDA, Bax and caspase-3; effects exerted via inhibition of miR-152-3p and activation of FBN1/TGF-β pathway in DUF	↑Angiogenesis (H&E)	Cao et al., 2024[84]

Abbreviations: 8-OH-dG: 8-hydroxy-2'-deoxyguanosine; **8-PN:** 8-prenylnaringenin; **ACSL4:** Acyl-CoA synthetase long chain family member 4 or long-chain fatty-acid coenzyme A ligase 4; **AGE:** Advanced Glycation End products; **Akt:** Protein kinase B**; AMD:** age-related macular degeneration; **AMI:** Acute myocardial infarction; **Ang-1:** angiopoietin-1; **Apo-E:** apolipoprotein E; **AST:** aspartate aminotransferase; **ATF-6:** activating transcription factor-6; **ATM:** ataxia-telangiectasia mutated; **BAT:** brown adipose tissue; **BBB:** blood brain barrier; **bFGF:** basic fibroblasts growth factor; **BMI:** body mass index; **BDNF:** brain neurotrophic factor; **BS:** Blood sugar level; **b.w.:** body weight; **CAT:** catalase; **CD31/PECAM1:** platelet endothelial cell adhesion molecule 1; **CDK:** Cyclin-dependent kinase 2; **CIA:** collagen induced arthritis; **CKMB:** creatinine phosphokinase myocardial bodies; **CML:** carboxymethyl lysine; **COL1:** collagen type-1; **COX-2:** cyclooxygenase-2; **CREB**: cAMP Response Element-Binding protein; **CRP:** C-reactive protein; **CSCs:** cardiac stem cells; **CTGF:** connective tissue growth factor; **Curc:** curcumin; **CYR61:** cysteine-rich angiogenic inducer 61; **DAB2:** Disabled 2; **DBP:** diastolic blood pressure; **DHA:** docosahexaenoic acid; **DH-RSV:** dihydro-resveratrol; **EA:** ellagic acid; **ECM:** extracellular matrix; **EGCG:** epigallocatechin gallate; **EPA:** eicosapentaenoic acid; **ERK:** extracellular signal-regulated kinases; **ESM-1:** endothelial cell specific molecule 1; **FAS:** fatty acid synthase; **FLC:** Flax lignan concentrate; **FLK-1**: Fetal Liver Kinase-1 (also known as VEGFR-2; **FN:** fibronectin; **FoxO1:** forkhead box class-O1; **FS:** flaxseed; **GBM:** glomeluar basement; **GD:** gestational day; **GFAP:** Glial fibrillary acidic protein; **GLUT:** Glucose transporter; **GDNF:** Glial Cell-Derived Neurotrophic Factor; **GPX:** glutathione peroxidase; **GSH:** glutathione; **GSPE:** grape seed proanthocyanidin extract; **GST:** Glutathione S-transferase; **GTT:** glucose tolerance test; **Hb:** haemoglobin; **HbA_{1c}:** glycated

haemoglobin or hemoglobin A_{1c}; **HCC:** Hexahydroxycurcumin; **HCD:** high cholesterol diet; **HDL:** high density lipoprotein; **HFD:** high fat diet; **HGF:** hepatocyte growth factor; **HIF-1α:** hipoxia inducible factor 1 alpha; **HK**: Hexokinase; **HO-1:** heme oxidase 1; **HSP90:** heat shock protein 90; **H&E**: haematoxylin and eosin; **Iba-1:** ionized calcium binding adaptor molecule-1; **sICAM-1:** soluble intracelular Adhesion Molecule 1; **ICAM:** intracelular adhesion molecule; **IGF-1:** insulin growth factor 1; **IκB:** Inhibitor of kappa B; **iNOS:** inducible nitric oxide synthase; **IR:** insulin receptor; **JNK:** c-Jun NH2-terminal kinase; **LAD:** left artery descending coronary artery; **LDH:** lactate dehydrogenase; **LDL:** low density lipoprotein; **L-NAME:** NG-nitroarginine methyl ester; **LPO:** lipid peroxidation; **LVAW**: Left ventricular anterior wall; **LVDP:** left ventricular developed pressure; **LVID:** Left ventricular internal dimension; **LVPW**: Left ventricular posterior wall; **MABP:** mean arterial blood pressure; **MBP:** mean blood pressure; **MCAO:** middle cerebral artery occlusion; **MCP-1:** monocyte chemoattractant protein 1; **MCT:** monocrotaline; **MDA:** malondialdehide; **MEK:** mitoen-activated protein kinase kinase; **MI:** myocardial infarction; **miR:** microRNA; **MMP:** matrix metalloproteinase; **MnSOD:** manganese superoxide dismutase; **mPAP:** mean pulmonary artery pressure; **MPO:** myeloperoxidase; **N-AGM**: N-acetylglucosamine; **n.d.:** not describe**d; NF-kB**: Nuclear factor kappa-light-chain-enhancer of activated B cells; **NGF:** nerve growth factor; **NO:** nitric oxide; **NP-1:** neurophilin-1; **NR4A3:** nuclear receptor 4A3; **Nrf2:** nuclear factor erythroid 2-related factor 2; **NRP1:** neuropilin 1; **N-Tyr:** nitrotyrosine; **OPN:** osteopontin; **ORAC:** oxygen radical absorbance capacity; **p-:** phosphorylated; **p38:** mitogen-activated protein kinases; **PAI-1:** plasminogen activator inhibitor-1; **PAR:** Poly (ADP-ribose); **PCNA:** proliferating cell nuclear antigen; **PDK:** Pyruvate Dehydrogenase Kinase; **PEA:** palmitoylethanolamide; **PECAM-1:** platelet endothelial cell adhesion molecule; **PFK**: Phosphofructokinase-1; **PFKFB3:** phosphofructokinase-2/fructose-2,6-biphosphatase 3; **PGG:** penta-*O*-galloyl-β-D-glucose; **PBM:** photobiomodulation; **PDGF-BB**: Platelet-derived growth factor subunit B; **PI3K:** phosphatidylinositol 3-kinase; **PKC-β:** protein kinase C beta; **PPAR-γ:** peroxisome proliferatror-activated receptor gamma; **Quer:** quercetin; **RAGE:** receptor for advanced glycation end products: **RANKL:** receptor activator of nuclear factor kappa beta (NF-κB) ligand; **Rho:** rhodopsin; **ROS:** reactive oxygen species; **RSV:** resveratrol; **RWPC:** red wine (poly)phenolic compounds; **RVHI:** right ventricular hypertrophy index; **RVP**: retinal vascular permeability; **RVSP:** right ventricular systolic pressure; **SBP:** systolic blood pressure; **Sca-1:** stem cell antigen 1; **SDF-1α:** stromal cell-derived factor; **SIRT:** sirtuin; **αSMA:** alpha smooth muscle actin; **SM**: smooth muscle protein; **SMAD:** suppressor of mothers against decapentaplegic; **SOD:** superoxide dismutase; **Src:** proto-oncogen tyrosine-protein kinase Src; **STZ:** streptozotocin; **TCh:** total cholesterol; **TG:** triglycerides; **TGF-β1:** transforming growth factor beta 1; **TH:** tyrosine hydroxylase; **Tie2:** angiopoietin-1 receptor; **TIMP:** tissue inhibitor of metalloproteinases: **TLR-4:** toll-like receptor; **TNC:** tenascin C; **TNF-α:** tumor necrosis factor alpha; **TRAP:** tartrate-resistant acid phosphatase; **Trx-1:** thioredoxin-1; **TSP:** thrombospondin; **uPA:** urokinase plasminogen activator; **Uro-A:** urolithin-A; **VEGF:** vascular endothelial growth factor; **VEGFR2:** vascular endothelial growth factor receptor 2; **VLDL:** very low density lipoprotein; **vWF:** von Willebrand protein; **WAT:** white adipose tissue; **WSD:** western-style diet; **XBP-1:** X-box binding protein 1; **XN:** Xanthohumol

[a]**Provinols™:** proanthocyanidins: 480 mg kg^{-1}; total anthocyanins: 61 mg kg^{-1}; free anthocyanins: 19 mg kg^{-1}; catechins: 38 mg kg^{-1}; hydroxycinnamic acids: 18 mg kg^{-1}; flavonols: 14 mg kg^{-1}; polymeric tannins: 370 mg kg^{-1}.
[b]**Longenivex:** Trans RSV from *Polygonum cuspidatum*, 100 mg Quer (micronized, microencapsulated), 25 mg rice bran calcium phytate (IP6), 75 mg Vit D3, 1000 IU rice bran ferulic acid.

[c]**Resvega:** 30 mg *trans*-RSV + 240 mg Vit. C; 30 mg E; 12.5 mg Zn; 1 mg Cu; 380 mg EPA; 190 mg DHA; 10 mg lutein; 2 mg zeaxanthin.

Acknowledgments

This work was supported by the Ramón y Cajal grant (RyC2021-032111-I) and CNS2022-135253 grant funded by the MICIU/AEI/10.13039/501100011033 and by the "European Union NextGenerationEU/PRTR" program. It was also supported by the grants PID2022-136419OB-I00 and PID2022-136915NA-I00 funded by MICIU/AEI/10.13039/501100011033 and "ERDF A way of making Europe" by the European Union, by the grant 22030/PI/22 funded by the Programa Regional de Fomento de la Investigación Científica y Técnica (Plan de Actuación 2022) de la Fundación Séneca-Agencia de Ciencia y Tecnología de la Región de Murcia, Spain, and by the AGROALNEXT program (MICIU, PRTR-C17.I1, Spain) with funding from the European Union NextGenerationEU (PRTR-C17.I1) and Fundación Séneca (Comunidad Autónoma Región de Murcia, Spain). CS was supported by NIH award R35GM144091 by the National Institute of General Medical Sciences. AV-P was supported by a predoctoral contract associated with the grant PID2022-136915NA-I00 funded by MCIN/AEI/10.13039/501100011033 and "ERDF A way of making Europe" by the European Union.

References

[1] C. Baron-Menguy, A. Bocquet, A.-L. Guihot, D. Chappard, M.-J. Amiot, R. Andriantsitohaina, L. Loufrani, D. Henrion, *FASEB J.* **2007**, *21*, 3511.

[2] A. Mauray, C. Felgines, C. Morand, A. Mazur, A. Scalbert, D. Milenkovic, *Nutr Metab Cardiovasc Dis* **2012**, *22*, 72.

[3] M.-K. Kang, S. S. Lim, J.-Y. Lee, K. M. Yeo, Y.-H. Kang, *PLOS ONE* **2013**, *8*, e79823.

[4] S. Ouyang, W. Chen, Z. Gaofeng, L. Changcheng, T. Guoping, Z. Minyan, L. Yang, Y. Min, J. Luo, *Mol. Med. Rep.* **2021**, *23*, 1.

[5] F. Zhao, X. Gao, X. Ge, J. Cui, X. Liu, *Bioengineered* **2021**, *12*, 9266.

[6] T. Fushimi, S. Oyama, R. Koizumi, Y. Fujii, N. Osakabe, *J. Clin. Biochem. Nutr.* **2023**, *72*, 132.

[7] L. M. Cryan, L. Bazinet, K. A. Habeshian, S. Cao, J. Clardy, K. A. Christensen, M. S. Rogers, *J. Med. Chem.* **2013**, *56*, 1940.

[8] G. Raghu, C. Akileshwari, V. S. Reddy, G. B. Reddy, *J. Food Sci. Technol.* **2017**, *54*, 2411.

[9] L. Hu, X. Chen, S. Qiu, J. Yang, H. Liu, J. Zhang, D. Zhang, F. Wang, *Am. J. Chin. Med.* **2020**, *48*, 1005.

[10] K. Lu, O. H. Iwenofu, R. Mitra, X. Mo, P. S. Dasgupta, S. Basu, *Arthritis. Res. Ther.* **2020**, *22*, 273.

[11] N. Ghosh, A. Das, N. Biswas, S. Gnyawali, K. Singh, M. Gorain, C. Polcyn, S. Khanna, S. Roy, C. K. Sen, *Sci. Rep.* **2020**, *10*, 20184.

[12] Z. Feng, J. Chen, P. Yuan, Z. Ji, S. Tao, L. Zheng, X. Wei, Z. Zheng, B. Zheng, B. Chen, J. Chen, F. Zhao, *Front. Pharmacol.* **2022**, *13*, 806284.

[13] F. Huang, L. Luo, Y. Wu, D. Xia, F. Xu, J. Gao, J. Shi, Q. Gong, *Phytother. Res.* **2022**, *36*, 2940.

[14] Y. Cao, R. Cao, *Nature* **1999**, *398*, 381.

[15] N. Kavantzas, A. Chatziioannou, A. E. Yanni, D. Tsakayannis, D. Balafoutas, G. Agrogiannis, D. Perrea, *Vascul. Pharmacol.* **2006**, *44*, 461.

[16] I. Ramirez-Sanchez, L. Nogueira, A. Moreno, A. Murphy, P. Taub, G. Perkins, G. Ceballos, M. Hogan, M. Malek, F. Villarreal, *J. Cardiovasc. Pharmacol.* **2012**, *60*, 429.

[17] H. S. Lee, J.-H. Jun, E.-H. Jung, B. A. Koo, Y. S. Kim, *Molecules* **2014**, *19*, 12150.

[18] J.-P. Hao, H. Shi, J. Zhang, C.-M. Zhang, Y.-M. Feng, L.-Y. Qie, M. Dong, X. Ji, *Eur Rev Med Pharmacol. Sci.* **2018**, *22*, 6999.

[19] B. Kumar, S. K. Gupta, B. P. Srinivasan, T. C. Nag, S. Srivastava, R. Saxena, *Vascul. Pharmacol.* **2012**, *57*, 201.

[20] X. Shi, S. Liao, H. Mi, C. Guo, D. Qi, F. Li, C. Zhang, Z. Yang, *Molecules* **2012**, *17*, 12868.

[21] A. D. Kandhare, P. Ghosh, S. L. Bodhankar, *Chem. Biol. Interact.* **2014**, *219*, 101.

[22] L. Cheng, T. Chen, Q. Tu, H. Li, Z. Feng, Z. Li, D. Lin, *Oncotarget* **2017**, *8*, 94142.

[23] R. Costa, I. Rodrigues, L. Guardão, J. Q. Lima, E. Sousa, R. Soares, R. Negrão, *Mol. Nutr. Food Res.* **2017**, *61*.

[24] P. Maneesai, S. Bunbupha, P. Potue, T. Berkban, U. Kukongviriyapan, V. Kukongviriyapan, P. Prachaney, P. Pakdeechote, *Nutrients* **2018**, *10*, 1549.

[25] G. Haddadi, A. Abbaszadeh, M. A. Mosleh-Shirazi, M. A. Okhovat, A. Salajeghe, Z. Ghorbani, *J. Cancer Res. Ther.* **2018**, *14*, S1098.

[26] W. Li, A. D. Kandhare, A. A. Mukherjee, S. L. Bodhankar, *EXCLI. J.* **2018**, *17*, 399.

[27] R. Kapoor, V. K. Sirohi, K. Gupta, A. Dwivedi, *J. Nutr. Biochem.* **2019**, *70*, 215.

[28] X. Yu, Z. Liu, Y. Yu, C. Qian, Y. Lin, S. Jin, L. Wu, S. Li, *Phytother. Res.* **2024**, *38*, 1478.

[29] M. E. Mehrabadi, Z. Salemi, S. Babaie, M. Panahi, *Can. J. Diabetes* **2018**, *42*, 639.

[30] S. V. Penumathsa, S. Koneru, M. Thirunavukkarasu, L. Zhan, K. Prasad, N. Maulik, *J Pharmacol. Exp. Ther.* **2007**, *320*, 951.

[31] C. H. Lin, C. H. Li, P. L. Liao, L. S. Tse, W. K. Huang, H. W. Cheng, Y. W. Cheng, *Br. J. Pharmacol.* **2013**, *168*, 920.

[32] G. C. Vicente, A. M. Correia-Santos, A. Suzuki, L. G. Coca Velarde, M. A. Chagas, G. T. Boaventura, *J. Sci. Food Agric.* **2015**, *95*, 2973.

[33] A. E. Ghule, A. D. Kandhare, S. S. Jadhav, A. A. Zanwar, S. L. Bodhankar, *Int. Immunopharmacol.* **2015**, *28*, 751.

[34] E. Nahari, M. Razi, *Acta Histochem.* **2018**, *120*, 757.

[35] A. M. Stolf, C. Campos Cardoso, H. de Morais, C. E. Alves de Souza, L. A. Lomba, A. P. Brandt, J. P. Agnes, F. C. Collere, C. M. Galindo, C. R. Corso, K. M. Spercoski, R. Locatelli Dittrich, A. R. Zampronio, S. M. S. C. Cadena, A. Acco, *Biomed. Pharmacother.* **2018**, *108*, 232.

[36] Q. Deng, Y. Wang, C. Wang, B. Ji, R. Cong, L. Zhao, P. Chen, X. Zang, F. Lu, F. Han, F. Huang, *Food Funct.* **2018**, *9*, 2469.

[37] X.-M. Hu, M.-M. Zhou, X.-M. Hu, F.-D. Zeng, *Acta Pharmacol. Sin.* **2005**, *26*, 1454.

[38] N. Xu, L. Zhang, J. Dong, X. Zhang, Y.-G. Chen, B. Bao, J. Liu, *Mol. Nutr. Food Res.* **2014**, *58*, 1258.

[39] J. Wang, J. Tan, J. Luo, P. Huang, W. Zhou, L. Chen, L. Long, L.-M. Zhang, B. Zhu, L. Yang, D. Y. B. Deng, *J. Nanobiotechnology* **2017**, *15*, 18.

[40] Z. Kong, Q. Shen, J. Jiang, M. Deng, Z. Zhang, G. Wang, *Ann. Transl. Med.* **2019**, *7*, 639.

[41] D. Li, J. Ma, L. Wang, S. Xin, *J. Cardiovasc. Pharmacol.* **2020**, *75*, 229.

[42] L. Long, Y. Li, S. Yu, X. Li, Y. Hu, T. Long, L. Wang, W. Li, X. Ye, Z. Ke, H. Xiao, *J. Diabetes Res.* **2019**, *2019*, 4875421.

[43] X. Ma, Y. Lin, yingying Liu, W. Li, J. He, M. Fang, D. Lin, *Front. Pharmacol.* **2021**, *12*, 625733.

[44] X. Lin, F. Xu, K.-W. Zhang, W.-X. Qiu, H. Zhang, Q. Hao, M. Li, X.-N. Deng, Y. Tian, Z.-H. Chen, A.-R. Qian, *Front. Cell Dev. Biol.* **2022**, *10*, 796227.

[45] X. Wang, L. Cui, Y. Wang, Z. Zeng, H. Wang, L. Tian, J. Guo, Y. Chen, *Eur. J. Pharmacol.* **2024**, *978*, 176786.

[46] N. M. Al-Rasheed, L. M. Faddah, A. M. Mohamed, N. A. Abdel Baky, N. M. Al-Rasheed, R. A. Mohammad, *J. Oleo Sci.* **2013**, *62*, 961.

[47] R. Huang, Z. Shi, L. Chen, Y. Zhang, J. Li, Y. An, *Eur. J. Pharm.* **2017**, *814*, 151.

[48] D. J. Perdicaro, C. Rodriguez Lanzi, J. Gambarte Tudela, R. M. Miatello, P. I. Oteiza, M. A. Vazquez Prieto, *J. Nutr. Biochem.* **2020**, *79*, 108352.

[49] L. Wang, H. Wu, L. Xiong, X. Liu, N. Yang, L. Luo, T. Qin, X. Zhu, Z. Shen, H. Jing, J. Chen, *BioMed Res. Int.* **2020**, *2020*, 9485398.

[50] G.-R. Chai, S. Liu, H.-W. Yang, X.-L. Chen, *Neural. Regen. Res.* **2021**, *16*, 1344.

[51] X. Zhang, Z. Yang, S. Su, X. Nan, X. Xie, Z. Li, D. Lu, *Toxicol. Appl. Pharmacol.* **2023**, *466*, 116478.

[52] E. Bråkenhielm, R. Cao, Y. Cao, *FASEB J.* **2001**, *15*, 1798.

[53] S. Kaga, L. Zhan, M. Matsumoto, N. Maulik, *J. Mol. Cell Cardiol.* **2005**, *39*, 813.

[54] S. Fukuda, S. Kaga, L. Zhan, D. Bagchi, D. K. Das, A. Bertelli, N. Maulik, *Cell Biochem. Biophys.* **2006**, *44*, 43.

[55] M. Thirunavukkarasu, S. V. Penumathsa, S. Koneru, B. Juhasz, L. Zhan, H. Otani, D. Bagchi, D. K. Das, N. Maulik, *Free Radic. Biol. Med.* **2007**, *43*, 720.

[56] S. V. Penumathsa, M. Thirunavukkarasu, S. Koneru, B. Juhasz, L. Zhan, R. Pant, V. P. Menon, H. Otani, N. Maulik, *J. Mol. Cell Cardiol.* **2007**, *42*, 508.

[57] S. V. Penumathsa, S. Koneru, S. M. Samuel, G. Maulik, D. Bagchi, S.-F. Yet, V. P. Menon, N. Maulik, *Free Radic. Biol. Med.* **2008**, *45*, 1027.

[58] W. Dong, N. Li, D. Gao, H. Zhen, X. Zhang, F. Li, *J. Vasc. Surg.* **2008**, *48*, 709.

[59] M. P. Robich, L. M. Chu, M. Chaudray, R. Nezafat, Y. Han, R. T. Clements, R. J. Laham, W. J. Manning, M. A. Coady, F. W. Sellke, *Surgery* **2010**, *148*, 453.

[60] M. P. Robich, R. M. Osipov, R. Nezafat, J. Feng, R. T. Clements, C. Bianchi, M. Boodhwani, M. A. Coady, R. J. Laham, F. W. Sellke, *Circulation* **2010**, *122*, S142.

[61] L. M. Chu, M. P. Robich, A. D. Lassaletta, J. Feng, R. J. Laham, T. Burgess, R. T. Clements, F. W. Sellke, *Surgery* **2011**, *150*, 390.

[62] J. Hua, K. I. Guerin, J. Chen, S. Michán, A. Stahl, N. M. Krah, M. R. Seaward, R. J. Dennison, A. M. Juan, C. J. Hatton, P. Sapieha, D. A. Sinclair, L. E. H. Smith, *Invest. Ophthalmol. Vis. Sci.* **2011**, *52*, 2809.

[63] D. Wen, X. Huang, M. Zhang, L. Zhang, J. Chen, Y. Gu, C.-M. Hao, *PLoS One* **2013**, *8*, e82336.

[64] P. Mukhopadhyay, S. Das, M. K. Ahsan, H. Otani, D. K. Das, *J. Cell Mol. Med.* **2012**, *16*, 2504.

[65] M. R. Kanavi, S. Darjatmoko, S. Wang, A. A. Azari, M. Farnoodian, J. D. Kenealey, P. R. van Ginkel, D. M. Albert, N. Sheibani, A. S. Polans, *Molecules* **2014**, *19*, 17578.

[66] S. Michan, A. M. Juan, C. G. Hurst, Z. Cui, L. P. Evans, C. J. Hatton, D. T. Pei, M. Ju, D. A. Sinclair, L. E. H. Smith, J. Chen, *PLoS One* **2014**, *9*, e85031.

[67] L. D. Pound, S. M. Comstock, K. L. Grove, *Am. J. Physiol. Endocrinol. Metab.* **2014**, *307*, E115.

[68] R. Di Paola, R. Fusco, E. Gugliandolo, R. Crupi, M. Evangelista, R. Granese, S. Cuzzocrea, *Front. Pharmacol.* **2016**, *7*, 382.

[69] L. Ling, S. Gu, Y. Cheng, *Mol. Med. Rep.* **2017**, *15*, 1188.

[70] E.-M. Lee, I. Park, Y.-J. Lee, Y.-H. You, J.-W. Kim, M.-J. Kim, Y.-B. Ahn, P. Kim, S.-H. Ko, *Islets* **2018**, *10*, 25.

[71] X. Huang, J. Sun, G. Chen, C. Niu, Y. Wang, C. Zhao, J. Sun, H. Huang, S. Huang, Y. Liang, Y. Shen, W. Cong, L. Jin, Z. Zhu, *Front. Pharmacol.* **2019**, *10*, 421.

[72] Y.-Y. Liu, W.-Y. Zhang, C.-G. Wang, J.-A. Huang, J.-H. Jiang, D.-X. Zeng, *Microvasc. Res.* **2020**, *130*, 103988.

[73] F. Courtaut, V. Aires, N. Acar, L. Bretillon, I. C. Guerrera, C. Chhuon, J.-P. Pais de Barros, C. Olmiere, D. Delmas, *Int. J. Mol. Sci.* **2021**, *22*, 11023.

[74] D. Fan, C. Liu, Z. Guo, K. Huang, M. Peng, N. Li, H. Luo, T. Wang, Z. Cen, W. Cai, L. Gu, S. Chen, Z. Li, *Molecules* **2021**, *26*, 7528.

[75] T. Mrudula, P. Suryanarayana, P. N. B. S. Srinivas, G. B. Reddy, *Biochem. Biophys. Res. Commun.* **2007**, *361*, 528.

[76] A. Arivazhagan, S. Krishna, S. Yadav, H. R. Shah, P. Kumar, R. K. Ambasta, *J. Diabetes* **2015**, *7*, 473.

[77] J. You, J. Sun, T. Ma, Z. Yang, X. Wang, Z. Zhang, J. Li, L. Wang, M. Ii, J. Yang, Z. Shen, *Stem Cell Res. Ther.* **2017**, *8*, 182.

[78] X. Li, Q. Fang, X. Tian, X. Wang, Q. Ao, W. Hou, H. Tong, J. Fan, S. Bai, *Mol. Med. Rep.* **2017**, *16*, 4455.

[79] A. Amini, H. Soleimani, M.-A. Abdollhifar, A. Moradi, S. K. Ghoreishi, S. Chien, M. Bayat, *J Cell Biochem.* **2019**, *120*, 17994.

[80] J. Zhang, Q. Wang, G. Rao, J. Qiu, R. He, *Exp. Ther. Med.* **2019**, *17*, 798.

[81] T. Wang, R. Guan, F. Xia, J. Du, L. Xu, *Microvasc. Res.* **2021**, *136*, 104148.

[82] D. Fan, J. Lu, N. Yu, Y. Xie, L. Zhen, *Evid. Based Complement. Alternat. Med.* **2022**, *2022*, 4974343.

[83] L. Panthiya, J. Tocharus, A. Onsa-ard, W. Chaichompoo, A. Suksamrarn, C. Tocharus, *Biochim. Biophys Acta (BBA) – Mol. Basis Dis.* **2022**, *1868*, 166317.

[84] M. Cao, Z. Duan, X. Wang, P. Gong, L. Zhang, B. Ruan, *Mol. Biotechnol.* **2024**, *66*, 1266.
